# Anesthesia and Monitoring in Small Laboratory Mammals Used in Anesthesiology, Respiratory and Critical Care Research: A Systematic Review on the Current Reporting in Top-10 Impact Factor Ranked Journals

**DOI:** 10.1371/journal.pone.0134205

**Published:** 2015-08-25

**Authors:** Christopher Uhlig, Hannes Krause, Thea Koch, Marcelo Gama de Abreu, Peter Markus Spieth

**Affiliations:** Pulmonary Engineering Group, Department of Anesthesiology and Intensive Care Therapy, University Hospital Dresden, Dresden, Technische Universität Dresden, Germany; Centre Hospitalier de l'Université Laval, CANADA

## Abstract

**Rationale:**

This study aimed to investigate the quality of reporting of anesthesia and euthanasia in experimental studies in small laboratory mammals published in the top ten impact factor journals.

**Methods:**

A descriptive systematic review was conducted and data was abstracted from the ten highest ranked journals with respect to impact factor in the categories ‘Anesthesiology’, ‘Critical Care Medicine’ and ‘Respiratory System’ as defined by the 2012 Journal Citation Reports. Inclusion criteria according to PICOS criteria were as follows: 1) population: small laboratory mammals; 2) intervention: any form of anesthesia and/or euthanasia; 3) comparison: not specified; 4) primary outcome: type of anesthesia, anesthetic agents and type of euthanasia; secondary outcome: animal characteristics, monitoring, mechanical ventilation, fluid management, postoperative pain therapy, animal care approval, sample size calculation and performed interventions; 5) study: experimental studies. Anesthesia, euthanasia, and monitoring were analyzed per performed intervention in each article.

**Results:**

The search yielded 845 articles with 1,041 interventions of interest. Throughout the manuscripts we found poor quality and frequency of reporting with respect to completeness of data on animal characteristics as well as euthanasia, while anesthesia (732/1041, 70.3%) and interventions without survival (970/1041, 93.2%) per se were frequently reported. Premedication and neuromuscular blocking agents were reported in 169/732 (23.1%) and 38/732 (5.2%) interventions, respectively. Frequency of reporting of analgesia during (117/610, 19.1%) and after painful procedures (38/364, 10.4%) was low. Euthanasia practice was reported as anesthesia (348/501, 69%), transcardial perfusion (37/501, 8%), carbon dioxide (26/501, 6%), decapitation (22/501, 5%), exsanguination (23/501, 5%), other (25/501, 5%) and not specified (20/501, 4%, respectively.

**Conclusions:**

The present systematic review revealed insufficient reporting of anesthesia and euthanasia methods throughout experimental studies in small laboratory mammals. Specific guidelines for anesthesia and euthanasia regimens should be considered to achieve comparability, quality of animal experiments and animal welfare. These measures are of special interest when translating experimental findings to future clinical applications.

## Introduction

Animal models in biomedical research are important to improve *in vivo* understanding of the physiology of living organisms as well as the pathophysiology of diseases [1]. Despite recent advances in computer simulation [1–3] and other alternative methods [4,5], animal experiments are still necessary to test new treatment strategies in pre-clinical research [6].

Among the different species available, small mammals are used most frequently for laboratory research. In this systematic review ‘small laboratory mammals’ are defined as members of the clade glires (rodents and lagomorpha). Reasons for the frequent use of small laboratory mammals are low maintenance costs, short reproduction time and availability of transgenic models [5,6]. Experiments conducted in rodents often require anesthesia and result in euthanasia of the animal at the end of the experiment [7].

Anesthesia in small animals might be challenging. Specific anesthesia-related problems are mainly caused by the size of the animal. The placement of invasive hemodynamic monitoring is often difficult and may cause severe complications. Furthermore, special and expensive equipment such as surgical instruments, microscopes or mechanical ventilators for small laboratory mammals can be necessary. Hypothermia, while not exclusive to small mammals, is a special concern in these animals and an important cause of unintended death under anesthesia [8–10]. High metabolic rates and difficulties monitoring of respiratory [11] and cardiovascular function reliably [11,12] contribute to the complexity of the anesthetic management.

Various drugs are used in experimental research. They differ in part or total from human anesthesia practice with respect to the substances themselves, dosages administered, routes of administration and form of application [6]. Furthermore, the usage of analgesic drugs during and/or after surgical procedures in laboratory animals is uncommon [7,13], but has increased over time [14]. This lack of comparability to human clinical practice contributes to concerns raised by the society and fellow researchers regarding bioethical aspects [15–17] as well as translation of experimental findings back into clinical practice.

The Animal Research: Reporting of In Vivo Experiments (ARRIVE) guidelines [18] recommend detailed description of animal characteristics, experimental procedures including anesthesia and euthanasia, as well as general aspects, such as institutional animal care and use committee approval or sample size calculation to be included in scientific publications. In spite of the essential role of complete reporting of experimental parameters for reproducibility and translation of experimental findings into phase I clinical trials, Joffe *et al*. report, after analyzing articles published over six month in three journals from the field of critical care medicine, that the methodological quality of reporting in animal experiments in the field of critical care is poor [17].

Considering the limited scope of this previous work, we performed a systematic review of the top ten journals in each of the categories ‘Anesthesiology’, ‘Respiratory System’ and ‘Critical Care Medicine’ reporting animal experimental studies. Our aim was to evaluate the frequency and quality of reporting of anesthesia, euthanasia, including animal characteristics, postoperative pain therapy, monitoring, mechanical ventilation, animal care approval, sample size calculation and performed interventions in experimental studies with small laboratory mammals, over a one year period on a broad data base. Additionally to rodents, we included lagomorpha as they are widely used in experimental research. They are also closely related to rodents and investigators face similar challenges when handling and taking care of the animals.

We hypothesized that 1) the frequency of reporting experiments involving small laboratory mammals published in the top ten journals in the categories ‘Anesthesiology’, ‘Respiratory System’ and ‘Critical Care Medicine’ over a one year period is high while 2) the quality of reporting in these studies is poor regarding the provision of data on anesthesia and euthanasia.

## Material and Methods

This systematic review is reported according to the PRISMA guidelines [19] whenever possible with the exception of: 1) the protocol registration (item 5), 2) the ‘risk of bias’ assessment (item 12, 19 and 22) and summary measures (risk ratio, difference in means, item 13) which were not applicable for this review.

### Identification of relevant studies

The tables of contents of the ten highest-ranked journals in each of the ISI Web of Knowledge Subject Categories ‘Anesthesiology’, ‘Critical Care Medicine’ and ‘Respiratory System’ were systematically searched for relevant articles. Ranks were determined based on the impact factor of the respective journal as published in the 2012 Journal Citation Reports Science Edition, accessed via ISI Web of Knowledge [20]. Articles published between 1^st^ January 2012 and 31^st^ December 2012 were considered for evaluation without language restriction. If a journal had been listed in more than one of the categories, the following ranked journal according to the impact factor was substituted for it in one of the respective categories. Detailed information regarding selected journals is provided in Table 1 and S1 File.

**Table 1:** Identified Articles.

Category	Journal Title	Impact Factor	Included Articles	ANE	EUTH	ESM	MethodSection	Animial Care Approval#
**Anesthesiology**	Pain	5.644	63 (25.6)	52 (81.3)	63 (100.0)	accepted	no word limit	statement required
	Anesthesiology	5.163	70 (28.5)	60 (85.7)	65 (92.9)	accepted	no word limit	statement required
	British Journal of Anaesthesia	4.237	22 (8.9)	18 (81.8)	20 (90.9)	accepted	no word limit, ARRIVE guidelines referenced	statement required
	Anaesthesia	3.486	3 (1.2)	3 (100.0)	2 (66.6)	accepted	no word limit, compliance with ARRIVE guidelines demanded	approval required§
	Regional Anesthesia and Pain Medicine	3.464	11 (4.5)	10 (90.9)	9 (81.8)	accepted	no word limit	statement required
	Anesthesia and Analgesia	3.300	43 (17.5)	36 (83.7)	37 (86.0)	accepted	no word limit	statement required
	European Journal of Pain	3.067	30 (12.2)	30 (100)	22 (73.3)	accepted	no word limit	statement required
	Minerva Anestesiologica	2.818	0 (0.0)	0 (0.0)	0 (0.0)	not mentioned in authors’ guidelines	no word limit	statement required
	European Journal of Anaesthesiology	2.792	4 (1.6)	4 (100)	3 (75.0)	accepted	no word limit, ARRIVE guidelines referenced	statement required
	Pain Practice	2.605	0 (0.0)	0 (0.0)	0 (0.0)	not mentioned in authors’ guidelines	only reviews	statement required
	**Total**		246 (29.1)	213 (86.2)	222 (89.9)			
**Critical Care Medicine**	American Journal of Respiratory and Critical Care Medicine	11.041	32 (11.8)	16 (50)	32 (100)	accepted	methods limited to 500 words	approval required
	Critical Care Medicine	6.124	57 (21,0)	48 (84.2)	57 (100.0)	accepted	no word limit	statement required
	Chest	5.854	4 (1.5)	2 (50.0)	3 (75.0)	accepted, but authors are charged 150 USD;	no word limit	approval§
	Intensive Care Medicine	5.258	16 (5.8)	14 (93.3)	15 (100)	accepted	no word limit	statement required
	Critical Care	4.718	15 (5.5)	13 (81.3)	16 (100.0)	accepted	no word limit	not explicitly required
	Journal of Neurotrauma	4.295	131 (48.1)	118 (90.0)	118 (90.0)	not mentioned in authors’ guidelines	no word limit	statement required
	Resuscitation	4.104	18 (6.6)	16 (88.8)	17 (94.4)	accepted	no word limit,appended ARRIVE checklist demanded	Review Board approval identifier required
	Neurocritical Care	3.038	0 (0.0)	0 (0.0)	0 (0.0)	not mentioned in authors’ guidelines	no word limit	not explicitly required
	Current Opinion in Critical Care	2.967	0 (0.0)	0 (0.0)	0 (0.0)	accepted	only review articles	not applicable
	Seminars in Respiratory and Critical Care Medicine	2.752	0 (0.0)	0 (0.0)	0 (0.0)	not mentioned in authors’ guidelines	only review articles	not applicable
	**Total** *		273 (32.3)	227 (83.5)	258 (94.9)			
**Respiratory System**	Thorax	8.376	11 (3.4)	6 (54.5)	11 (100)	accepted	no word limit	not explicitly required
	European Respiratory Journal	5.854	16 (4.9)	10 (62.5)	16 (100)	accepted;	no word limit	not explicitly required
	Journal of Heart and Lung Transplantation	5.112	14 (4.3)	9 (64.3)	14 (100.0)	not mentioned in authors’ guidelines	no word limit	statement confirming adherence to Helsinki declaration required
	Journal of Thoracic Oncology	4.473	4 (1.2)	0 (0.0)	4 (100.0)	accepted	no word limit	not explicitly required
	American Journal of Respiratory Cell and Molecular Biology	4.148	113 (34.6)	44 (39.3)	113 (100)	accepted	limited to 500 words	not explicitly required
	Respiratory Research	3.642	27 (8.3)	15 (55.5)	27 (100.0)	accepted	no word limit	not explicitly required
	Journal of Thoracic and Cardiovascular Surgery	3.526	9 (2.8)	5 (55.5)	9 (100)	accepted	no word limit	statement required
	American Journal of Physiology—Lung Cellular and Molecular Physiology	3.523	115 (35.3)	58 (50.4)	115 (100.0)	accepted	no word limit, ARRIVE guidelines referenced	statement required
	Annals of Thoracic Surgery	3.454	7 (2.1)	2 (28.6)	7 (100)	not mentioned in authors’ guidelines	no word limit	statement required
	Lung Cancer	3.392	10 (3.1)	2 (20.0)	10 (100)	accepted	no word limit	statement required
	**Total** *		326 (38.6)	151 (46.3)	326 (100.0)			
	**Overall**		**845 (100.0)**	**591 (69.9)**	**806 (95.4)**			

Summary on the number of articles and journal characteristics according to the instructions to authors included in the final analysis with respect to 2012 ISI Web of Knowledge subject category, journal and impact factor. The journals "Seminars of Respiratory and Critical Care", "Thorax" and “Journal of Heart and Lung Transplant" were substituted for "Minerva Anestesiologica", "American Journal of Respiratory and Critical Care Medicine" and "Chest" respectively, which were listed in more than one subject category. The substitution resulted in the inclusion of "Annals of Thoracic Surgery" and "Lung Cancer" in the present review. Values are given as number (% per category).

*: percentage related to total number of articles (845);

^#^: animal care approval statement in methods section according to the instructions to authors,

^§^: animal care approval required, but no explicit statement regarding animals care approval in methods section in the instructions to authors listed.

ANE: number of manuscripts involving animal anesthesia (percentages given related to number of included articles); EUTH: number of manuscripts involving animal euthanasia (percentages given related to number of included articles); ESM: Availability of an electronic online supplement.

### Eligibility criteria

Inclusion criteria with respect to the PICOS criteria [21] were as follows: 1) population: small laboratory mammals are defined as members of the clade glires (rodents and lagomorpha), also including animals used for cell and tissue isolation, 2) intervention: any form of anesthesia and / or euthanasia, 3) comparison: not specified, 4) outcome: defined by primary and secondary endpoints, 5) study: experimental studies presented in original research articles. Primary endpoints were defined as follows: 1) type of anesthesia and anesthetic agents (dosage, route of administration, form of application), 2) type of euthanasia (methods, anesthetic agents [dosage, route of administration, form of application]). Secondary endpoints of this review included animal characteristics, monitoring, mechanical ventilation, fluid management, postoperative pain therapy, animal care approval, sample size calculation and performed interventions.

Manuscripts were excluded, if only purchased cells were used in the reported experiments or the publication was not an original research article.

### Manuscript selection and data abstraction

The articles included in this review were selected by examining titles, abstracts and the full text if a potentially relevant manuscript was identified. Two authors (CU, HK) abstracted the data on the a priori defined inclusion criteria with respect to anesthesia, euthanasia and monitoring procedures as well as animal and manuscript characteristics independently and in duplicate. A posteriori, the reporting of animal characteristics, anesthesia, euthanasia and monitoring was characterized for the three categories ‘Anesthesiology’, ‘Critical Care Medicine’ and ‘Respiratory System’, impact factor (higher and lower as five) as well as for journals advising authors to describe anesthesia in their manuscript instructions separately. Quality of reporting was assessed as completeness of data on animal characteristics, anesthesia, euthanasia, ethical statements as well as details of the study planning as described in the ARRIVE guidelines [18]. Frequency of reporting describes how often a specific intervention or characteristic was stated in the analyzed data. Research protocols using several different anesthesia and/or euthanasia methods within one manuscript were counted separately. An outcome was classified as 'not specified' if a range for the respective value or a general term (e.g. 'euthanized', 'sacrificed' or 'anesthetized') was used without further description. Initial survival was defined as ‘recovering from anesthesia after an intervention’. Overall survival was classified as ‘no reported euthanasia and no results or procedures reported which are contradictory to life’, such as histology or other tissue processing results of vital organs. A painful intervention was defined as a procedure involving organ injury, surgery or tissue stimulation. Premedication was classified as ‘the administration of a drug for sedation with or without combination with analgesic agents before the induction of anesthesia’. Drugs for anesthesia maintenance were used during the experimental interventions with or without premedication. The term ‘form of application’ of an agent was defined as ‘bolus’ if a single or repetitive injection was performed or as ‘continuous’ which represents a constant or varying infusion rate. The purpose of monitoring is reported in four categories: experimental procedure, anesthesia only, both or not specified. Article supplements were searched whenever available.

### Data synthesis

All analyses were performed using IBM SPSS Statistics (Vers. 20, IBM Deutschland GmbH, Ehningen, Germany). Values are given as total number and percentage of total unless specified otherwise. Animal characteristics were analyzed per publication. Anesthesia, euthanasia, as well as monitoring and mechanical ventilation were analyzed per performed intervention in each article. Percentages of outcomes were calculated with respect to the reported outcome (excluding 'not reported' and 'not specified'). Frequency of reporting among the journal categories and between journals with impact factor equal or greater than five and lower than five were analyzed with a chi-square or Fisher's exact test, if appropriate. Statistical significance was accepted at p<0.05.

## Results

### Trial identification and characteristics

**Fig 1:**
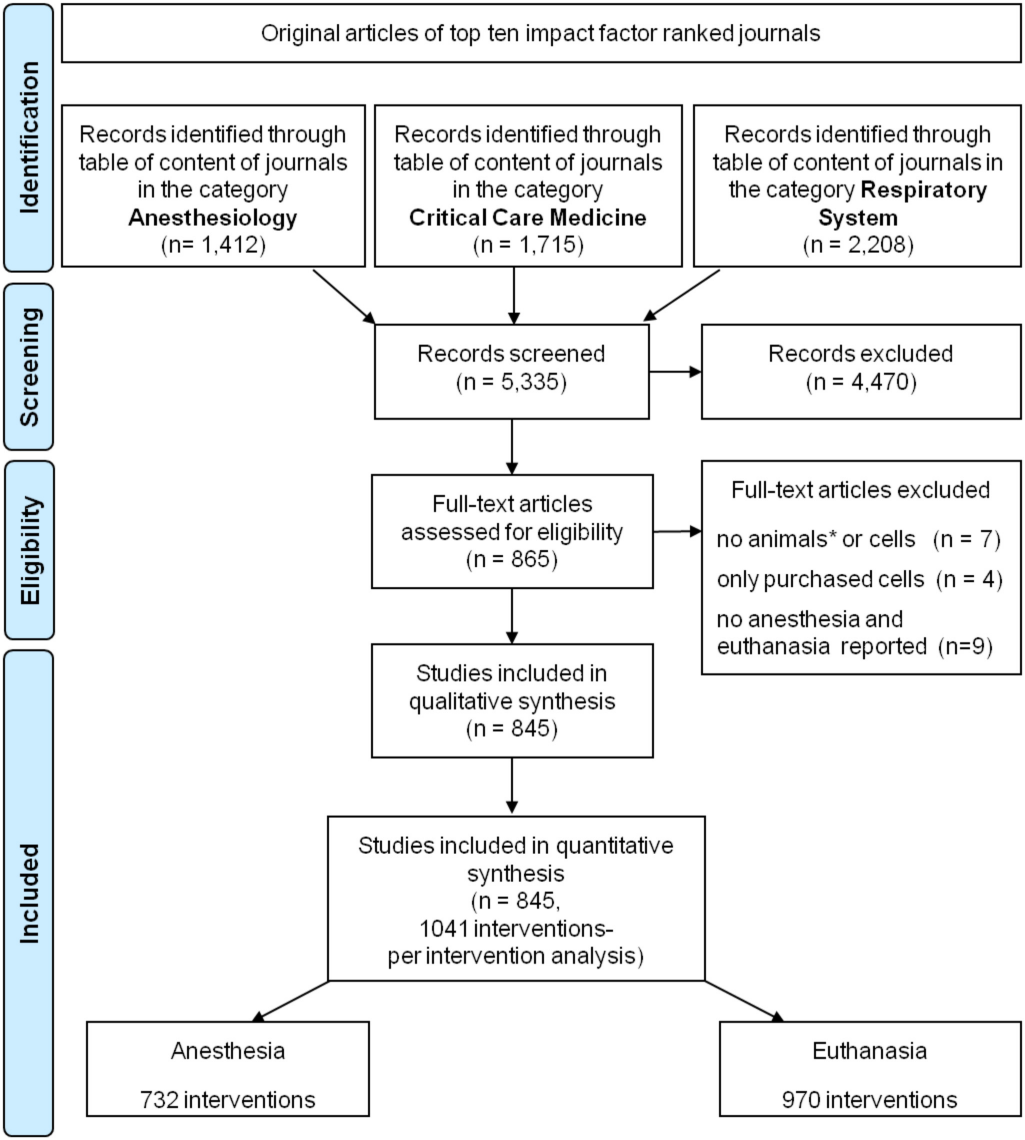
Flowchart. Studies enrolled for this systematic review. *: small laboratory animals (clade glires).

The search yielded 5,335 experimental studies in total including 865 manuscripts describing experimental studies in small laboratory mammals. Seven of these 865 manuscripts screened in full text were excluded because neither small laboratory mammals nor cells were. Four more articles were excluded because cells were not primarily isolated, but commercially purchased. Nine papers involving rodents did not report anesthesia or euthanasia. Those nine manuscripts were excluded, too. Finally, 845 articles were included in this review. Terminal experiments were conducted in 806 (95.4%) articles. 1,041 different interventions involving anesthesia and/or euthanasia of small animals were performed. Initial survival of the intervention was reported in 455/1,041 (43.7%) of the performed interventions (Table 2). Anesthesia was reported in 599/845 (70.8%) of manuscripts and performed in 732/1,041 (70.3%) of the interventions. Euthanasia was applied in 970/1,041 (93.1%) of the interventions including interventions with initial survival. The flowchart of the selection process is depicted in Fig 1.

**Table 2:** Interventions and survival.

Intervention	Species				Initial Survival		Overall Survival		Number of
	Mouse	Rat	Guinea pig	Rabbit	Yes	No	Yes	No	Interventions*
**Cell/organ harvest**	111 (53.4)	83 (40.4)	7 (3.4)	6 (2.9)	19 (9.2)	188 (90.8)	0 (0.0)	207 (100.0)	207 (19.9)
**Mechanical ventilation**	23 (46.0)	21 (42.0)	0 (0.0)	6 (12.0)	3 (6.0)	47 (94.0)	0 (0.0)	50 (100.0)	50 (4.8.)
**Organ injury/surgical procedure/TS**	255 (41.5)	343 (56.4)	0 (0.0)	12 (2.1)	364 (59.7)	246 (40.3)	50 (9.2)	560 (91.8)	610 (58.6)
**Drug administration**	25 (27.7)	63 (67.0)	2 (2.1)	3 (3.2)	39 (41.9)	54 (58.1)	16 (17.2)	77 (82.8)	93 (8.9)
**Measurement**	41 (50.0)	37 (46.3)	1 (1.2)	2 (2.4)	30 (37.0)	51 (63.0)	4 (4.9)	77 (95.1)	81 (7.8)
**Total**	455 (43.7)	547 (52.5)	10 (1.0)	29 (2.8)	455 (43.7)	586 (56.3)	70 (6.7)	971 (93.3)	1041 (100.0)

Values are given as number (%). Initial survival was defined as ‘recovering from anesthesia after an intervention’. Overall survival was defined as no reported euthanasia.

*: the number of interventions represent the denominator to all fractions; TS: tissue stimulation.

### Quality of reporting

Of the 845 analyzed papers, 129/845 (15.3%) did not include the ethical statement on animal care approval from their institution (Table A in S2 File) and 837/845 (99.1%) did not include sample size calculations, both recommended in the ARRIVE guidelines [18]. None of the 845 analyzed papers reported on all items of the ARRIVE guidelines [18].

### Frequency of reporting

#### Animal characteristics

**Fig 2:**
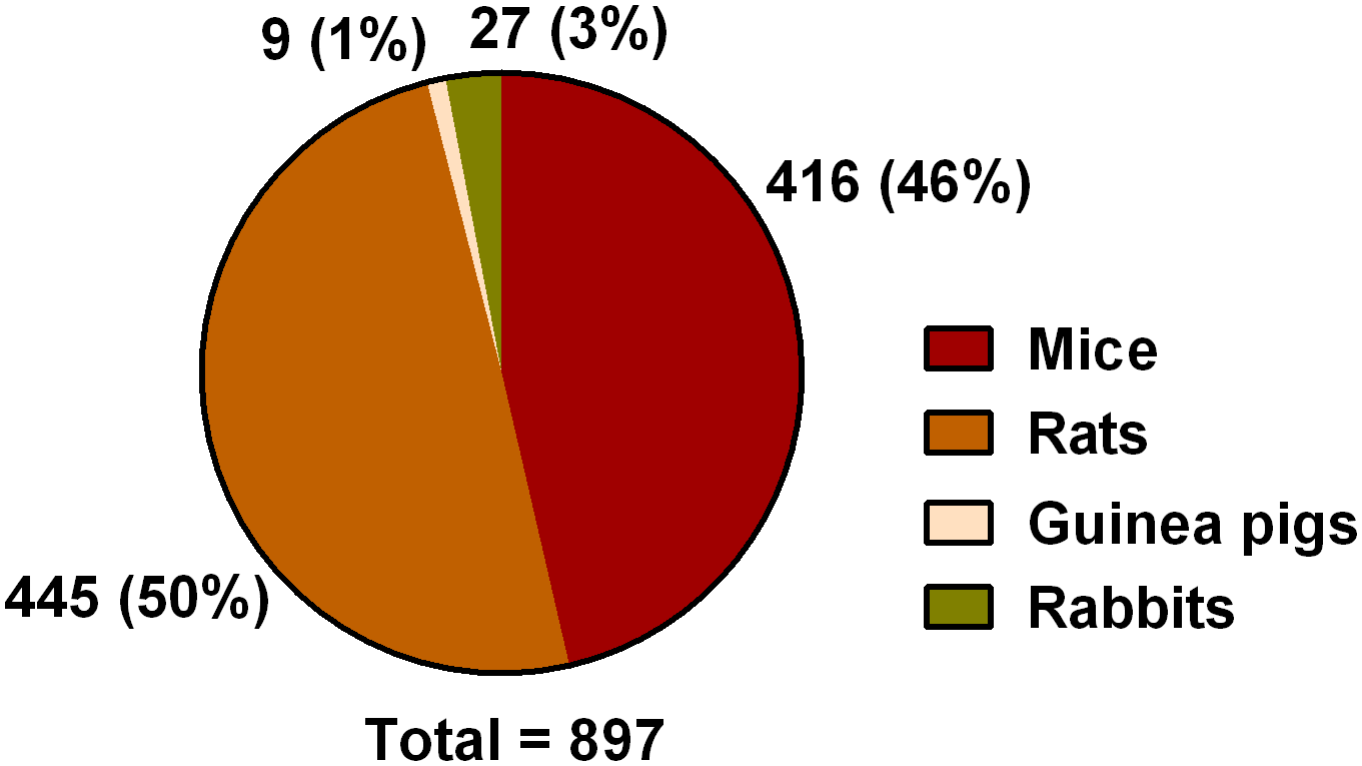
Frequencies of reported species. Values are given as number; percentage relative to total (below pie diagram). Analysis was performed per publication. The total number of 897 is explained by use of multiple species among manuscripts.

Animal characteristics are described in Table B in S2 File and Fig 2. Animal species and breeding strains are summarized in Table 3. Frequency of reporting, with respect to journal category and impact factor, is shown in Table 4. The total number of small laboratory mammals used was not reported in 533/897 (59.4%) and not specified in 15/897 (1.7%) data sets out of 845 analyzed manuscripts. The difference between data sets and analyzed manuscripts results from the possible use of multiple species in a single manuscript. A total number of 23,009 small laboratory mammals, including 6,570/23,009 (28.5%) mice, 15,729/23,009 (68.4%) rats, 40/23,009 (0.2%) guinea pigs and 670/23,009 (2.9%) rabbits was described in the remaining 349/897 (38.9%) interventions involving different small laboratory species.

**Table 3:** Animal species and breeding strains.

Species	Mouse	No. (%)	Rat	No. (%)	Guinea pig	No. (%)	Rabbit	No. (%)
**Breeding strains**	C57BL/6	260 (59.5)	Spargue Dawley	297 (66.4)	Hartley	4 (44.4)	New Zealand White	23 (85.2)
	BALB/c	58 (13.3)	Wistar	98 (21.9)			Japanese White	2 (7.4)
	129Sv	11 (2.5)	Long Evans	11 (2.5)				
	Swiss	6 (1.4)	Lewis	10 (2.2)				
	C3H/HeN	5 (1.1)	Brown Norway	5 (1.1)				
	Swiss Webster	4 (0.9)	Fisher 344	3 (0.7)				
	other*	41 (9.4)	other*	15 (3.4)				
	n/r	52 (11.9)	n/r	8 (1.8)	n/r	5 (55.6)	n/r	2 (7.4)
	total	437 (100.0)	total	447 (100.0)	total	9 (100.0)	total	27 (100.0)

Values are given as absolute number and percentage No.(%). The total number of species and breeding strains is varying from 897, since animals were counted per strain separately. n/r: not reported;

*: race frequency < 0.5%.

**Table 4:** Frequency of reporting with respect to journal categories and impact factor.

	Category				Impact Factor		
	ANE	CCM	RS	P value	< 5	≥ 5	P value
**Animal characteristics**							
No reported number of animals	115/533 (21.6%)	119/533 (22.3%)	299/533 (56.1%)	P<0.001	376/533 (70.5%)	157/533 (29.4%)	P<0.001
No reported beeding strains	16/67 (23.8%)	15/67 (22.4%)	36/67 (53.7%)	P = 0.017	46/67 (68.7%)	21/67 (31.3%)	P = 0.511
No reported sex	37/246 (15.0%)	58/246 (23.6%)	151/246 (61.4%)	P<0.001	183/246 (74.0%)	64/246 (26.0%)	P<0.001
No reported age	128/391 (32.7%)	123/391 (31.5%)	140/391 (35.8%)	P = 0.086	269/391 (68.8%)	122/391 (31.2%)	P = 0.023
No reported weight	67/436 (15.4%)	97/436 (22.2%)	272/436 (62.4%)	P<0.001	312/436 (71.6%)	124/436 (28.4%)	P<0.001
**Anesthesia**							
Reported anesthesia*	285/690 (41.3%)	257/690 (37.2%)	148/690 (21.5%)	P<0.001	423/690 (61.3%)	267/690 (38.7%)	P<0.001
No reported anesthesia^§^	54/351 (15.4%)	64/351 (18.2%)	233/351 (66.4%)	P<0.001	114/351 (32.5%)	237/351 (67.5%)	P<0.001
Premedication	76/169 (45.0%)	71/169 (42.0%)	22/169 (13.0%)	P = 0.009	101/169 (59.8%)	68/169 (48.2%)	P = 0.636
Analgesics for premedication	3/32 (9.4%)	14/32 (43.7%)	15/32 (46.9%)	P<0.001	15/32 (46.9%)	17/32 (53.1%)	P = 0.006
Analgesics during anesthesia	54/185 (29.2%)	71/185 (38.4%)	60/185 (32.4%)	P<0.001	118/185 (63.8%)	67/185 (36.2%)	P = 0.418
Post-interventional analgesia	7/38 (18.4%)	27/38 (71.1%)	4/38 (10.5%)	P<0.001	9/38 (23.7%)	29/38 (76.3%)	P<0.001
Neuromuscular blocking agents	7/38 (18.4%)	18/38 (47.4%)	13/38 (34.2%)	P = 0.010	16/38 (42.1%)	22/38 (57.9%)	P = 0.016
Fluid therapy	22/66 (33.3%)	37/66 (56.1%)	7/66 (10.6%)	P = 0.002	41/66 (62.1%)	25/66 (37.9%)	P = 1.000
**Euthanasia**							
Reported euthanasia*	177/481 (36.8%)	183/481 (38.0%)	121/481 (25.2%)	P<0.001	324/481 (67.4%)	157/481 (32.6%)	P = 0.039
No reported euthanasia in interventions without survival	93/450 (20.7%)	112/450 (24.9%)	245/450 (54.4%)	P<0.001	274/450 (68.9%)	176/450 (39.1%)	P = 0.039
Anesthesia for euthanasia#	124/348 (35.6%)	136/348 (39.1%)	88/348 (25.3%)	P = 0.828	245/348 (70.4%)	103/348 (25.6%)	P = 0.023
Transcardial perfusion	9/37 (24.3%)	18/37 (48.7%)	10/37 (27.0%)	P = 0.231	12/37 (32.4%)	25/37 (67.6%)	P<0.001
Carbon dioxide	11/26 (42.3%)	7/26 (26.9%)	8/26 (30.8%)	P = 0.481	16/26 (61.5%)	10/26 (38.5%)	P = 0.524
Decapitation	14/22 (63.6%)	5/22 (22.7%)	3/22 (13.6%)	P = 0.028	13/22 (59.1%)	9/22 (40.9%)	P = 0.485
Exsanguination	6/23 (26.1%)	10/23 (43.5%)	7/23 (30.4%)	P = 0.547	11/23 (47.8%)	12/23 (52.2%)	P = 0.065
Other methods	13/25 (52.0%)	7/25 (28.0%)	5/25 (20.0%)	P = 0.268	14/25 (56.0%)	11/25 (44.0%)	P = 0.272
**Monitoring**							
No reported monitoring	96/439 (21.9%)	156/439 (35.5%)	187/439 (42.6%)	P<0.001	248/439 (56.5%)	191/439 (43.5%)	P = 0.130
Total reported monitoring	100/293 (34.1%)	137/293 (46.8%)	56/293 (19.1%)	P<0.001	182/293 (62.1%)	111/293 (37.9%)	P = 0.130
Heart rate (ECG or pulse plethysmography)	13/40 (32.5%)	19/40 (47.5%)	8/40 (20.0%)	P = 0.1894	26/40 (65.0%)	14/40 (35%)	P = 0.509
Blood pressure (invasive or non-invasive)	53/129 (41.1%)	57/129 (44.2%)	19/129 (14.7%)	P<0.001	57/129 (44.2%)	72/129 (55.8%)	P<0.001
Oxygenation (pulse plethysmography or BGA)	33/100 (33.0%)	49/100 (49.0%)	18/100 (18.0%)	P = 0.002	46/100 (46.0%)	54/100 (54.0%)	P = 0.005
Temperature	77/198 (38.9%)	100/198 (50.5%)	21/198 (10.6%)	P<0.001	79/198 (39.9%)	119/198 (60.1%)	P<0.001

Values are given as number in relation to total and the respective percentage. Statistical analysis was performed using a chi-square or Fisher's exact test, if appropriate. Statistical significance was accepted at α = 0.05.

*: Numbers without 'not specified' interventions,

^§^: Numbers with included 'not specified' interventions,

^#^: anesthesia for euthanasia includes combination with other methods, ANE: Anesthesiology, CCM: Critical Care Medicine, RS: Respiratory System, ECG: electrocardiography, BGA: blood gas analysis

#### Anesthesia

**Fig 3:**
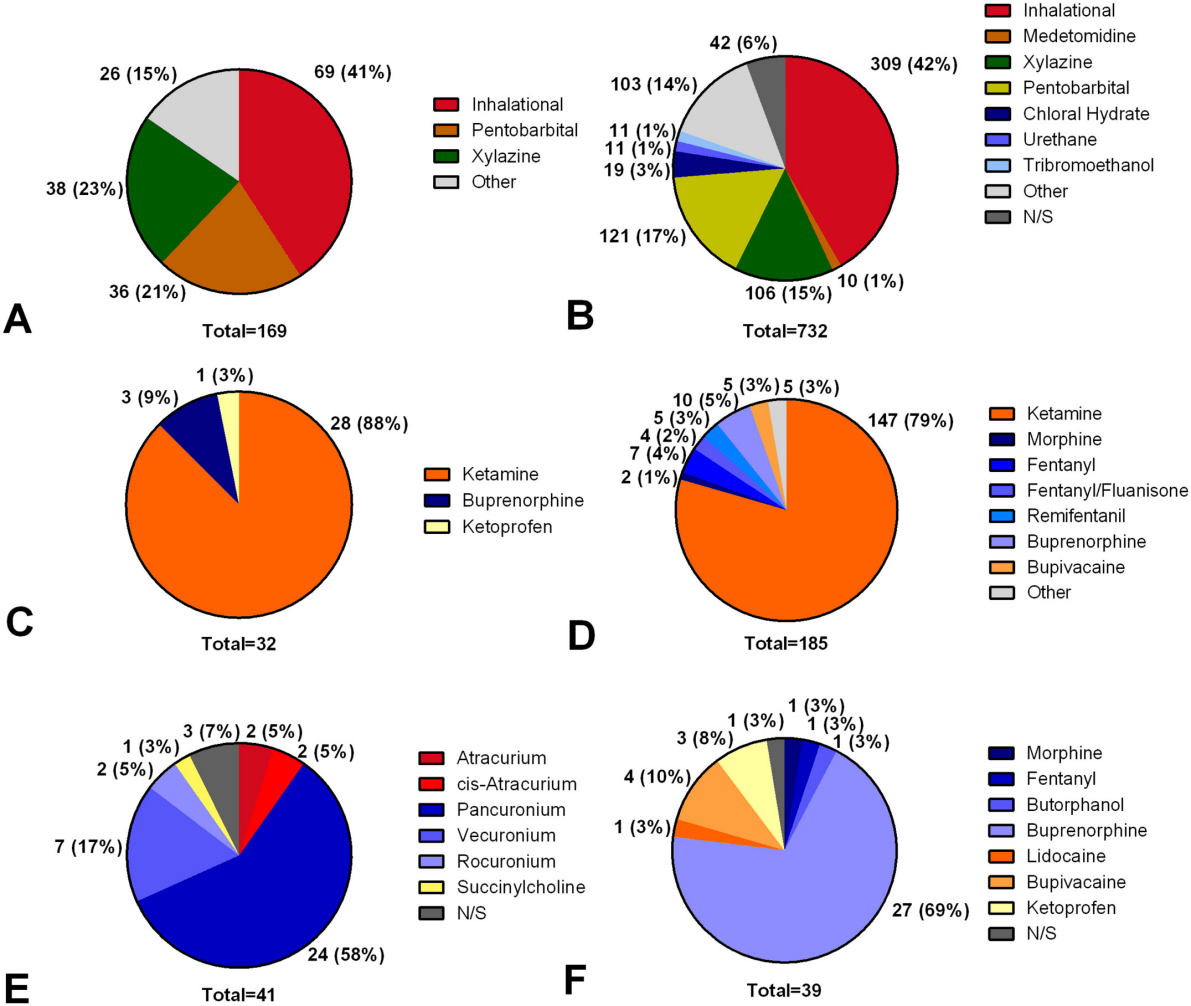
Frequencies of anesthetic drugs. Values are given as numbers and percentages relative to the total number (below pie diagram). A: Sedative drugs for premedication. Inhalational anesthetics include: isoflurane 56 (33%) and sevoflurane 13 (8%). B: Sedative agents for anesthesia maintenance. Inhalational anesthetics include: isoflurane 225 (31%), isoflurane/nitrous oxide 36 (5%), sevoflurane 28 (4%) and halothane 20 (3%). C: Analgesic drugs for premedication. D: Analgesic drugs for anesthesia maintenance. E: Neuromuscular blocking agents. F: Analgesics for postoperative pain therapy. Other: drugs with <1% occurrence. n/s: not specified.

Detailed information regarding anesthesia is summarized in Table 4, Table C in S2 File and Fig 3. Table 5 shows the dosages of the most commonly used anesthetics. Premedication was used in 169/732 (23.1%) of all interventions involving anesthesia. The use of analgesics, in addition to sedatives, for premedication was described in 32/169 premedications (18.9%). Anesthesia was not specified in 42/732 (5.7% interventions involving anesthesia). In detail, 185/690 interventions (26.8% of procedures specifying anesthesia) reported the use of analgesics in addition to sedatives for anesthesia in 117/690 (16.9% with respect to interventions involving anesthesia, and 117/610 (19.1%) with respect to painful interventions) surgical or organ injury procedures and 68/690 (9.9%) non-injurious interventions. In 12/185 (6.4%) of these remaining interventions, animals received post-operative pain therapy after procedures associated with surgery or organ injury. Regardless of intra-operative management, post-operative pain therapy was reported in 38/364 (10.4%) interventions in which animals underwent surgical procedures or organ injury with initial survival. The use of neuromuscular blocking agents was reported in 38/732 (5.2%) of the papers that described anesthetic procedures. Fluid therapy was described in 70/732 (9.6%) interventions involving anesthesia, using saline (44/70, 62.9%), lactated ringer solution (20/70, 28.6%), hydroxylethylstarch 6% (1/70, 1.4%) or dextrose 5% (1/70, 1.4%). In 4/70 interventions (5.7%), drugs for fluid therapy were not specified.

**Table 5:** Dosages of anesthetic drugs.

			Premedication				Anesthesia Maintenance			
		Unit	Mouse	Rat	Gp	Rabbit	Mouse	Rat	Gp	Rabbit
**Sedatives**	Isoflurane	[Vol%]	3.9±0.9	3.9±1.0	n/a	n/a	2.4±1.2	2.3±1.0	n/a	1.75±0
	Pentobarbital	[mg/kg BW]	53.3±20.8	52.1±11.0	n/a	n/a	70±44	44.6±14.9 [22.6±19.7]	n/a	[25.0±3.5]
	Xylazine	[mg/kg BW]	10.8±5.2	9±1	7.3±6.7	(4.5±4.5)	8.9±4.2	11.0±9.0 [20.0±14.1]	n/a	[3.25±2.1]
	Sevoflurane	[Vol%]	4±0	5.5±2.1	n/a	n/a	2.6±0.6	2.8±1.0	n/a	6.0±0.0
	Isoflurane/N_2_O	[Vol%]	n/a	n/a	n/a	n/a	1.8±0.9/ 56.6±21.7	2.3±0.8/55.8±15.7	n/a	n/a
	Halothane	[Vol%]	n/a	n/a	n/a	n/a	1.8±0.3	2.4±0.9	n/a	1.5±0.0
	Chloral hydrate	[mg/kg BW]	n/a	n/a	n/a	n/a	460.0±56.6	251.5±143.4	n/a	n/a
	Urethane	[mg/kg BW]	n/a	n/a	n/a	n/a	n/a	1300±230	1500±0.0	n/a
	Tribromo-ethanol	[mg/kg BW]	n/a	n/a	n/a	n/a	324.4±192.5	250±0	n/a	n/a
	Metedomidine	[mg/kg BW]	n/a	n/a	n/a	n/a	1.0±0.0	0.58±0.23	n/a	(0.25±0)
**Analgesics**	Ketamine	[mg/kg BW]	83.6±24.6 (60±0)	71.4±27.3 (85.0±21.2)	n/a	21.8±13.7	95.0±41.3	74.8±23.0[31.3±16.5] (50.0±17.6)	n/a	[35.0±14.4]
	Buprenorphine	[mg/kg BW]	n/a	0.025±0.007*	n/a	n/a	n/a	0.045±0.01*	n/a	n/a
	Fentanyl	[mg/kg BW]	n/a	n/a	n/a	n/a	n/a	n/a	n/a	n/a
	Remifentanil	[mg/kg BW]	n/a	n/a	n/a	n/a	n/a	[42.0±78.4]	n/a	n/a
	Bupivacaine	[mg/kg BW]	n/a	n/a	n/a	n/a	n/a	n/a	n/a	n/a
	Fentanyl/Fluanisone	[mg/kg BW]	n/a	n/a	n/a	n/a	n/a	n/a	n/a	n/a
	Morphine	[mg/kg BW]	n/a	n/a	n/a	n/a	n/a	1.8±1.0*	n/a	n/a
	Ketoprofen	[mg/kg BW]	n/a	5±0	n/a	n/a	n/a	n/a	n/a	n/a

Values are given as mean ± standard deviation and as intraperitoneal, (intramuscular) and [intravenous (mg/kg/h)] dosage. Anesthetic agents are ordered by frequency of utilization.

*: subcutaneous administration, Gp: Guinea pig.

Among journals advising or referring to advising guidelines in their instructions to authors (British Journal of Anaesthesia, Anesthesiology, Journals of the American Thoracic Society and the American Journal of Physiology—Lung Cellular and Molecular Physiology) *vs*. journals without this advise, the total number and percentage of reported anesthetic methods were as follows: reporting of anesthesia (without 'not specified'): 233/690 (33.8%) vs. 457/690 (66.2%), premedication: 42/169 (24.9%) vs. 127/169 (75.1%), analgesics for premedication: 10/32 (31.2%) vs. 22/32 (68.8%), analgesic agents during anesthesia: 73/185 (39.5%) vs. 112/185 (60.5%), post-interventional analgesia: 5/38 (12.2%) vs. 33/38 (86.8%), neuromuscular blocking agents during anesthesia: 15/38 (39.5%) vs. 23/38 (60.5%), fluid therapy: 21/66 (31.8%) vs. 45/66 (68.2%).

#### Side effects of anesthesia and unexpected death

Side effects of anesthesia such as hypothermia, hypotension, anaphylaxis or unexpected death caused by anesthetics were not reported in the retrieved manuscripts. Unexpected death was described in eight publications (0.9% of all 845 analyzed publications). Reasons for unexpected death were experimental procedure in 6/845 (0.7%) and not further specified in 2/845 manuscripts (0.2%).

#### Euthanasia

**Fig 4:**
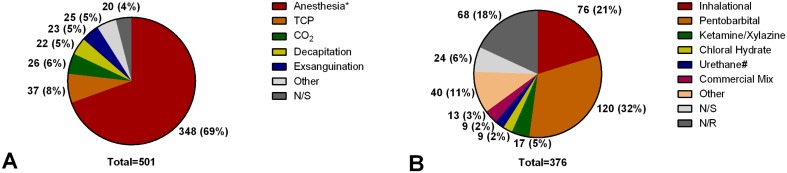
Frequencies of euthanasia. Values are given as numbers and percentages relative to the total number (below pie diagram). A: Type of Euthanasia, Anesthesia*: anesthesia overdose 121 (25%), anesthesia overdose + transcardial perfusion 135 (28%), anesthesia overdose + decapitation 38 (8%), anesthesia overdose + removal of vital organs 17 (4%), anesthesia overdose + exsanguination 37 (8%), number and percentage relative to total, respectively. B: Euthanasia drugs. Inhalational: isoflurane 47 (13%), carbon dioxide 29 (8%), number and percentage relative to total, respectively. #: urethane is not recommended as sole method for euthanasia of small laboratory mammals by the guidelines of the American Veterinary Medical Association (AVMA) guidelines for the euthanasia of animals [22] due to its slow onset of action. Other: drugs with <2% occurrence. n/s: not specified.

Type and drugs used for euthanasia are shown in Fig 4. Frequency of reporting, with respect to journal category and impact factor, is shown in Table 4. Of 971 interventions (93.2% of all interventions) without survival, euthanasia was not reported in 450/971 (46.3%) and not specified in 20/971 (2.1%), respectively. Anesthetic agents were used alone in 121/501 (24.2%) of interventions with reported euthanasia method or in combination with other euthanasia methods in 227/501 (45.3%) of interventions with reported euthanasia method. Dosage of anesthetic agents was not reported in 153/348 (43.9%) and not specified in 68/348 (19.5%) of these interventions involving euthanasia, respectively. The dosages of the three most frequently used drugs were reported as follows (values given as mean ± standard deviation): 1) pentobarbital: 135.3±115.6 mg/kg (mice, intraperitoneal), 102.6±53.8 mg/kg (rats, intraperitoneal), 150.0±40.8 mg/kg (guinea pigs, intraperitoneal), 175.0±106.1 mg/kg (rabbits, intravenous); 2) isoflurane: 3.5±1.8 vol% (mice) and 3.9±1.2 vol% (rats); 3) ketamine/xylazine: 80.0±0.0/10.0±0.0 mg/kg (rats). Route of administration was not reported in 130/348 (37.3%) interventions using anesthesia for euthanasia. Inhalative, intraperitoneal, intravenous, subcutaneous and intracardial administration was used in 92/348 (26.4%), 113/348 (32.5%), 11/348 (3.2%), 1/348 (0.3%) and 1/348 (0.3%) interventions using anesthesia for euthanasia. In 6/501 interventions with reported euthanasia (1.2%), the method used (single urethane overdose without additional euthanasia procedure) was deemed unacceptable for euthanasia in laboratory animals according to the American Veterinary Medical Association (AVMA) guidelines for the euthanasia of animals [22].

#### Monitoring and mechanical ventilation

**Fig 5:**
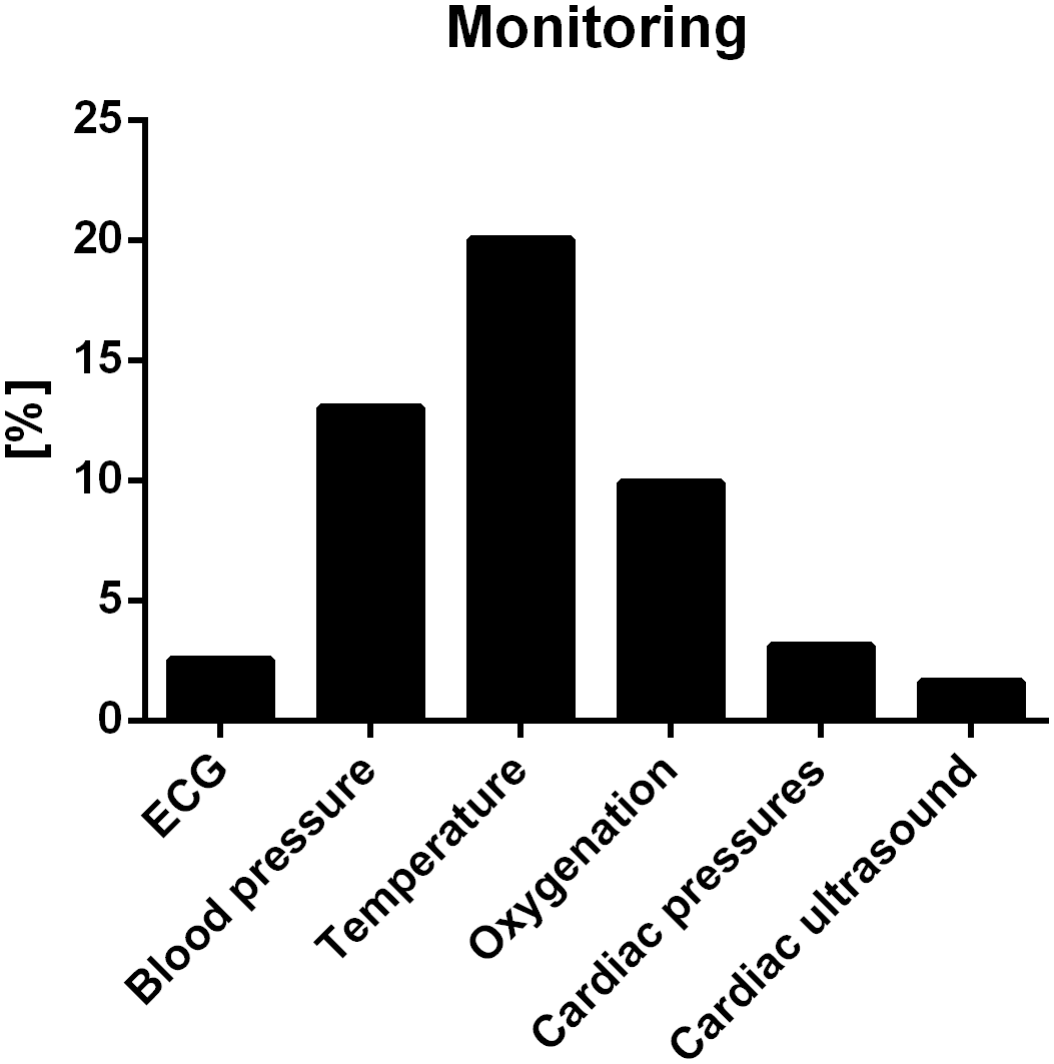
Frequencies of monitoring. Values are given as number; percentage relative to total number of interventions involving anesthesia (732/1041). ECG: electrocardiogramm, blood pressure: noninvasive or invasive, oxygenation: blood gas analysis or pulse plethysmography, cardiac pressures include ventricular or atrial pressures, pulmonary arterial pressure or central venous pressure.

No monitoring was described in 439/732 (60.0%) interventions involving anesthesia. Monitoring reported during anesthesia is depicted in Fig 5. In the remaining 293/732 (40.0%) procedures involving anesthesia, the use of monitoring techniques were described for anesthesia purposes, experimental procedure or both in 114/293 (38.9%), 26/293 (8.9%) and 113/293 (38.6%) interventions, respectively. In 40/293 interventions (13.7%), the intention of the monitoring was not specified. Venous and arterial accesses were reported in 141/845 (16.6%) and 138/845 (16.3%) of the analyzed manuscripts. Temperature was monitored in 198/732 (27.0%) interventions involving anesthesia. Detailed frequency of reporting of monitoring with respect to journal category and impact factor is shown in Table 4.

Mechanical ventilation during anesthesia was described in 179/732 (24.5%) interventions (Table 6). For monitoring during mechanical ventilation, the measurement of arterial blood pressure, blood gas analyses and spirometry was reported more frequently compared to interventions without mechanical ventilation (92, 51.4% *vs*. 61, 34.1%; 60, 33.5% *vs*. 24, 13.4% and 43, 24.0% *vs*. 19, 10.6%; values given as total number of interventions, percentage of interventions involving mechanical ventilation; mechanical ventilation vs. no mechanical ventilation). The mean tidal volume (mean ± standard deviation) used during mechanical ventilation was reported as 13.1±8.4 ml/kg (mice), 8.5±5.2 ml/kg (rats) and 7.1±2.1 ml/kg (rabbits). The mean respiratory rate (mean ± standard deviation) was reported as 155±68 per min for mice, 71±24 per min for rats, 80±0 for guinea pigs and 44±18 per min for rabbits. The fraction of inspired oxygen (mean ± standard deviation) during mechanical ventilation was described as 54 ± 35% (mice), 58 ± 33% (rats) and 68 ± 36 (rabbits). For guinea pigs no fraction of inspired oxygen and tidal volume settings were reported.

**Table 6:** Frequency of reported mechanical ventilation.

		Airway			Settings							
	MV	SGA	TS	ETT	Mode	F_I_O_2_	RR	V_T_	PEEP	ETCO_2_	I:E	Duration
reported	179 (24.5)	10 (5.6)	90 (50.3)	76 (42.5)	36 (20.1)	71 (39.7)	91 (50.8)	90 (50.3)	59 (33.0)	6 (3.4)	2 (1.1)	49 (27.4)
not reported	553 (75.5)	169 (94.4)	89 (49.7)	103 (57.5)	141 (78.8)	106 (59.2)	77 (43.0)	86 (48.0)	116 (64.8)	173 (96.6)	177 (98.9)	129 (72.1)
not specified	0 (0.0)	0 (0.0)	0 (0.0)	0 (0.0)	2 (1.1)	1 (0.6)	11 (6.1)	3 (1.7)	4 (2.2)	0 (0.0)	0 (0.0)	1 (0.6)
total	732 (100.0)	179 (100.0)	179 (100.0)	179 (100.0)	179 (100.0)	179 (100.0)	179 (100.0)	179 (100.0)	179 (100.0)	179 (100.0)	179 (100.0)	179 (100.0)
mean value								10.3±7.0ml/kg	2.8±1.8 cmH_2_O			

Values are given as number (% relative to total). MV: mechanical ventilation, SGA: supraglottic airway including supplemental oxygen inflation via nose cone and mask, TS: tracheostomy, ETT: endotracheal intubation, F_I_O_2_: fraction of inspired oxygen, RR: respiratory rate, V_T_: tidal volume, PEEP: positive endexpiratory pressure, ETCO_2_: end tidal carbon dioxide, I:E: inspiration/expiration-ratio.

#### Differences among categories and impact factor ratings

Significant differences among subject categories and impact factor ratings were found for many items covered in the ARRIVE guidelines, when comparing frequencies of reporting (Table 4). Studies reporting animal characteristics were inhomogeneously distributed among the categories, with more articles with incomplete reporting especially found in lower-impact factor journals and journals from the category 'Respiratory System'. Anesthesia was also reported inhomogeneously, with most papers reporting anesthesia published in lower-impact factor and Anesthesia journals. Inhomogeneous distribution of articles reporting euthanasia was found, too. Most articles not providing sufficient information on euthanasia were published in journals from the category 'Respiratory System' and lower-impact factor journals. Most papers not reporting monitoring come from 'Respiratory System' journals, while no inhomogeneous distribution between lower and higher impact factors were found.

## Discussion

### Main findings

Of the 845 studies included in the present review, many reported anesthesia and euthanasia procedures, while failing to provide detailed information on the performed procedures and the characteristics of the treated animals.

### Strengths and limitations

This review focuses on articles published in journals listed in the three Web of Knowledge subject categories closely related to anesthesia, so frequent use of anesthetic procedures throughout the interventions, high reporting rates and experience with small laboratory mammal anesthesia were postulated. The strength of this review lies in the comprehensive search strategy containing 30 journals and explicit inclusion and exclusion criteria. Trial selection and data abstraction were performed in duplicate.

The present systematic review has several limitations. First, the occurrence of under-reporting of monitoring, anesthesia and euthanasia practice could be a major source of bias when evaluating frequency and quality of reporting. This review focused on the quality of reporting within the manuscripts or additional files if available. Second, this systematic review is not completely reported according to the PRISMA guidelines [19], since those guidelines have been developed for clinical trials. Nevertheless, we followed these guidelines whenever possible. Third, only three categories from the 2012 Journal Citation Reports [20] have been investigated. Therefore, no conclusions about other categories can be drawn. Fourth, only a one-year period was analyzed and therefore no long-term conclusions can be drawn about developments of anesthesiological practice and quality of reporting. Fifth, tools for assessing bias like standards for clinical trials [23] are not available for experimental studies. Considering the large quantity of non-reported items revealed by this review, such tools are possibly unusable in this study. Due to the large amount of data reported incompletely, only a descriptive statistical approach was taken without performing meta-analysis. Additionally, the ARRIVE guidelines [18] were published in 2010 and the time for adaption of the guidelines in all manuscripts could have been too short. However, those guidelines summarize only general knowledge comparable to ‘good clinical practice’ for clinical trials. In our opinion, quality standards similar to clinical studies with respect to reporting and clinical practice should be established and followed in basic research employing animal models.

### Quality of reporting

Based on the findings of this review, the quality of reporting regarding the investigated outcomes is poor. As a direct consequence, it would be difficult to reproduce the results reported in many publications. None of the 845 analyzed papers reported on all items of the ARRIVE guidelines [18]. Omission of items is not a violation of the guidelines and explicitly allowed, but could make reproduction of results difficult.

### Frequency of reporting

Most articles failed to provide details on sample size calculation. Data on basic animal characteristics, especially the number of animals used as well as age and weight, was lacking in approximately 50% of the articles. Animal care approval was also described in only approximately 85% of the manuscripts. Unanticipated mortality due to anesthetic drugs or experimental procedures was rarely reported, introducing a significant survivor bias. The reporting of animal characteristics, anesthesia, euthanasia and monitoring tended to be worst in journals belonging to the category ‘Respiratory System‘. One possible explanation for this finding is that there are more manuscripts related to basic research in this category. In contrast journals with advising guidelines regarding anesthesia showed lower frequencies of reported anesthetic methods. This could be based on the fact that more manuscripts involving anesthesia were published in journals without advising guidelines. These findings are supported by a recently published study investigating the ethical quality of reporting in three journals related to critical care [17]. Cultural differences between basic research and both human clinical research with its strict rules concerning reporting of studies and human as well as veterinary clinical anesthesiology could contribute to this phenomenon. Despite of these potential differences, authors from basic research should strive for completeness of reporting on clinical procedures performed during studies, for example by considering the ARRIVE guidelines [18].

In spite of this lack of reported data, all investigated manuscripts passed a peer-review and editorial board process. Possible explanations for the lack of basic information regarding animal characteristics, animal care approval and relevant information regarding anesthesia and euthanasia are: 1) due to space constrains in the methods sections of the respective journals, the authors may not elaborate on these characteristics more extensively, especially if the opportunity of an online data supplement is not available; 2) editors and reviewers are confronted with a large amount of manuscripts, 3) the review process might be more focused on the results and their interpretation than on formal requirements of the method section.

### Ways to improve quality and frequency of reporting

To improve quality and frequency of reporting, potential space constraints could be addressed by accepting online supplementary data. Furthermore, journals could include a checkbox with a statement of institutional animal care and use committee approval in the journal submission mask, as well as a checkbox to indicate that authors have read the ARRIVE guidelines [18]. During peer review, the ARRIVE guidelines [18] could be considered to ensure that all information is provided in the manuscript to adequately characterize a given animal study. However, it cannot be in the journals’ responsibility, but rather the respective institutional animal care and use committees’, to make sure that the reported study has been performed in accordance with the approved animal protocols.

### Pain management

Detecting states of pain in small laboratory mammals requires careful behavioral assessment [24,25] and can be challenging [26–29]. Researchers and animal care personnel have to be trained to recognize painful animal behavior to prevent unnecessary suffering [30].

For possibly painful interventions, administration of analgesic drugs during maintenance of anesthesia in addition to sedative agents is frequently used in humans [31]. In the animal studies included in this review, the administration of analgesics was reported in only 19.1% of all painful interventions describing drugs for anesthesia maintenance. However, this fact should be interpreted with caution because of bias due to possible underreporting as previously mentioned. Insufficient analgesia can cause hemodynamic instability and stress responses on metabolic and hormone level, which may influence the experimental endpoints [32]. On the other hand, analgesic substances can influence animal outcomes by modulation of inflammatory pathways (non-steroidal anti-inflammatory drugs) or impair respiratory drive, requiring mechanical ventilation (opioid analgesics). As a direct consequence the specific research question and choice of sedative and/or analgesic drugs have to be balanced individually. The role of the institutional animal care and use committee is therefore crucial to evaluate the importance of the scientific needs in the context of animal welfare.

The extension of analgesic therapy into the post-operative period after painful interventions is a key element to modern human anesthetic strategies and a hallmark of humane animal care in laboratory research [33]. In animal research, post-operative pain management is important in order to minimize animal suffering and reduce post-operative stress [12]. In the present review, only in 10.4% of the interventions in which animals initially survived the respective procedure, post-operative treatment with analgesic agents was reported. In context of the published literature, intra- and post-operative analgesic therapy is rarely performed [7,14,17]. Therefore, some of the investigated journals (e.g. British Journal of Anaesthesia, Anesthesiology, Journals of the American Thoracic Society and the American Journal of Physiology- Lung Cellular and Molecular Physiology) advise researchers in their instructions for authors regarding anesthesia in laboratory animals [34–37]. The British Journal of Anaesthesia highlighted that e.g. a single intraperitoneal pentobarbital anesthesia alone is insufficient to provide analgesia. Nevertheless this strategy was frequently found in the present review. Also the use of postoperative pain therapy was recommended, whenever necessary [34].

### General considerations

Some principles of anesthesia may seem unintuitive for researchers without explicit anesthesiological background. In addition, anesthesia in small laboratory mammals varies in part from clinical practice. The main risks of anesthesia in small laboratory animals are hypotension, hypoxemia, hypoventilation and hypothermia, which need to be considered in planning and performing the experiments.

First, preanesthetic medication (sedation and analgesia), especially prior to painful interventions. reduces apprehension and stress resulting in lower intraoperative doses of anesthetic agents and reduction of side effects such as hypotension [38]. In contrast, in the present review, only few studies reported the use of sedative premedication, and only 19% of these trials described additional use of analgesics. Increased animal stress levels, resulting from a lack of premedication, may modulate inflammatory response and compromise experimental results [39].

Second, management of body temperature is essential since small laboratory mammals are at high risk for intraoperative hypothermia due to their high relative body surface compared to body weight. Physiological functions as immunological response and hemostasis are highly temperature-dependent [40,41]. Therefore, close temperature monitoring and management is crucial for experiments to be comparable and reproducible. Only in 20% of the anesthetic procedures body temperature monitoring was described.

Third, hemodynamic monitoring is an important tool for titrating depth of anesthesia and avoiding cardiovascular depression especially if the animal is paralyzed [42]. In order to obtain accurate and reproducible measurements, it is important to use devices specifically designed for monitoring and mechanical ventilation of small laboratory mammals. Specific methods based on neuro-monitoring to determine depth of anesthesia available in humans are unusable in small laboratory mammals due to technical limitations [43]. Apart from the reaction to a painful stimulus, hemodynamic monitoring is another method for assessing sufficient anesthesia [44]. In approximately 40% of the anesthetic procedures hemodynamic monitoring was reported.

Fourth, the sex of the animal can have an effect on the anesthetic dosage, due to differences in metabolism and lean body mass [45]. Furthermore, anesthetic dosage is also influenced by the age of the small laboratory mammal, which means that younger animals need higher doses than older animals in relation to their body weight [46–48].

Fifth, routine fluid therapy for hemodynamic stabilization was only described in few interventions, most commonly using saline. In contrast, balanced crystalloid solution has become standard of care in human clinical practice [49].

Sixth, the method of euthanasia should be performed according to the AVMA guidelines [22]. In the present systematic review, in six manuscripts single urethane injections for euthanasia were used, mainly with approval of an institutional animal care and use committee. Alternative euthanasia methods could have been used in the opinion of the authors. Researchers as well as members of the institutional animal care and use committees should be aware of the ethical guidelines regarding euthanasia while conducting and reviewing animal experiments.

### Implication for further studies

As for all types of medications, effects as well as side effects of anesthesia need to be considered carefully. For many anesthetic agents, common side effects as cardiorespiratory depression in rodent models are described [6]. Apart from that, there is strong evidence that anesthetic and analgesic drugs have immunomodulatory properties [50–52], which have the potential to influence outcomes in many experimental settings, e.g. joint inflammation and sepsis. Similar effects are known for carbon dioxide which was used for euthanasia in some interventions [53]. Concerns about distress and pain with carbon dioxide euthanasia are also raised [54]. However, AVMA guidelines [22] state that euthanasia with carbon dioxide is “acceptable with conditions” for laboratory rodents and other species. It is important to consider the effects of anesthesia and euthanasia in the context of the respective study protocols.

Reproducibility is an essential criterion for the validity of experimental research. The problem of insufficient reporting of data contributes to a lack of reproducibility, as seen in recent publications on cancer research [55,56]. The incomplete data provided in some manuscripts would make it difficult to reproduce the respective findings and compare them to other experiments.

Whenever study protocols allow, we believe that anesthesia management in small laboratory mammals for laboratory research should include premedication, hypnotic and analgesic agents as well as postoperative pain therapy. Basic monitoring of anesthesia should include at least heart rate and body temperature. Side effects of anesthetic agents should be considered while designing the research protocol and unexpected mortality of laboratory animals should be reported in the publication.

In the context of this review, guidelines for small laboratory mammals anesthesia based on more than expert consensus are desirable as already established for other species [57,58]. Ideally, these guidelines should be embraced by the leading societies in human and veterinary medicine. Concerning quality of reporting, both authors and reviewers should strive towards provision of all basic study information, for example by considering the ARRIVE guidelines [18].

## Conclusion

The present systematic review revealed insufficient reporting of anesthesia and euthanasia methods throughout experimental studies in small laboratory mammals. Furthermore, the present review shows that there is a high need for application of existing guidelines for reporting experimental animal research. Specific guidelines for anesthesia and euthanasia regimens should be considered and reported in future studies to guarantee comparability as well as quality of animal experiments. This is of special interest when translating experimental findings to future clinical applications.

## Supporting Information

S1 File Identified Articles. Includes all references of the manuscripts retrieved by this review.
(DOCX)

S2 File Additional Results. Contains Table A—Frequency of animal care approval, Table B—Animal characteristics and Table C—Dosage, route and application of anesthetic agents and fluid therapy.
(DOCX)

## References

[1] Gargiulo S, Greco A, Gramanzini M, Esposito S, Affuso A, Brunetti A, et al Mice anesthesia, analgesia, and care, part I: Anesthetic considerations in preclinical research. ILAR J. 2012; 53: E55–69. 10.1093/ilar.53.1.55 23382271

[2] Luan F, Cordeiro MN, Alonso N, Garcia-Mera X, Caamano O, Romero-Duran FJ, et al TOPS-MODE model of multiplexing neuroprotective effects of drugs and experimental-theoretic study of new 1,3-rasagiline derivatives potentially useful in neurodegenerative diseases. Bioorg Med Chem. 2013; 21: 1870–1879. 10.1016/j.bmc.2013.01.035 23415089

[3] Di Ianni ME, Enrique AV, Palestro PH, Gavernet L, Talevi A, Bruno-Blanch LE. Several new diverse anticonvulsant agents discovered in a virtual screening campaign aimed at novel antiepileptic drugs to treat refractory epilepsy. J Chem Inf Model. 2012; 52: 3325–3330. 10.1021/ci300423q 23181365

[4] Bouzom F, Ball K, Perdaems N, Walther B. Physiologically based pharmacokinetic (PBPK) modelling tools: How to fit with our needs? Biopharm Drug Dispos. 2012; 33: 55–71. 10.1002/bdd.1767 22228149

[5] Sasai Y. Cytosystems dynamics in self-organization of tissue architecture. Nature. 2013; 493: 318–326. 10.1038/nature11859 23325214

[6] Gruber FP, Hartung T. Alternatives to animal experimentation in basic research. ALTEX. 2004; 21 Suppl 1: 3–31. 15586255

[7] Richardson CA, Flecknell PA. Anaesthesia and post-operative analgesia following experimental surgery in laboratory rodents: Are we making progress? Altern Lab Anim. 2005; 33: 119–127. 16180987 10.1177/026119290503300207

[8] Yatabe T, Kawano T, Yamashita K, Yokoyama M. Preoperative carbohydrate-rich beverage reduces hypothermia during general anesthesia in rats. J Anesth. 2011; 25: 558–562. 10.1007/s00540-011-1170-z 21607766

[9] Boyko M, Kuts R, Gruenbaum BF, Melamed I, Gruenbaum SE, Klein M, et al The role of hypothermia in the regulation of blood glutamate levels in naive rats. J Neurosurg Anesthesiol. 2013; 25: 174–183. 10.1097/ANA.0b013e31827ee0ac 23295267

[10] Kanazawa M, Ando S, Tsuda M, Suzuki T. The effect of amino acid infusion on anesthesia-induced hypothermia in muscle atrophy model rats. J Nutr Sci Vitaminol (Tokyo). 2010; 56: 117–122. 20495293 10.3177/jnsv.56.117

[11] van den Brink I, van de Pol F, Vaneker M, Kox M, Schellekens WJ, Ritskes-Hoitinga M, et al Mechanical ventilation of mice under general anesthesia during experimental procedures. Lab Anim (NY). 2013; 42: 253–257. 23783316 10.1038/laban.252

[12] Flecknell PA. Anaesthesia of animals for biomedical research. Br J Anaesth. 1993; 71: 885–894. 8280560 10.1093/bja/71.6.885

[13] Coulter CA, Flecknell PA, Richardson CA. Reported analgesic administration to rabbits, pigs, sheep, dogs and non-human primates undergoing experimental surgical procedures. Lab Anim. 2009; 43: 232–238. 10.1258/la.2008.008021 19116294

[14] Coulter CA, Flecknell PA, Leach MC, Richardson CA. Reported analgesic administration to rabbits undergoing experimental surgical procedures. BMC Vet Res. 2011; 7: 12-6148-7-12. 10.1186/1746-6148-7-12 PMC3058034 21338514

[15] Perry P. The ethics of animal research: A UK perspective. ILAR J. 2007; 48: 42–46. 17170495 10.1093/ilar.48.1.42

[16] Festing S, Patel T. The ethics of research involving animals: A review of the nuffield council on bioethics report from a research perspective. Altern Lab Anim. 2005; 33: 654–658. 16419360 10.1177/026119290503300603

[17] Bara M, Joffe AR. The ethical dimension in published animal research in critical care: The public face of science. Crit Care. 2014; 18: R15 10.1186/cc13694 24423201 PMC4056799

[18] Kilkenny C, Browne WJ, Cuthill IC, Emerson M, Altman DG. Improving bioscience research reporting: The ARRIVE guidelines for reporting animal research. PLoS Biol. 2010; 8: e1000412 10.1371/journal.pbio.1000412 20613859 PMC2893951

[19] Moher D, Liberati A, Tetzlaff J, Altman DG, PRISMA Group. Preferred reporting items for systematic reviews and meta-analyses: The PRISMA statement. BMJ. 2009; 339: b2535 10.1136/bmj.b2535 19622551 PMC2714657

[20] 2012 Journal Citation Reports Science Edition. Available: http://admin-apps.webofknowledge.com/JCR/JCR?SID.

[21] Riva JJ, Malik KM, Burnie SJ, Endicott AR, Busse JW. What is your research question? an introduction to the PICOT format for clinicians. J Can Chiropr Assoc. 2012; 56: 167–171. 22997465 PMC3430448

[22] American Veterinary Medical Association. (2013) AVMA guidelines for the euthanasia of animals: 2013 edition. Available: https://www.avma.org/KB/Policies/Documents/euthanasia.pdf.

[23] Higgins JP, Altman DG, Gotzsche PC, Juni P, Moher D, Oxman AD, et al The cochrane collaboration's tool for assessing risk of bias in randomised trials. BMJ. 2011; 343: d5928 10.1136/bmj.d5928 22008217 PMC3196245

[24] Langford DJ, Bailey AL, Chanda ML, Clarke SE, Drummond TE, Echols S, et al Coding of facial expressions of pain in the laboratory mouse. Nat Methods. 2010; 7: 447–449. 10.1038/nmeth.1455 20453868

[25] Urban R, Scherrer G, Goulding EH, Tecott LH, Basbaum AI. Behavioral indices of ongoing pain are largely unchanged in male mice with tissue or nerve injury-induced mechanical hypersensitivity. Pain. 2011; 152: 990–1000. 10.1016/j.pain.2010.12.003 21256675 PMC3079194

[26] Hawkins P. Recognizing and assessing pain, suffering and distress in laboratory animals: A survey of current practice in the UK with recommendations. Lab Anim. 2002; 36: 378–395. 12396281 10.1258/002367702320389044

[27] Robertson SA. Pain management in laboratory animals—are we meeting the challenge? J Am Vet Med Assoc. 2002; 221: 205–208. 12118580 10.2460/javma.2002.221.205

[28] Roughan JV, Flecknell PA. Evaluation of a short duration behaviour-based post-operative pain scoring system in rats. Eur J Pain. 2003; 7: 397–406. 12935791 10.1016/S1090-3801(02)00140-4

[29] Roughan JV, Flecknell PA. Pain assessment and control in laboratory animals. Lab Anim. 2003; 37: 172 12689430 10.1258/00236770360563831

[30] Flecknell P, Gledhill J, Richardson C. Assessing animal health and welfare and recognising pain and distress. ALTEX. 2007; 24 Spec No: 82–83. 19835069

[31] Merchant R, Chartrand D, Dain S, Dobson G, Kurrek M, Lagace A, et al Guidelines to the practice of anesthesia revised edition 2013. Can J Anaesth. 2013; 60: 60–84. 10.1007/s12630-012-9820-7 23264010

[32] Huiku M, Uutela K, van Gils M, Korhonen I, Kymalainen M, Merilainen P, et al Assessment of surgical stress during general anaesthesia. Br J Anaesth. 2007; 98: 447–455. 17329347 10.1093/bja/aem004

[33] Guillen J. FELASA guidelines and recommendations. J Am Assoc Lab Anim Sci. 2012; 51: 311–321. 22776188 PMC3358979

[34] British Journal of Anaesthesia: Instructions to Authors. Available: http://www.oxfordjournals.org/our_journals/bjaint/for_authors/general.html.

[35] AJRCCM Instructionsfor Contributors. Available: http://www.atsjournals.org/page/AJRCCM/instructions_for_contributors.

[36] AJRCCM Instructionsfor Contributors. Available: http://www.atsjournals.org/page/AJRCMB/instructions_for_contributors.

[37] JAP Instructions for Authors. Available: http://www.the-aps.org/mm/Publications/Info-For-Authors/Animal-and-Human-Research.

[38] Cesarovic N, Jirkof P, Rettich A, Nicholls F, Arras M. Combining sevoflurane anesthesia with fentanyl-midazolam or s-ketamine in laboratory mice. J Am Assoc Lab Anim Sci. 2012; 51: 209–218. 22776121 PMC3314524

[39] Gorska P. Principles in laboratory animal research for experimental purposes. Med Sci Monit. 2000; 6: 171–180. 11208307

[40] Roberts NJ Jr. Temperature and host defense. Microbiol Rev. 1979; 43: 241–259. 390356 10.1128/mr.43.2.241-259.1979 PMC281473

[41] Lindenblatt N, Menger MD, Klar E, Vollmar B. Sustained hypothermia accelerates microvascular thrombus formation in mice. Am J Physiol Heart Circ Physiol. 2005; 289: H2680–7. 16100248 10.1152/ajpheart.00425.2005

[42] Shafer SL, Stanski DR. Defining depth of anesthesia. Handb Exp Pharmacol. 2008; 182:409–423. 43 Cove ME, Pinsky MR. Perioperative hemodynamic monitoring. Best Pract Res Clin Anaesthesiol. 2012; 26: 453–462. 10.1007/978-3-540-74806-9_19 23351232

[43] Cove ME, Pinsky MR. Perioperative hemodynamic monitoring. Best Pract Res Clin Anaesthesiol. 2012; 26: 453–462. 10.1016/j.bpa.2012.10.003 23351232

[44] Wagner AE, Brodbelt DC. Arterial blood pressure monitoring in anesthetized animals. J Am Vet Med Assoc. 1997; 210: 1279–1285. 9143529

[45] Zambricki EA, Dalecy LG. Rat sex differences in anesthesia. Comp Med. 2004; 54: 49–53. 15027618

[46] Bolander HG, Wahlstrom G. Age-related changes in CNS-sensitivity to hexobarbital and thiopental in the rat. Arch Int Pharmacodyn Ther. 1984; 267: 213–223. 6712356

[47] Koblin DD, Lurz FW, Eger EI. Age-dependent alterations in nitrous oxide requirement of mice. Anesthesiology. 1983; 58: 428–431. 6837994 10.1097/00000542-198305000-00006

[48] Larsson JE, Wahlstrom G. The influence of age and administration rate on the brain sensitivity to propofol in rats. Acta Anaesthesiol Scand. 1998; 42: 987–994. 9773145 10.1111/j.1399-6576.1998.tb05360.x

[49] Reinhart K, Perner A, Sprung CL, Jaeschke R, Schortgen F, Johan Groeneveld AB, et al Consensus statement of the ESICM task force on colloid volume therapy in critically ill patients. Intensive Care Med. 2012; 38: 368–383. 10.1007/s00134-012-2472-9 22323076

[50] Fuentes JM, Talamini MA, Fulton WB, Hanly EJ, Aurora AR De Maio A. General anesthesia delays the inflammatory response and increases survival for mice with endotoxic shock. Clin Vaccine Immunol. 2006; 13: 281–288. 16467339 10.1128/CVI.13.2.281-288.2006 PMC1391927

[51] Galley HF, DiMatteo MA, Webster NR. Immunomodulation by anaesthetic, sedative and analgesic agents: Does it matter? Intensive Care Med. 2000; 26: 267–274. 10823382 10.1007/s001340051149

[52] Al-Hashimi M, Scott SW, Thompson JP, Lambert DG. Opioids and immune modulation: More questions than answers. Br J Anaesth. 2013; 111: 80–88. 10.1093/bja/aet153 23794649

[53] Kos M, Kuebler JF, Jesch NK, Vieten G, Bax NM, van der Zee DC, et al Carbon dioxide differentially affects the cytokine release of macrophage subpopulations exclusively via alteration of extracellular pH. Surg Endosc. 2006; 20: 570–576. 16437285 10.1007/s00464-004-2175-6

[54] Conlee KM, Stephens ML, Rowan AN, King LA. Carbon dioxide for euthanasia: Concerns regarding pain and distress, with special reference to mice and rats. Lab Anim. 2005; 39: 137–161. 15901358 10.1258/0023677053739747

[55] Picha B, Thompson M, Vondriska TM. Preclinical trials: Keep 'reproducibility' in context. Nature. 2012; 485: 41. 10.1038/485041d 22552083

[56] Begley CG, Ellis LM. Drug development: Raise standards for preclinical cancer research. Nature. 2012; 483: 531–533. 10.1038/483531a 22460880

[57] Bednarski R, Grimm K, Harvey R, Lukasik VM, Penn WS, Sargant B, et al (2011) AAHA anesthesia guidelines for dogs and cats. J Am Anim Hosp Assoc. 2011; 47: 377–385. 10.5326/JAAHA-MS-5846 22058343

[58] Beckman B. Anesthesia and pain management for small animals. Vet Clin North Am Small Anim Pract. 2013; 43: 669–688. 10.1016/j.cvsm.2013.02.006 23643026

