# Supplementary material for: Anesthesia and Monitoring in Small Laboratory Mammals Used in Anesthesiology, Respiratory and Critical Care Research: A Systematic Review on the Current Reporting in Top-10 Impact Factor Ranked Journals
Source: PLoS One. 2015 Aug 25;10(8):e0134205. doi: 10.1371/journal.pone.0134205 (PMC4549323; doi:10.1371/journal.pone.0134205)
Supplement: S1 File — Includes all references of the manuscripts retrieved by this review. (DOCX) [file pone.0134205.s001.docx]

**Anesthesia and monitoring in small laboratory mammals used in anesthesiology, respiratory and critical care research: a systematic review on the current reporting in top-10 impact factor ranked journals**

**SUPPORTING INFORMATION File 1– Identified Articles**

Christopher Uhlig, Hannes Krause, Thea Koch, Marcelo Gama de Abreu, Peter Markus Spieth

Department of Anaesthesiology and Intensive Care Therapy, University Hospital Dresden, Technische Universität Dresden, Dresden, Germany

*C. Uhlig and H. Krause contributed equally to this paper.*

*Correspondence and reprint requests to*:

Dr. Peter Markus Spieth, Pulmonary Engineering Group, Department of Anesthesiology and Intensive Care Medicine, University Hospital Dresden, Technische Universität Dresden, Fetscherstraße 74, 01307 Dresden, Germany. Email: [peter.spieth@uniklinikum-dresden.de](mailto:mgabreu@uniklinikum-dresden.de), Tel: +4935145816006, Fax: +493514584336.

**REFERENCES OF INCLUDED MANUSCRIPTS**

**I. Category: Anesthesiology**

**1. Pain**

1. Ahmed AS, Li J, Erlandsson-Harris H, Stark A, Bakalkin G, Ahmed M: **Suppression of pain and joint destruction by inhibition of the proteasome system in experimental osteoarthritis.** Pain 2012, **153**(1):18-26.

2. Aira Z, Buesa I, Garcia del Cano G, Salgueiro M, Mendiable N, Mingo J, Aguilera L, Bilbao J, Azkue JJ: **Selective impairment of spinal mu-opioid receptor mechanism by plasticity of serotonergic facilitation mediated by 5-HT2A and 5-HT2B receptors.** Pain 2012, **153**(7):1418-1425.

3. Alba-Delgado C, Mico JA, Sanchez-Blazquez P, Berrocoso E: **Analgesic antidepressants promote the responsiveness of locus coeruleus neurons to noxious stimulation: implications for neuropathic pain.** Pain 2012, **153**(7):1438-1449.

4. Amrutkar DV, Ploug KB, Hay-Schmidt A, Porreca F, Olesen J, Jansen-Olesen I: **mRNA expression of 5-hydroxytryptamine 1B, 1D, and 1F receptors and their role in controlling the release of calcitonin gene-related peptide in the rat trigeminovascular system.** Pain 2012, **153**(4):830-838.

5. Austin PJ, Kim CF, Perera CJ, Moalem-Taylor G: **Regulatory T cells attenuate neuropathic pain following peripheral nerve injury and experimental autoimmune neuritis.** Pain 2012, **153**(9):1916-1931.

6. Barriere DA, Rieusset J, Chanteranne D, Busserolles J, Chauvin MA, Chapuis L, Salles J, Dubray C, Morio B: **Paclitaxel therapy potentiates cold hyperalgesia in streptozotocin-induced diabetic rats through enhanced mitochondrial reactive oxygen species production and TRPA1 sensitization.** Pain 2012, **153**(3):553-561.

7. Beggs S, Alvares D, Moss A, Currie G, Middleton J, Salter MW, Fitzgerald M: **A role for NT-3 in the hyperinnervation of neonatally wounded skin.** Pain 2012, **153**(10):2133-2139.

8. Bosshard SC, Grandjean J, Schroeter A, Baltes C, Zeilhofer HU, Rudin M: **Hyperalgesia by low doses of the local anesthetic lidocaine involves cannabinoid signaling: an fMRI study in mice.** Pain 2012, **153**(7):1450-1458.

9. Chiou CS, Huang CC, Liang YC, Tsai YC, Hsu KS: **Impairment of long-term depression in the anterior cingulate cortex of mice with bone cancer pain.** Pain 2012, **153**(10):2097-2108.

10. Crown ED, Gwak YS, Ye Z, Yu Tan H, Johnson KM, Xu GY, McAdoo DJ, Hulsebosch CE: **Calcium/calmodulin dependent kinase II contributes to persistent central neuropathic pain following spinal cord injury.** Pain 2012, **153**(3):710-721.

11. Djouhri L, Fang X, Koutsikou S, Lawson SN: **Partial nerve injury induces electrophysiological changes in conducting (uninjured) nociceptive and nonnociceptive DRG neurons: Possible relationships to aspects of peripheral neuropathic pain and paresthesias.** Pain 2012, **153**(9):1824-1836.

12. Duan KZ, Xu Q, Zhang XM, Zhao ZQ, Mei YA, Zhang YQ: **Targeting A-type K(+) channels in primary sensory neurons for bone cancer pain in a rat model.** Pain 2012, **153**(3):562-574.

13. Edelmayer RM, Le LN, Yan J, Wei X, Nassini R, Materazzi S, Preti D, Appendino G, Geppetti P, Dodick DW, Vanderah TW, Porreca F, Dussor G: **Activation of TRPA1 on dural afferents: a potential mechanism of headache pain.** Pain 2012, **153**(9):1949-1958.

14. Franchi S, Valsecchi AE, Borsani E, Procacci P, Ferrari D, Zalfa C, Sartori P, Rodella LF, Vescovi A, Maione S, Rossi F, Sacerdote P, Colleoni M, Panerai AE: **Intravenous neural stem cells abolish nociceptive hypersensitivity and trigger nerve regeneration in experimental neuropathy.** Pain 2012, **153**(4):850-861.

15. Fukuoka T, Yamanaka H, Kobayashi K, Okubo M, Miyoshi K, Dai Y, Noguchi K: **Re-evaluation of the phenotypic changes in L4 dorsal root ganglion neurons after L5 spinal nerve ligation.** Pain 2012, **153**(1):68-79.

16. Gregoire S, Michaud V, Chapuy E, Eschalier A, Ardid D: **Study of emotional and cognitive impairments in mononeuropathic rats: effect of duloxetine and gabapentin.** Pain 2012, **153**(8):1657-1663.

17. Hathway GJ, Vega-Avelaira D, Fitzgerald M: **A critical period in the supraspinal control of pain: opioid-dependent changes in brainstem rostroventral medulla function in preadolescence.** Pain 2012, **153**(4):775-783.

18. Hoffmann J, Supronsinchai W, Andreou AP, Summ O, Akerman S, Goadsby PJ: **Olvanil acts on transient receptor potential vanilloid channel 1 and cannabinoid receptors to modulate neuronal transmission in the trigeminovascular system.** Pain 2012, **153**(11):2226-2232.

19. Huang ZJ, Li HC, Cowan AA, Liu S, Zhang YK, Song XJ: **Chronic compression or acute dissociation of dorsal root ganglion induces cAMP-dependent neuronal hyperexcitability through activation of PAR2.** Pain 2012, **153**(7):1426-1437.

20. Inquimbert P, Bartels K, Babaniyi OB, Barrett LB, Tegeder I, Scholz J: **Peripheral nerve injury produces a sustained shift in the balance between glutamate release and uptake in the dorsal horn of the spinal cord.** Pain 2012, **153**(12):2422-2431.

21. Jankowski MP, Rau KK, Soneji DJ, Ekmann KM, Anderson CE, Molliver DC, Koerber HR: **Purinergic receptor P2Y1 regulates polymodal C-fiber thermal thresholds and sensory neuron phenotypic switching during peripheral inflammation.** Pain 2012, **153**(2):410-419.

22. Ji Y, Tang B, Cao DY, Wang G, Traub RJ: **Sex differences in spinal processing of transient and inflammatory colorectal stimuli in the rat.** Pain 2012, **153**(9):1965-1973.

23. Kim Y, Cho HY, Ahn YJ, Kim J, Yoon YW: **Effect of NMDA NR2B antagonist on neuropathic pain in two spinal cord injury models.** Pain 2012, **153**(5):1022-1029.

24. King T, Qu C, Okun A, Melemedjian OK, Mandell EK, Maskaykina IY, Navratilova E, Dussor GO, Ghosh S, Price TJ, Porreca F: **Contribution of PKMzeta-dependent and independent amplification to components of experimental neuropathic pain.** Pain 2012, **153**(6):1263-1273.

25. Knights CB, Gentry C, Bevan S: **Partial medial meniscectomy produces osteoarthritis pain-related behaviour in female C57BL/6 mice.** Pain 2012, **153**(2):281-292.

26. Lee DZ, Chung JM, Chung K, Kang MG: **Reactive oxygen species (ROS) modulate AMPA receptor phosphorylation and cell-surface localization in concert with pain-related behavior.** Pain 2012, **153**(9):1905-1915.

27. Lee J, Saloman JL, Weiland G, Auh QS, Chung MK, Ro JY: **Functional interactions between NMDA receptors and TRPV1 in trigeminal sensory neurons mediate mechanical hyperalgesia in the rat masseter muscle.** Pain 2012, **153**(7):1514-1524.

28. Liang DY, Li X, Shi X, Sun Y, Sahbaie P, Li WW, Clark JD: **The complement component C5a receptor mediates pain and inflammation in a postsurgical pain model.** Pain 2012, **153**(2):366-372.

29. Liou JT, Yuan HB, Mao CC, Lai YS, Day YJ: **Absence of C-C motif chemokine ligand 5 in mice leads to decreased local macrophage recruitment and behavioral hypersensitivity in a murine neuropathic pain model.** Pain 2012, **153**(6):1283-1291.

30. Marshall TM, Herman DS, Largent-Milnes TM, Badghisi H, Zuber K, Holt SC, Lai J, Porreca F, Vanderah TW: **Activation of descending pain-facilitatory pathways from the rostral ventromedial medulla by cholecystokinin elicits release of prostaglandin-E(2) in the spinal cord.** Pain 2012, **153**(1):86-94.

31. Martuscello RT, Spengler RN, Bonoiu AC, Davidson BA, Helinski J, Ding H, Mahajan S, Kumar R, Bergey EJ, Knight PR, Prasad PN, Ignatowski TA: **Increasing TNF levels solely in the rat hippocampus produces persistent pain-like symptoms.** Pain 2012, **153**(9):1871-1882.

32. Michot B, Bourgoin S, Viguier F, Hamon M, Kayser V: **Differential effects of calcitonin gene-related peptide receptor blockade by olcegepant on mechanical allodynia induced by ligation of the infraorbital nerve vs the sciatic nerve in the rat.** Pain 2012, **153**(9):1939-1948.

33. Millecamps M, Tajerian M, Naso L, Sage EH, Stone LS: **Lumbar intervertebral disc degeneration associated with axial and radiating low back pain in ageing SPARC-null mice.** Pain 2012, **153**(6):1167-1179.

34. Nakajima K, Obata H, Iriuchijima N, Saito S: **An increase in spinal cord noradrenaline is a major contributor to the antihyperalgesic effect of antidepressants after peripheral nerve injury in the rat.** Pain 2012, **153**(5):990-997.

35. Niu KY, Zhang Y, Ro JY: **Effects of gonadal hormones on the peripheral cannabinoid receptor 1 (CB1R) system under a myositis condition in rats.** Pain 2012, **153**(11):2283-2291.

36. Nolan TA, Price DD, Caudle RM, Murphy NP, Neubert JK: **Placebo-induced analgesia in an operant pain model in rats.** Pain 2012, **153**(10):2009-2016.

37. Okun A, Liu P, Davis P, Ren J, Remeniuk B, Brion T, Ossipov MH, Xie J, Dussor GO, King T, Porreca F: **Afferent drive elicits ongoing pain in a model of advanced osteoarthritis.** Pain 2012, **153**(4):924-933.

38. Otsubo Y, Satoh Y, Kodama M, Araki Y, Satomoto M, Sakamoto E, Pages G, Pouyssegur J, Endo S, Kazama T: **Mechanical allodynia but not thermal hyperalgesia is impaired in mice deficient for ERK2 in the central nervous system.** Pain 2012, **153**(11):2241-2252.

39. Padi SS, Shi XQ, Zhao YQ, Ruff MR, Baichoo N, Pert CB, Zhang J: **Attenuation of rodent neuropathic pain by an orally active peptide, RAP-103, which potently blocks CCR2- and CCR5-mediated monocyte chemotaxis and inflammation.** Pain 2012, **153**(1):95-106.

40. Pagano RL, Fonoff ET, Dale CS, Ballester G, Teixeira MJ, Britto LR: **Motor cortex stimulation inhibits thalamic sensory neurons and enhances activity of PAG neurons: possible pathways for antinociception.** Pain 2012, **153**(12):2359-2369.

41. Pais-Vieira M, Aguiar P, Lima D, Galhardo V: **Inflammatory pain disrupts the orbitofrontal neuronal activity and risk-assessment performance in a rodent decision-making task.** Pain 2012, **153**(8):1625-1635.

42. Pelletier J, Fromy B, Morel G, Roquelaure Y, Saumet JL, Sigaudo-Roussel D: **Chronic sciatic nerve injury impairs the local cutaneous neurovascular interaction in rats.** Pain 2012, **153**(1):149-157.

43. Peng HY, Chen GD, Hsieh MC, Lai CY, Huang YP, Lin TB: **Spinal SGK1/GRASP-1/Rab4 is involved in complete Freund's adjuvant-induced inflammatory pain via regulating dorsal horn GluR1-containing AMPA receptor trafficking in rats.** Pain 2012, **153**(12):2380-2392.

44. Peng HY, Chen GD, Lai CY, Hsieh MC, Lin TB: **Spinal SIRPalpha1-SHP2 interaction regulates spinal nerve ligation-induced neuropathic pain via PSD-95-dependent NR2B activation in rats.** Pain 2012, **153**(5):1042-1053.

45. Reichl S, Augustin M, Zahn PK, Pogatzki-Zahn EM: **Peripheral and spinal GABAergic regulation of incisional pain in rats.** Pain 2012, **153**(1):129-141.

46. Rogoz K, Lagerstrom MC, Dufour S, Kullander K: **VGLUT2-dependent glutamatergic transmission in primary afferents is required for intact nociception in both acute and persistent pain modalities.** Pain 2012, **153**(7):1525-1536.

47. Ruparel S, Henry MA, Akopian A, Patil M, Zeldin DC, Roman L, Hargreaves KM: **Plasticity of cytochrome P450 isozyme expression in rat trigeminal ganglia neurons during inflammation.** Pain 2012, **153**(10):2031-2039.

48. Sasso O, Russo R, Vitiello S, Raso GM, D'Agostino G, Iacono A, Rana GL, Vallee M, Cuzzocrea S, Piazza PV, Meli R, Calignano A: **Implication of allopregnanolone in the antinociceptive effect of N-palmitoylethanolamide in acute or persistent pain.** Pain 2012, **153**(1):33-41.

49. Serra J, Bostock H, Sola R, Aleu J, Garcia E, Cokic B, Navarro X, Quiles C: **Microneurographic identification of spontaneous activity in C-nociceptors in neuropathic pain states in humans and rats.** Pain 2012, **153**(1):42-55.

50. Shields SD, Ahn HS, Yang Y, Han C, Seal RP, Wood JN, Waxman SG, Dib-Hajj SD: **Nav1.8 expression is not restricted to nociceptors in mouse peripheral nervous system.** Pain 2012, **153**(10):2017-2030.

51. Smits H, van Kleef M, Joosten EA: **Spinal cord stimulation of dorsal columns in a rat model of neuropathic pain: evidence for a segmental spinal mechanism of pain relief.** Pain 2012, **153**(1):177-183.

52. Song Y, Li HM, Xie RG, Yue ZF, Song XJ, Hu SJ, Xing JL: **Evoked bursting in injured Abeta dorsal root ganglion neurons: a mechanism underlying tactile allodynia.** Pain 2012, **153**(3):657-665.

53. Takasaki I, Taniguchi K, Komatsu F, Sasaki A, Andoh T, Nojima H, Shiraki K, Hsu DK, Liu FT, Kato I, Hiraga K, Kuraishi Y: **Contribution of spinal galectin-3 to acute herpetic allodynia in mice.** Pain 2012, **153**(3):585-592.

54. Tappe-Theodor A, Constantin CE, Tegeder I, Lechner SG, Langeslag M, Lepcynzsky P, Wirotanseng RI, Kurejova M, Agarwal N, Nagy G, Todd A, Wettschureck N, Offermanns S, Kress M, Lewin GR, Kuner R: **Galpha(q/11) signaling tonically modulates nociceptor function and contributes to activity-dependent sensitization.** Pain 2012, **153**(1):184-196.

55. Taylor AM, Osikowicz M, Ribeiro-da-Silva A: **Consequences of the ablation of nonpeptidergic afferents in an animal model of trigeminal neuropathic pain.** Pain 2012, **153**(6):1311-1319.

56. Thibault K, Calvino B, Dubacq S, Roualle-de-Rouville M, Sordoillet V, Rivals I, Pezet S: **Cortical effect of oxaliplatin associated with sustained neuropathic pain: exacerbation of cortical activity and down-regulation of potassium channel expression in somatosensory cortex.** Pain 2012, **153**(8):1636-1647.

57. Uhelski ML, Davis MA, Fuchs PN: **Pain affect in the absence of pain sensation: evidence of asomaesthesia after somatosensory cortex lesions in the rat.** Pain 2012, **153**(4):885-892.

58. Urtikova N, Berson N, Van Steenwinckel J, Doly S, Truchetto J, Maroteaux L, Pohl M, Conrath M: **Antinociceptive effect of peripheral serotonin 5-HT2B receptor activation on neuropathic pain.** Pain 2012, **153**(6):1320-1331.

59. Walder RY, Radhakrishnan R, Loo L, Rasmussen LA, Mohapatra DP, Wilson SP, Sluka KA: **TRPV1 is important for mechanical and heat sensitivity in uninjured animals and development of heat hypersensitivity after muscle inflammation.** Pain 2012, **153**(8):1664-1672.

60. Wang S, Tian Y, Song L, Lim G, Tan Y, You Z, Chen L, Mao J: **Exacerbated mechanical hyperalgesia in rats with genetically predisposed depressive behavior: role of melatonin and NMDA receptors.** Pain 2012, **153**(12):2448-2457.

61. Weng X, Smith T, Sathish J, Djouhri L: **Chronic inflammatory pain is associated with increased excitability and hyperpolarization-activated current (Ih) in C- but not Adelta-nociceptors.** Pain 2012, **153**(4):900-914.

62. Wilkerson JL, Gentry KR, Dengler EC, Wallace JA, Kerwin AA, Armijo LM, Kuhn MN, Thakur GA, Makriyannis A, Milligan ED: **Intrathecal cannabilactone CB(2)R agonist, AM1710, controls pathological pain and restores basal cytokine levels.** Pain 2012, **153**(5):1091-1106.

63. Xiao WH, Bennett GJ: **Effects of mitochondrial poisons on the neuropathic pain produced by the chemotherapeutic agents, paclitaxel and oxaliplatin.** Pain 2012, **153**(3):704-709.

**2. Anesthesiology**

1. Biais M, Jouffroy R, Carillion A, Feldman S, Jobart-Malfait A, Riou B, Amour J: **Interaction of metabolic and respiratory acidosis with alpha and beta-adrenoceptor stimulation in rat myocardium.** Anesthesiology 2012, **117**(6):1212-1222.

2. Bickler PE, Warren DE, Clark JP, Gabatto P, Gregersen M, Brosnan H: **Anesthetic protection of neurons injured by hypothermia and rewarming: roles of intracellular Ca2+ and excitotoxicity.** Anesthesiology 2012, **117**(2):280-292.

3. Blaudszun G, Morel DR: **Superiority of desflurane over sevoflurane and isoflurane in the presence of pressure-overload right ventricle hypertrophy in rats.** Anesthesiology 2012, **117**(5):1051-1061.

4. Borghese CM, Xiong W, Oh SI, Ho A, Mihic SJ, Zhang L, Lovinger DM, Homanics GE, Eger EI,2nd, Harris RA: **Mutations M287L and Q266I in the glycine receptor alpha1 subunit change sensitivity to volatile anesthetics in oocytes and neurons, but not the minimal alveolar concentration in knockin mice.** Anesthesiology 2012, **117**(4):765-771.

5. Bravo L, Mico JA, Rey-Brea R, Perez-Nievas B, Leza JC, Berrocoso E: **Depressive-like states heighten the aversion to painful stimuli in a rat model of comorbid chronic pain and depression.** Anesthesiology 2012, **117**(3):613-625.

6. Callaway JK, Jones NC, Royse AG, Royse CF: **Sevoflurane anesthesia does not impair acquisition learning or memory in the Morris water maze in young adult and aged rats.** Anesthesiology 2012, **117**(5):1091-1101.

7. Cao JP, He XY, Xu HT, Zou Z, Shi XY: **Autologous transplantation of peripheral blood-derived circulating endothelial progenitor cells attenuates endotoxin-induced acute lung injury in rabbits by direct endothelial repair and indirect immunomodulation.** Anesthesiology 2012, **116**(6):1278-1287.

8. Cao W, Pavlinec C, Gravenstein N, Seubert CN, Martynyuk AE: **Roles of aldosterone and oxytocin in abnormalities caused by sevoflurane anesthesia in neonatal rats.** Anesthesiology 2012, **117**(4):791-800.

9. Cayla C, Labuz D, Machelska H, Bader M, Schafer M, Stein C: **Impaired nociception and peripheral opioid antinociception in mice lacking both kinin B1 and B2 receptors.** Anesthesiology 2012, **116**(2):448-457.

10. Chemali JJ, Van Dort CJ, Brown EN, Solt K: **Active emergence from propofol general anesthesia is induced by methylphenidate.** Anesthesiology 2012, **116**(5):998-1005.

11. Dai S, Perouansky M, Pearce RA: **Isoflurane enhances both fast and slow synaptic inhibition in the hippocampus at amnestic concentrations.** Anesthesiology 2012, **116**(4):816-823.

12. Dong F, Xie W, Strong JA, Zhang JM: **Mineralocorticoid receptor blocker eplerenone reduces pain behaviors in vivo and decreases excitability in small-diameter sensory neurons from local inflamed dorsal root ganglia in vitro.** Anesthesiology 2012, **117**(5):1102-1112.

13. Ducrocq N, Kimmoun A, Furmaniuk A, Hekalo Z, Maskali F, Poussier S, Marie PY, Levy B: **Comparison of equipressor doses of norepinephrine, epinephrine, and phenylephrine on septic myocardial dysfunction.** Anesthesiology 2012, **116**(5):1083-1091.

14. Eikermann M, Grosse-Sundrup M, Zaremba S, Henry ME, Bittner EA, Hoffmann U, Chamberlin NL: **Ketamine activates breathing and abolishes the coupling between loss of consciousness and upper airway dilator muscle dysfunction.** Anesthesiology 2012, **116**(1):35-46.

15. Ewan EE, Martin TJ: **Intracranial self-stimulation of the paraventricular nucleus of the hypothalamus: increased faciliation by morphine compared to cocaine.** Anesthesiology 2012, **116**(5):1116-1123.

16. Faye N, Fournier L, Balvay D, Thiam R, Orliaguet G, Clement O, Dewachter P: **Macromolecular capillary leakage is involved in the onset of anaphylactic hypotension.** Anesthesiology 2012, **117**(5):1072-1079.

17. Ge R, Pejo E, Husain SS, Cotten JF, Raines DE: **Electroencephalographic and hypnotic recoveries after brief and prolonged infusions of etomidate and optimized soft etomidate analogs.** Anesthesiology 2012, **117**(5):1037-1043.

18. Godier A, Miclot A, Le Bonniec B, Durand M, Fischer AM, Emmerich J, Marchand-Leroux C, Lecompte T, Samama CM: **Evaluation of prothrombin complex concentrate and recombinant activated factor VII to reverse rivaroxaban in a rabbit model.** Anesthesiology 2012, **116**(1):94-102.

19. Guenther U, Theuerkauf NU, Huse D, Boettcher MF, Wensing G, Putensen C, Hoeft A: **Selective 5-HT(1A)-R-agonist repinotan prevents remifentanil-induced ventilatory depression and prolongs antinociception.** Anesthesiology 2012, **116**(1):56-64.

20. Hayashida K, Kimura M, Yoshizumi M, Hobo S, Obata H, Eisenach JC: **Ondansetron reverses antihypersensitivity from clonidine in rats after peripheral nerve injury: role of gamma-aminobutyric acid in alpha2-adrenoceptor and 5-HT3 serotonin receptor analgesia.** Anesthesiology 2012, **117**(2):389-398.

21. Hu FY, Hanna GM, Han W, Mardini F, Thomas SA, Wyner AJ, Kelz MB: **Hypnotic hypersensitivity to volatile anesthetics and dexmedetomidine in dopamine beta-hydroxylase knockout mice.** Anesthesiology 2012, **117**(5):1006-1017.

22. Husain SS, Pejo E, Ge R, Raines DE: **Modifying methoxycarbonyl etomidate inter-ester spacer optimizes in vitro metabolic stability and in vivo hypnotic potency and duration of action.** Anesthesiology 2012, **117**(5):1027-1036.

23. Ishikawa M, Tanaka S, Arai M, Genda Y, Sakamoto A: **Differences in microRNA changes of healthy rat liver between sevoflurane and propofol anesthesia.** Anesthesiology 2012, **117**(6):1245-1252.

24. James ML, Wang H, Cantillana V, Lei B, Kernagis DN, Dawson HN, Klaman LD, Laskowitz DT: **TT-301 inhibits microglial activation and improves outcome after central nervous system injury in adult mice.** Anesthesiology 2012, **116**(6):1299-1311.

25. Jee JP, Parlato MC, Perkins MG, Mecozzi S, Pearce RA: **Exceptionally stable fluorous emulsions for the intravenous delivery of volatile general anesthetics.** Anesthesiology 2012, **116**(3):580-585.

26. Koeppen M, Harter PN, Bonney S, Bonney M, Reithel S, Zachskorn C, Mittelbronn M, Eckle T: **Adora2b signaling on bone marrow derived cells dampens myocardial ischemia-reperfusion injury.** Anesthesiology 2012, **116**(6):1245-1257.

27. Kratzer S, Mattusch C, Kochs E, Eder M, Haseneder R, Rammes G: **Xenon attenuates hippocampal long-term potentiation by diminishing synaptic and extrasynaptic N-methyl-D-aspartate receptor currents.** Anesthesiology 2012, **116**(3):673-682.

28. Krishnamoorthy V, Hiller DB, Ripper R, Lin B, Vogel SM, Feinstein DL, Oswald S, Rothschild L, Hensel P, Rubinstein I, Minshall R, Weinberg GL: **Epinephrine induces rapid deterioration in pulmonary oxygen exchange in intact, anesthetized rats: a flow and pulmonary capillary pressure-dependent phenomenon.** Anesthesiology 2012, **117**(4):745-754.

29. Le Freche H, Brouillette J, Fernandez-Gomez FJ, Patin P, Caillierez R, Zommer N, Sergeant N, Buee-Scherrer V, Lebuffe G, Blum D, Buee L: **Tau phosphorylation and sevoflurane anesthesia: an association to postoperative cognitive impairment.** Anesthesiology 2012, **116**(4):779-787.

30. Lei C, Yu B, Shahid M, Beloiartsev A, Bloch KD, Zapol WM: **Inhaled nitric oxide attenuates the adverse effects of transfusing stored syngeneic erythrocytes in mice with endothelial dysfunction after hemorrhagic shock.** Anesthesiology 2012, **117**(6):1190-1202.

31. Li J, Iorga A, Sharma S, Youn JY, Partow-Navid R, Umar S, Cai H, Rahman S, Eghbali M: **Intralipid, a clinically safe compound, protects the heart against ischemia-reperfusion injury more efficiently than cyclosporine-A.** Anesthesiology 2012, **117**(4):836-846.

32. Li T, Lin X, Zhu Y, Li L, Liu L: **Short-term, mild hypothermia can increase the beneficial effect of permissive hypotension on uncontrolled hemorrhagic shock in rats.** Anesthesiology 2012, **116**(6):1288-1298.

33. Li T, Zhu Y, Fang Y, Liu L: **Determination of the optimal mean arterial pressure for postbleeding resuscitation after hemorrhagic shock in rats.** Anesthesiology 2012, **116**(1):103-112.

34. Li WW, Guo TZ, Liang DY, Sun Y, Kingery WS, Clark JD: **Substance P signaling controls mast cell activation, degranulation, and nociceptive sensitization in a rat fracture model of complex regional pain syndrome.** Anesthesiology 2012, **116**(4):882-895.

35. Liu JR, Liu Q, Li J, Baek C, Han XH, Athiraman U, Soriano SG: **Noxious stimulation attenuates ketamine-induced neuroapoptosis in the developing rat brain.** Anesthesiology 2012, **117**(1):64-71.

36. Lonati C, Sordi A, Giuliani D, Spaccapelo L, Leonardi P, Carlin A, Ottani A, Galantucci M, Grieco P, Catania A, Guarini S: **Molecular changes induced in rat liver by hemorrhage and effects of melanocortin treatment.** Anesthesiology 2012, **116**(3):692-700.

37. Lucchinetti E, Awad AE, Rahman M, Feng J, Lou PH, Zhang L, Ionescu L, Lemieux H, Thebaud B, Zaugg M: **Antiproliferative effects of local anesthetics on mesenchymal stem cells: potential implications for tumor spreading and wound healing.** Anesthesiology 2012, **116**(4):841-856.

38. Ma F, Zhang L, Westlund KN: **Trigeminal nerve injury ErbB3/ErbB2 promotes mechanical hypersensitivity.** Anesthesiology 2012, **117**(2):381-388.

39. Mrozek S, Jung B, Petrof BJ, Pauly M, Roberge S, Lacampagne A, Cassan C, Thireau J, Molinari N, Futier E, Scheuermann V, Constantin JM, Matecki S, Jaber S: **Rapid onset of specific diaphragm weakness in a healthy murine model of ventilator-induced diaphragmatic dysfunction.** Anesthesiology 2012, **117**(3):560-567.

40. Pearn ML, Hu Y, Niesman IR, Patel HH, Drummond JC, Roth DM, Akassoglou K, Patel PM, Head BP: **Propofol neurotoxicity is mediated by p75 neurotrophin receptor activation.** Anesthesiology 2012, **116**(2):352-361.

41. Pejo E, Ge R, Banacos N, Cotten JF, Husain SS, Raines DE: **Electroencephalographic recovery, hypnotic emergence, and the effects of metabolite after continuous infusions of a rapidly metabolized etomidate analog in rats.** Anesthesiology 2012, **116**(5):1057-1065.

42. Pickerodt PA, Emery MJ, Zarndt R, Martin W, Francis RC, Boemke W, Swenson ER: **Sodium nitrite mitigates ventilator-induced lung injury in rats.** Anesthesiology 2012, **117**(3):592-601.

43. Prakash A, Mesa KR, Wilhelmsen K, Xu F, Dodd-o JM, Hellman J: **Alveolar macrophages and Toll-like receptor 4 mediate ventilated lung ischemia reperfusion injury in mice.** Anesthesiology 2012, **117**(4):822-835.

44. Ren BX, Gu XP, Zheng YG, Liu CL, Wang D, Sun YE, Ma ZL: **Intrathecal injection of metabotropic glutamate receptor subtype 3 and 5 agonist/antagonist attenuates bone cancer pain by inhibition of spinal astrocyte activation in a mouse model.** Anesthesiology 2012, **116**(1):122-132.

45. Rittner HL, Amasheh S, Moshourab R, Hackel D, Yamdeu RS, Mousa SA, Fromm M, Stein C, Brack A: **Modulation of tight junction proteins in the perineurium to facilitate peripheral opioid analgesia.** Anesthesiology 2012, **116**(6):1323-1334.

46. Sahbaie P, Li X, Shi X, Clark JD: **Roles of Gr-1+ leukocytes in postincisional nociceptive sensitization and inflammation.** Anesthesiology 2012, **117**(3):602-612.

47. Sall JW, Stratmann G, Leong J, Woodward E, Bickler PE: **Propofol at clinically relevant concentrations increases neuronal differentiation but is not toxic to hippocampal neural precursor cells in vitro.** Anesthesiology 2012, **117**(5):1080-1090.

48. Schellekens WJ, van Hees HW, Vaneker M, Linkels M, Dekhuijzen PN, Scheffer GJ, van der Hoeven JG, Heunks LM: **Toll-like receptor 4 signaling in ventilator-induced diaphragm atrophy.** Anesthesiology 2012, **117**(2):329-338.

49. Serra MF, Anjos-Valotta EA, Olsen PC, Couto GC, Jurgilas PB, Cotias AC, Pao CR, Ferreira TP, Arantes AC, Pires AL, Cordeiro RS, Silva PM, Martins MA: **Nebulized lidocaine prevents airway inflammation, peribronchial fibrosis, and mucus production in a murine model of asthma.** Anesthesiology 2012, **117**(3):580-591.

50. Shen J, Fox LE, Cheng J: **Differential effects of peripheral versus central coadministration of QX-314 and capsaicin on neuropathic pain in rats.** Anesthesiology 2012, **117**(2):365-380.

51. Sheng SP, Lei B, James ML, Lascola CD, Venkatraman TN, Jung JY, Maze M, Franks NP, Pearlstein RD, Sheng H, Warner DS: **Xenon neuroprotection in experimental stroke: interactions with hypothermia and intracerebral hemorrhage.** Anesthesiology 2012, **117**(6):1262-1275.

52. Shih J, May LD, Gonzalez HE, Lee EW, Alvi RS, Sall JW, Rau V, Bickler PE, Lalchandani GR, Yusupova M, Woodward E, Kang H, Wilk AJ, Carlston CM, Mendoza MV, Guggenheim JN, Schaefer M, Rowe AM, Stratmann G: **Delayed environmental enrichment reverses sevoflurane-induced memory impairment in rats.** Anesthesiology 2012, **116**(3):586-602.

53. Soares JH, Brosnan RJ, Fukushima FB, Hodges J, Liu H: **Solubility of haloether anesthetics in human and animal blood.** Anesthesiology 2012, **117**(1):48-55.

54. Song JG, Li HH, Cao YF, Lv X, Zhang P, Li YS, Zheng YJ, Li Q, Yin PH, Song SL, Wang HY, Wang XR: **Electroacupuncture improves survival in rats with lethal endotoxemia via the autonomic nervous system.** Anesthesiology 2012, **116**(2):406-414.

55. Sorensen H, Secher NH, Siebenmann C, Nielsen HB, Kohl-Bareis M, Lundby C, Rasmussen P: **Cutaneous vasoconstriction affects near-infrared spectroscopy determined cerebral oxygen saturation during administration of norepinephrine.** Anesthesiology 2012, **117**(2):263-270.

56. Spofford CM, Brennan TJ: **Gene expression in skin, muscle, and dorsal root ganglion after plantar incision in the rat.** Anesthesiology 2012, **117**(1):161-172.

57. Sun Y, Li XQ, Sahbaie P, Shi XY, Li WW, Liang DY, Clark JD: **miR-203 regulates nociceptive sensitization after incision by controlling phospholipase A2 activating protein expression.** Anesthesiology 2012, **117**(3):626-638.

58. Suzuki M, Narita M, Hasegawa M, Furuta S, Kawamata T, Ashikawa M, Miyano K, Yanagihara K, Chiwaki F, Ochiya T, Suzuki T, Matoba M, Sasaki H, Uezono Y: **Sensation of abdominal pain induced by peritoneal carcinomatosis is accompanied by changes in the expression of substance P and mu-opioid receptors in the spinal cord of mice.** Anesthesiology 2012, **117**(4):847-856.

59. Torigoe K, Nakahara K, Rahmadi M, Yoshizawa K, Horiuchi H, Hirayama S, Imai S, Kuzumaki N, Itoh T, Yamashita A, Shakunaga K, Yamasaki M, Nagase H, Matoba M, Suzuki T, Narita M: **Usefulness of olanzapine as an adjunct to opioid treatment and for the treatment of neuropathic pain.** Anesthesiology 2012, **116**(1):159-169.

60. Tsai YJ, Huang CT, Lin SC, Yeh JH: **Effects of regional and whole-body hypothermic treatment before and after median nerve injury on neuropathic pain and glial activation in rat cuneate nucleus.** Anesthesiology 2012, **116**(2):415-431.

61. Wei H, Karimaa M, Korjamo T, Koivisto A, Pertovaara A: **Transient receptor potential ankyrin 1 ion channel contributes to guarding pain and mechanical hypersensitivity in a rat model of postoperative pain.** Anesthesiology 2012, **117**(1):137-148.

62. Werdehausen R, Kremer D, Brandenburger T, Schlosser L, Jadasz J, Kury P, Bauer I, Aragon C, Eulenburg V, Hermanns H: **Lidocaine metabolites inhibit glycine transporter 1: a novel mechanism for the analgesic action of systemic lidocaine?** Anesthesiology 2012, **116**(1):147-158.

63. Wulfert FM, van Meurs M, Kurniati NF, Jongman RM, Houwertjes MC, Heeringa P, Struys MM, Zijlstra JG, Molema G: **Age-dependent role of microvascular endothelial and polymorphonuclear cells in lipopolysaccharide-induced acute kidney injury.** Anesthesiology 2012, **117**(1):126-136.

64. Yamamoto T, Honda H, Baba H, Kohno T: **Effect of xenon on excitatory and inhibitory transmission in rat spinal ventral horn neurons.** Anesthesiology 2012, **116**(5):1025-1034.

65. Yang Q, Yan W, Li X, Hou L, Dong H, Wang Q, Dong H, Wang S, Zhang X, Xiong L: **Activation of canonical notch signaling pathway is involved in the ischemic tolerance induced by sevoflurane preconditioning in mice.** Anesthesiology 2012, **117**(5):996-1005.

66. Yoshizumi M, Parker RA, Eisenach JC, Hayashida K: **Gabapentin inhibits gamma-amino butyric acid release in the locus coeruleus but not in the spinal dorsal horn after peripheral nerve injury in rats.** Anesthesiology 2012, **116**(6):1347-1353.

67. Zaugg M, Wang L, Zhang L, Lou PH, Lucchinetti E, Clanachan AS: **Choice of anesthetic combination determines Ca2+ leak after ischemia-reperfusion injury in the working rat heart: favorable versus adverse combinations.** Anesthesiology 2012, **116**(3):648-657.

68. Zhang J, Li H, Teng H, Zhang T, Luo Y, Zhao M, Li YQ, Sun ZS: **Regulation of peripheral clock to oscillation of substance P contributes to circadian inflammatory pain.** Anesthesiology 2012, **117**(1):149-160.

69. Zhang XY, Liu ZM, Wen SH, Li YS, Li Y, Yao X, Huang WQ, Liu KX: **Dexmedetomidine administration before, but not after, ischemia attenuates intestinal injury induced by intestinal ischemia-reperfusion in rats.** Anesthesiology 2012, **116**(5):1035-1046.

70. Zlotnik A, Sinelnikov I, Gruenbaum BF, Gruenbaum SE, Dubilet M, Dubilet E, Leibowitz A, Ohayon S, Regev A, Boyko M, Shapira Y, Teichberg VI: **Effect of glutamate and blood glutamate scavengers oxaloacetate and pyruvate on neurological outcome and pathohistology of the hippocampus after traumatic brain injury in rats.** Anesthesiology 2012, **116**(1):73-83.

**3. British Journal of Anaesthesia**

1. Dietis N, McDonald J, Molinari S, Calo G, Guerrini R, Rowbotham DJ, Lambert DG: **Pharmacological characterization of the bifunctional opioid ligand H-Dmt-Tic-Gly-NH-Bzl (UFP-505).** Br J Anaesth 2012, **108**(2):262-270.

2. Dyson A, Cone S, Singer M, Ackland GL: **Microvascular and macrovascular flow are uncoupled in early polymicrobial sepsis.** Br J Anaesth 2012, **108**(6):973-978.

3. Feng X, Liu JJ, Zhou X, Song FH, Yang XY, Chen XS, Huang WQ, Zhou LH, Ye JH: **Single sevoflurane exposure decreases neuronal nitric oxide synthase levels in the hippocampus of developing rats.** Br J Anaesth 2012, **109**(2):225-233.

4. Goyagi T, Tobe Y, Nishikawa T: **Long-term and spatial memory effects of selective beta1-antagonists after transient focal ischaemia in rats.** Br J Anaesth 2012, **109**(3):399-406.

5. Hahm TS, Ahn HJ, Ryu S, Gwak MS, Choi SJ, Kim JK, Yu JM: **Combined carbamazepine and pregabalin therapy in a rat model of neuropathic pain.** Br J Anaesth 2012, **109**(6):968-974.

6. Hama-Tomioka K, Kinoshita H, Nakahata K, Kondo T, Azma T, Kawahito S, Hatakeyama N, Matsuda N: **Roles of neuronal nitric oxide synthase, oxidative stress, and propofol in N-methyl-D-aspartate-induced dilatation of cerebral arterioles.** Br J Anaesth 2012, **108**(1):21-29.

7. Iwata M, Inoue S, Kawaguchi M, Furuya H: **Effects of diazepam and flumazenil on forebrain ischaemia in a rat model of benzodiazepine tolerance.** Br J Anaesth 2012, **109**(6):935-942.

8. Kassam SI, Lu C, Buckley N, Gao YJ, Lee RM: **Modulation of thiopental-induced vascular relaxation and contraction by perivascular adipose tissue and endothelium.** Br J Anaesth 2012, **109**(2):177-184.

9. Kojima A, Kitagawa H, Omatsu-Kanbe M, Matsuura H, Nosaka S: **Presence of store-operated Ca2+ entry in C57BL/6J mouse ventricular myocytes and its suppression by sevoflurane.** Br J Anaesth 2012, **109**(3):352-360.

10. Kortelainen J, Jia X, Seppanen T, Thakor N: **Increased electroencephalographic gamma activity reveals awakening from isoflurane anaesthesia in rats.** Br J Anaesth 2012, **109**(5):782-789.

11. Lawton BK, Brown NJ, Reilly CS, Brookes ZL: **Role of L-type calcium channels in altered microvascular responses to propofol in hypertension.** Br J Anaesth 2012, **108**(6):929-935.

12. Le Sache F, Le Bonniec B, Gaussem P, Dizier B, Tagzirt M, Godier A, Emmerich J, Samama CM: **Recombinant activated factor VII and prothrombin complex concentrates have different effects on bleeding and arterial thrombosis in the haemodiluted rabbit.** Br J Anaesth 2012, **108**(4):586-593.

13. Li B, Yan J, Shen Y, Li B, Hu Z, Ma Z: **Association of sustained cardiovascular recovery with epinephrine in the delayed lipid-based resuscitation from cardiac arrest induced by bupivacaine overdose in rats.** Br J Anaesth 2012, **108**(5):857-863.

14. Mahjoub Y, Lorne E, Maizel J, Plantefeve G, Massy ZA, Dupont H, Slama M: **Effect of intra-abdominal hypertension on left ventricular relaxation: a preliminary animal study.** Br J Anaesth 2012, **108**(2):211-215.

15. Pearce RA, Duscher P, Van Dyke K, Lee M, Andrei AC, Perouansky M: **Isoflurane impairs odour discrimination learning in rats: differential effects on short- and long-term memory.** Br J Anaesth 2012, **108**(4):630-637.

16. Ribeiro PO, Valentim AM, Rodrigues P, Olsson IA, Antunes LM: **Apoptotic neurodegeneration and spatial memory are not affected by sedative and anaesthetics doses of ketamine/medetomidine combinations in adult mice.** Br J Anaesth 2012, **108**(5):807-814.

17. Stumpner J, Lange M, Beck A, Smul TM, Lotz CA, Kehl F, Roewer N, Redel A: **Desflurane-induced post-conditioning against myocardial infarction is mediated by calcium-activated potassium channels: role of the mitochondrial permeability transition pore.** Br J Anaesth 2012, **108**(4):594-601.

18. Suto T, Obata H, Tobe M, Oku H, Yokoo H, Nakazato Y, Saito S: **Long-term effect of epidural injection with sustained-release lidocaine particles in a rat model of postoperative pain.** Br J Anaesth 2012, **109**(6):957-967.

19. Tan PH, Gao YJ, Berta T, Xu ZZ, Ji RR: **Short small-interfering RNAs produce interferon-alpha-mediated analgesia.** Br J Anaesth 2012, **108**(4):662-669.

20. Wang Y, Wong GT, Man K, Irwin MG: **Pretreatment with intrathecal or intravenous morphine attenuates hepatic ischaemia-reperfusion injury in normal and cirrhotic rat liver.** Br J Anaesth 2012, **109**(4):529-539.

21. Yue W, Guo Z: **Blockade of spinal nerves inhibits expression of neural growth factor in the myocardium at an early stage of acute myocardial infarction in rats.** Br J Anaesth 2012, **109**(3):345-351.

22. Zhang Y, Zhang RX, Zhang M, Shen XY, Li A, Xin J, Ren K, Berman BM, Tan M, Lao L: **Electroacupuncture inhibition of hyperalgesia in an inflammatory pain rat model: involvement of distinct spinal serotonin and norepinephrine receptor subtypes.** Br J Anaesth 2012, **109**(2):245-252.

**4. Anaesthesia**

1. Brendt P, Rehfeld I, Kamphausen A, Kreissig C, Peters J: **Lipopolysaccharide interference in erythropoiesis in mice.** Anaesthesia 2012, **67**(5):493-500.

2. Chen JJ, Hung KC, Lu K, Yu SW, Chang CC, Liu CC, Spielberger J, Ku PY, Tan PH: **The pre-emptive analgesic effect of a cyclooxygenase-2 inhibitor in a rat model of acute postoperative pain.** Anaesthesia 2012, **67**(11):1225-1231.

3. Tomak Y, Yilmaz A, Bostan H, Tumkaya L, Altuner D, Kalkan Y, Erdivanli B: **Effects of sugammadex and rocuronium mast cell number and degranulation in rat liver.** Anaesthesia 2012, **67**(10):1101-1104.

**5. Regional Anesthesia and Pain Medicine**

1. Doan L, Piskoun B, Rosenberg AD, Blanck TJ, Phillips MS, Xu F: **In vitro antiseptic effects on viability of neuronal and Schwann cells.** Reg Anesth Pain Med 2012, **37**(2):131-138.

2. Engle MP, Ness TJ, Robbins MT: **Intrathecal oxytocin inhibits visceromotor reflex and spinal neuronal responses to noxious distention of the rat urinary bladder.** Reg Anesth Pain Med 2012, **37**(5):515-520.

3. Hung CH, Chu CC, Chen YC, Liu KS, Chen YW, Wang JJ: **Cutaneous analgesia and systemic toxicity of carbetapentane and caramiphen in rats.** Reg Anesth Pain Med 2012, **37**(1):34-39.

4. Kroin JS, Buvanendran A, Tuman KJ, Kerns JM: **Effect of acute versus continuous glycemic control on duration of local anesthetic sciatic nerve block in diabetic rats.** Reg Anesth Pain Med 2012, **37**(6):595-600.

5. Leng F, Wan J, Liu W, Tao B, Chen X: **Prolongation of epidural analgesia using solid lipid nanoparticles as drug carrier for lidocaine.** Reg Anesth Pain Med 2012, **37**(2):159-165.

6. Lirk P, Flatz M, Haller I, Hausott B, Blumenthal S, Stevens MF, Suzuki S, Klimaschewski L, Gerner P: **In Zucker diabetic fatty rats, subclinical diabetic neuropathy increases in vivo lidocaine block duration but not in vitro neurotoxicity.** Reg Anesth Pain Med 2012, **37**(6):601-606.

7. Meleine M, Rivat C, Laboureyras E, Cahana A, Richebe P: **Sciatic nerve block fails in preventing the development of late stress-induced hyperalgesia when high-dose fentanyl is administered perioperatively in rats.** Reg Anesth Pain Med 2012, **37**(4):448-454.

8. Ohri R, Blaskovich P, Wang JC, Pham L, Nichols G, Hildebrand W, Costa D, Scarborough N, Herman C, Strichartz G: **Prolonged nerve block by microencapsulated bupivacaine prevents acute postoperative pain in rats.** Reg Anesth Pain Med 2012, **37**(6):607-615.

9. Shankarappa SA, Sagie I, Tsui JH, Chiang HH, Stefanescu C, Zurakowski D, Kohane DS: **Duration and local toxicity of sciatic nerve blockade with coinjected site 1 sodium-channel blockers and quaternary lidocaine derivatives.** Reg Anesth Pain Med 2012, **37**(5):483-489.

10. Stopar Pintaric T, Veranic P, Hadzic A, Karmakar M, Cvetko E: **Electron-microscopic imaging of endothoracic fascia in the thoracic paravertebral space in rats.** Reg Anesth Pain Med 2012, **37**(2):215-218.

11. Yilmaz-Rastoder E, Gold MS, Hough KA, Gebhart GF, Williams BA: **Effect of adjuvant drugs on the action of local anesthetics in isolated rat sciatic nerves.** Reg Anesth Pain Med 2012, **37**(4):403-409.

**6. Anesthesia and Analgesia**

1. Aguado D, Abreu M, Benito J, Garcia-Fernandez J, Gomez de Segura IA: **The effects of gabapentin on acute opioid tolerance to remifentanil under sevoflurane anesthesia in rats.** Anesth Analg 2012, **115**(1):40-45.

2. Antonialli Cde S, da Silva GF, Rocha LW, Monteiro ER, de Souza MM, Malheiros A, Yunes RA, Quintao NL: **Antihyperalgesic effects of myrsinoic acid B in pain-like behavior induced by inflammatory and neuropathic pain models in mice.** Anesth Analg 2012, **115**(2):461-469.

3. Araki Y, Kaibori M, Matsumura S, Kwon AH, Ito S: **Novel strategy for the control of postoperative pain: long-lasting effect of an implanted analgesic hydrogel in a rat model of postoperative pain.** Anesth Analg 2012, **114**(6):1338-1345.

4. Benggon M, Chen H, Applegate R, Martin R, Zhang JH: **Effect of dexmedetomidine on brain edema and neurological outcomes in surgical brain injury in rats.** Anesth Analg 2012, **115**(1):154-159.

5. Cereda CM, Tofoli GR, Maturana LG, Pierucci A, Nunes LA, Franz-Montan M, de Oliveira AL, Arana S, de Araujo DR, de Paula E: **Local neurotoxicity and myotoxicity evaluation of cyclodextrin complexes of bupivacaine and ropivacaine.** Anesth Analg 2012, **115**(5):1234-1241.

6. Chen YW, Li YT, Chen YC, Li ZY, Hung CH: **Exercise training attenuates neuropathic pain and cytokine expression after chronic constriction injury of rat sciatic nerve.** Anesth Analg 2012, **114**(6):1330-1337.

7. de Graaf W, Diepenhorst GM, Herroeder S, Erdogan D, Hollmann MW, van Gulik TM: **Systemic lidocaine does not attenuate hepatic dysfunction after liver surgery in rats.** Anesth Analg 2012, **114**(3):566-573.

8. Faller S, Strosing KM, Ryter SW, Buerkle H, Loop T, Schmidt R, Hoetzel A: **The volatile anesthetic isoflurane prevents ventilator-induced lung injury via phosphoinositide 3-kinase/Akt signaling in mice.** Anesth Analg 2012, **114**(4):747-756.

9. Haile M, Galoyan S, Li YS, Cohen BH, Quartermain D, Blanck T, Bekker A: **Nimodipine-induced hypotension but not nitroglycerin-induced hypotension preserves long- and short-term memory in adult mice.** Anesth Analg 2012, **114**(5):1034-1041.

10. Han H, Lee KS, Rong W, Zhang G: **Different roles of peripheral mitogen-activated protein kinases in carrageenan-induced arthritic pain and arthritis in rats.** Anesth Analg 2012, **115**(5):1221-1227.

11. Hobo S, Hayashida K, Eisenach JC: **Oxytocin inhibits the membrane depolarization-induced increase in intracellular calcium in capsaicin sensitive sensory neurons: a peripheral mechanism of analgesic action.** Anesth Analg 2012, **114**(2):442-449.

12. Honda H, Kawasaki Y, Baba H, Kohno T: **The mu opioid receptor modulates neurotransmission in the rat spinal ventral horn.** Anesth Analg 2012, **115**(3):703-712.

13. Ishida R, Nikai T, Hashimoto T, Tsumori T, Saito Y: **Intravenous infusion of remifentanil induces transient withdrawal hyperalgesia depending on administration duration in rats.** Anesth Analg 2012, **114**(1):224-229.

14. Jang Y, Song HK, Yeom MY, Jeong DC: **The immunomodulatory effect of pregabalin on spleen cells in neuropathic mice.** Anesth Analg 2012, **115**(4):830-836.

15. Kawano T, Tanaka K, Chi H, Eguchi S, Yamazaki F, Kitamura S, Kumagai N, Yokoyama M: **Biophysical and pharmacological properties of glucagon-like peptide-1 in rats under isoflurane anesthesia.** Anesth Analg 2012, **115**(1):62-69.

16. Kim J, Hwang J, Huh J, Nahm SF, Lim C, Park S, Hahn S: **Acute normovolemic hemodilution can aggravate neurological injury after spinal cord ischemia in rats.** Anesth Analg 2012, **114**(6):1285-1291.

17. Kim WM, Lee SH, Jeong HJ, Lee HG, Choi JI, Yoon MH: **The analgesic activity of intrathecal tianeptine, an atypical antidepressant, in a rat model of inflammatory pain.** Anesth Analg 2012, **114**(3):683-689.

18. Kimura M, Obata H, Saito S: **Antihypersensitivity effects of tramadol hydrochloride in a rat model of postoperative pain.** Anesth Analg 2012, **115**(2):443-449.

19. Kitamura T, Sato K, Kawamura G, Yamada Y: **The involvement of adenosine triphosphate-sensitive potassium channels in the different effects of sevoflurane and propofol on glucose metabolism in fed rats.** Anesth Analg 2012, **114**(1):110-116.

20. Kuster I, Kuschnereit R, Kelly M, Zhou J, Whynot S, Kianian M, Hung O, Shukla R, Cerny V, Pavlovic D, Lehmann C: **Cannabinoid receptor 1 inhibition causes seizures during anesthesia induction in experimental sepsis.** Anesth Analg 2012, **114**(6):1217-1219.

21. Li Z, Chen X, Meng J, Deng L, Ma H, Csete M, Xiong L: **ED50 and recovery times after propofol in rats with graded cirrhosis.** Anesth Analg 2012, **114**(1):117-121.

22. Lilius TO, Rauhala PV, Kambur O, Rossi SM, Vaananen AJ, Kalso EA: **Intrathecal atipamezole augments the antinociceptive effect of morphine in rats.** Anesth Analg 2012, **114**(6):1353-1358.

23. Liu L, Xia Y, Chen Y, Wang Q, Shi T, Wang F, Small RH, Xu X: **The comparative effects of lipid, epinephrine, and their combination in the reversal of bupivacaine-induced asystole in the isolated rat heart.** Anesth Analg 2012, **114**(4):886-893.

24. Mattioli TA, Sutak M, Milne B, Jhamandas K, Cahill CM: **Intrathecal catheterization influences tolerance to chronic morphine in rats.** Anesth Analg 2012, **114**(3):690-693.

25. Mihara T, Kikuchi T, Kamiya Y, Koga M, Uchimoto K, Kurahashi K, Goto T: **Day or night administration of ketamine and pentobarbital differentially affect circadian rhythms of pineal melatonin secretion and locomotor activity in rats.** Anesth Analg 2012, **115**(4):805-813.

26. Miyazaki R, Yamamoto T: **The efficacy of morphine, pregabalin, gabapentin, and duloxetine on mechanical allodynia is different from that on neuroma pain in the rat neuropathic pain model.** Anesth Analg 2012, **115**(1):182-188.

27. Moon JY, Song S, Yoon SY, Roh DH, Kang SY, Park JH, Beitz AJ, Lee JH: **The differential effect of intrathecal Nav1.8 blockers on the induction and maintenance of capsaicin- and peripheral ischemia-induced mechanical allodynia and thermal hyperalgesia.** Anesth Analg 2012, **114**(1):215-223.

28. Naik AK, Latham JR, Obradovic A, Jevtovic-Todorovic V: **Dorsal root ganglion application of muscimol prevents hyperalgesia and stimulates myelin protein expression after sciatic nerve injury in rats.** Anesth Analg 2012, **114**(3):674-682.

29. Narai Y, Imamachi N, Saito Y: **Gabapentin augments the antihyperalgesic effects of diclofenac sodium through spinal action in a rat postoperative pain model.** Anesth Analg 2012, **115**(1):189-193.

30. Pal D, Walton ME, Lipinski WJ, Koch LG, Lydic R, Britton SL, Mashour GA: **Determination of minimum alveolar concentration for isoflurane and sevoflurane in a rodent model of human metabolic syndrome.** Anesth Analg 2012, **114**(2):297-302.

31. Patwardhan A, Edelmayer R, Annabi E, Price T, Malan P, Dussor G: **Receptor specificity defines algogenic properties of propofol and fospropofol.** Anesth Analg 2012, **115**(4):837-840.

32. Pejo E, Cotten JF, Kelly EW, Le Ge R, Cuny GD, Laha JK, Liu J, Lin XJ, Raines DE: **In vivo and in vitro pharmacological studies of methoxycarbonyl-carboetomidate.** Anesth Analg 2012, **115**(2):297-304.

33. Petrovszki Z, Kovacs G, Tomboly C, Benedek G, Horvath G: **The effects of peptide and lipid endocannabinoids on arthritic pain at the spinal level.** Anesth Analg 2012, **114**(6):1346-1352.

34. Steppan J, Nyhan SM, Sikka G, Uribe J, Ahuja A, White AR, Shoukas AA, Berkowitz DE: **Vasopressin-mediated enhancement of adrenergic vasoconstriction involves both the tyrosine kinase and the protein kinase C pathways.** Anesth Analg 2012, **115**(6):1290-1295.

36. Tsai RY, Chou KY, Shen CH, Chien CC, Tsai WY, Huang YN, Tao PL, Lin YS, Wong CS: **Resveratrol regulates N-methyl-D-aspartate receptor expression and suppresses neuroinflammation in morphine-tolerant rats.** Anesth Analg 2012, **115**(4):944-952.

37. van der Vijver RJ, van Laarhoven CJ, Lomme RM, Hendriks T: **Paracetamol does not compromise early wound repair in the intestine or abdominal wall in the rat.** Anesth Analg 2012, **115**(6):1451-1456.

38. Wala EP, Crooks PA, McIntosh JM, Holtman JR,Jr: **Novel small molecule alpha9alpha10 nicotinic receptor antagonist prevents and reverses chemotherapy-evoked neuropathic pain in rats.** Anesth Analg 2012, **115**(3):713-720.

39. Walker SM, Grafe M, Yaksh TL: **Intrathecal clonidine in the neonatal rat: dose-dependent analgesia and evaluation of spinal apoptosis and toxicity.** Anesth Analg 2012, **115**(2):450-460.

40. Yeh YC, Sun WZ, Ko WJ, Chan WS, Fan SZ, Tsai JC, Lin TY: **Dexmedetomidine prevents alterations of intestinal microcirculation that are induced by surgical stress and pain in a novel rat model.** Anesth Analg 2012, **115**(1):46-53.

41. Yin Y, Yan M, Zhu T: **Minimum alveolar concentration of sevoflurane in rabbits with liver fibrosis.** Anesth Analg 2012, **114**(3):561-565.

42. Zhang LN, Li ZJ, Tong L, Guo C, Niu JY, Hou WG, Dong HL: **Orexin-A facilitates emergence from propofol anesthesia in the rat.** Anesth Analg 2012, **115**(4):789-796.

43. Zhang Z, Wang C, Gu G, Li H, Zhao H, Wang K, Han F, Wang G: **The effects of electroacupuncture at the ST36 (Zusanli) acupoint on cancer pain and transient receptor potential vanilloid subfamily 1 expression in Walker 256 tumor-bearing rats.** Anesth Analg 2012, **114**(4):879-885.

44. Zurek AA, Bridgwater EM, Orser BA: **Inhibition of alpha5 gamma-Aminobutyric acid type A receptors restores recognition memory after general anesthesia.** Anesth Analg 2012, **114**(4):845-855.

**7. European Journal of Pain**

1. Andrews N, Legg E, Lisak D, Issop Y, Richardson D, Harper S, Pheby T, Huang W, Burgess G, Machin I, Rice AS: **Spontaneous burrowing behaviour in the rat is reduced by peripheral nerve injury or inflammation associated pain.** Eur J Pain 2012, **16**(4):485-495.

2. Benedetti M, Merino R, Kusuda R, Ravanelli MI, Cadetti F, dos Santos P, Zanon S, Lucas G: **Plasma corticosterone levels in mouse models of pain.** Eur J Pain 2012, **16**(6):803-815.

3. Blackbeard J, Wallace VC, O'Dea KP, Hasnie F, Segerdahl A, Pheby T, Field MJ, Takata M, Rice AS: **The correlation between pain-related behaviour and spinal microgliosis in four distinct models of peripheral neuropathy.** Eur J Pain 2012, **16**(10):1357-1367.

4. Brenchat A, Zamanillo D, Hamon M, Romero L, Vela JM: **Role of peripheral versus spinal 5-HT(7) receptors in the modulation of pain undersensitizing conditions.** Eur J Pain 2012, **16**(1):72-81.

5. Brissett DI, Whistler JL, van Rijn RM: **Contribution of mu and delta opioid receptors to the pharmacological profile of kappa opioid receptor subtypes.** Eur J Pain 2012, **16**(3):327-337.

6. Chung C, Carteret AF, McKelvy AD, Ringkamp M, Yang F, Hartke TV, Dong X, Raja SN, Guan Y: **Analgesic properties of loperamide differ following systemic and local administration to rats after spinal nerve injury.** Eur J Pain 2012, **16**(7):1021-1032.

7. Dominguez CA, Strom M, Gao T, Zhang L, Olsson T, Wiesenfeld-Hallin Z, Xu XJ, Piehl F: **Genetic and sex influence on neuropathic pain-like behaviour after spinal cord injury in the rat.** Eur J Pain 2012, **16**(10):1368-1377.

8. Escobar W, Ramirez K, Avila C, Limongi R, Vanegas H, Vazquez E: **Metamizol, a non-opioid analgesic, acts via endocannabinoids in the PAG-RVM axis during inflammation in rats.** Eur J Pain 2012, **16**(5):676-689.

9. Fais RS, Reis GM, Silveira JW, Dias QM, Rossaneis AC, Prado WA: **Amitriptyline prolongs the antihyperalgesic effect of 2- or 100-Hz electro-acupuncture in a rat model of post-incision pain.** Eur J Pain 2012, **16**(5):666-675.

10. Favaro-Moreira NC, Parada CA, Tambeli CH: **Blockade of beta(1)-, beta(2)- and beta(3)-adrenoceptors in the temporomandibular joint induces antinociception especially in female rats.** Eur J Pain 2012, **16**(9):1302-1310.

11. Gu N, Niu JY, Liu WT, Sun YY, Liu S, Lv Y, Dong HL, Song XJ, Xiong LZ: **Hyperbaric oxygen therapy attenuates neuropathic hyperalgesia in rats and idiopathic trigeminal neuralgia in patients.** Eur J Pain 2012, **16**(8):1094-1105.

12. Guillemette A, Dansereau MA, Beaudet N, Richelson E, Sarret P: **Intrathecal administration of NTS1 agonists reverses nociceptive behaviors in a rat model of neuropathic pain.** Eur J Pain 2012, **16**(4):473-484.

13. Jasper LL, MacNeil BJ: **Diverse sensory inputs permit priming in the acidic saline model of hyperalgesia.** Eur J Pain 2012, **16**(7):966-973.

14. Kawasaki-Yatsugi S, Nagakura Y, Ogino S, Sekizawa T, Kiso T, Takahashi M, Ishikawa G, Ito H, Shimizu Y: **Automated measurement of spontaneous pain-associated limb movement and drug efficacy evaluation in a rat model of neuropathic pain.** Eur J Pain 2012, **16**(10):1426-1436.

15. Khalefa BI, Shaqura M, Al-Khrasani M, Furst S, Mousa SA, Schafer M: **Relative contributions of peripheral versus supraspinal or spinal opioid receptors to the antinociception of systemic opioids.** Eur J Pain 2012, **16**(5):690-705.

16. Klein MM, Lee JW, Siegel SM, Downs HM, Oaklander AL: **Endoneurial pathology of the needlestick-nerve-injury model of Complex Regional Pain Syndrome, including rats with and without pain behaviors.** Eur J Pain 2012, **16**(1):28-37.

17. Ma MT, Yeo JF, Shui G, Wenk MR, Ong WY: **Systems wide analyses of lipids in the brainstem during inflammatory orofacial pain - evidence of increased phospholipase A(2) activity.** Eur J Pain 2012, **16**(1):38-48.

18. Ohmichi Y, Sato J, Ohmichi M, Sakurai H, Yoshimoto T, Morimoto A, Hashimoto T, Eguchi K, Nishihara M, Arai YC, Ohishi H, Asamoto K, Ushida T, Nakano T, Kumazawa T: **Two-week cast immobilization induced chronic widespread hyperalgesia in rats.** Eur J Pain 2012, **16**(3):338-348.

19. Quinteiro MS, Napimoga MH, Mesquita KP, Clemente-Napimoga JT: **The indirect antinociceptive mechanism of 15d-PGJ2 on rheumatoid arthritis-induced TMJ inflammatory pain in rats.** Eur J Pain 2012, **16**(8):1106-1115.

20. Rouwette T, Vanelderen P, de Reus M, Loohuis NO, Giele J, van Egmond J, Scheenen W, Scheffer GJ, Roubos E, Vissers K, Kozicz T: **Experimental neuropathy increases limbic forebrain CRF.** Eur J Pain 2012, **16**(1):61-71.

21. Saika F, Kiguchi N, Kobayashi Y, Fukazawa Y, Kishioka S: **CC-chemokine ligand 4/macrophage inflammatory protein-1beta participates in the induction of neuropathic pain after peripheral nerve injury.** Eur J Pain 2012, **16**(9):1271-1280.

22. Samengo I, Curro D, Navarra P, Barrese V, Taglialatela M, Martire M: **Molecular and pharmacological evidence for a facilitatory functional role of pre-synaptic GLUK2/3 kainate receptors on GABA release in rat trigeminal caudal nucleus.** Eur J Pain 2012, **16**(8):1148-1157.

23. Sanchez Robles EM, Bagues Arias A, Martin Fontelles MI: **Cannabinoids and muscular pain. Effectiveness of the local administration in rat.** Eur J Pain 2012, **16**(8):1116-1127.

24. Su TF, Zhao YQ, Zhang LH, Peng M, Wu CH, Pei L, Tian B, Zhang J, Shi J, Pan HL, Li M: **Electroacupuncture reduces the expression of proinflammatory cytokines in inflamed skin tissues through activation of cannabinoid CB2 receptors.** Eur J Pain 2012, **16**(5):624-635.

25. Sun T, Yu E, Yu L, Luo J, Li H, Fu Z: **LipoxinA(4) induced antinociception and decreased expression of NF-kappaB and pro-inflammatory cytokines after chronic dorsal root ganglia compression in rats.** Eur J Pain 2012, **16**(1):18-27.

26. Torres-Chavez KE, Sanfins JM, Clemente-Napimoga JT, Pelegrini-Da-Silva A, Parada CA, Fischer L, Tambeli CH: **Effect of gonadal steroid hormones on formalin-induced temporomandibular joint inflammation.** Eur J Pain 2012, **16**(2):204-216.

27. Tsang SW, Zhao M, Wu J, Sung JJ, Bian ZX: **Nerve growth factor-mediated neuronal plasticity in spinal cord contributes to neonatal maternal separation-induced visceral hypersensitivity in rats.** Eur J Pain 2012, **16**(4):463-472.

28. Wu JX, Xu MY, Miao XR, Lu ZJ, Yuan XM, Li XQ, Yu WF: **Functional up-regulation of P2X3 receptors in dorsal root ganglion in a rat model of bone cancer pain.** Eur J Pain 2012, **16**(10):1378-1388.

29. Yasui M, Shiraishi Y, Ozaki N, Hayashi K, Hori K, Ichiyanagi M, Sugiura Y: **Nerve growth factor and associated nerve sprouting contribute to local mechanical hyperalgesia in a rat model of bone injury.** Eur J Pain 2012, **16**(7):953-965.

30. Zhang Y, Meng X, Li A, Xin J, Berman BM, Lao L, Tan M, Ren K, Zhang RX: **Electroacupuncture alleviates affective pain in an inflammatory pain rat model.** Eur J Pain 2012, **16**(2):170-181.

**8. Minerva Anestesiologica**

No eligible manuscripts were identified.

**9. European Journal of Anaesthesiology**

1. Callaway JK, Jones NC, Royse CF: **Isoflurane induces cognitive deficits in the Morris water maze task in rats.** Eur J Anaesthesiol 2012, **29**(5):239-245.

2. Kamer AR, Galoyan SM, Haile M, Kline R, Boutajangout A, Li YS, Bekker A: **Meloxicam improves object recognition memory and modulates glial activation after splenectomy in mice.** Eur J Anaesthesiol 2012, **29**(7):332-337.

3. Nakatani H, Kim C, Sakamoto A: **Low-dose dexmedetomidine facilitates the carotid body response to low oxygen tension in vitro via alpha2-adrenergic receptor activation in rabbits.** Eur J Anaesthesiol 2012, **29**(12):570-576.

4. Su CS, Lai HC, Lee WL, Ting CT, Yang YL, Lee HW, Wang LC, Peng CY, Wang KY, Liu TJ: **A secure and rapid method for orotracheal intubation of laboratory rats utilising handy instruments.** Eur J Anaesthesiol 2012, **29**(11):515-519.

**10. Pain Practice**

No eligible manuscripts were identified.

**II. Category: Critical Care Medicine**

**11. American Journal of Respiratory and Critical Care Medicine**

1. Adamali H, Armstrong ME, McLaughlin AM, Cooke G, McKone E, Costello CM, Gallagher CG, Leng L, Baugh JA, Fingerle-Rowson G, Bucala RJ, McLoughlin P, Donnelly SC: **Macrophage migration inhibitory factor enzymatic activity, lung inflammation, and cystic fibrosis.** Am J Respir Crit Care Med 2012, **186**(2):162-169.

2. Aono Y, Ledford JG, Mukherjee S, Ogawa H, Nishioka Y, Sone S, Beers MF, Noble PW, Wright JR: **Surfactant protein-D regulates effector cell function and fibrotic lung remodeling in response to bleomycin injury.** Am J Respir Crit Care Med 2012, **185**(5):525-536.

3. Apostolou E, Stavropoulos A, Sountoulidis A, Xirakia C, Giaglis S, Protopapadakis E, Ritis K, Mentzelopoulos S, Pasternack A, Foster M, Ritvos O, Tzelepis GE, Andreakos E, Sideras P: **Activin-A overexpression in the murine lung causes pathology that simulates acute respiratory distress syndrome.** Am J Respir Crit Care Med 2012, **185**(4):382-391.

4. Barletta KE, Cagnina RE, Burdick MD, Linden J, Mehrad B: **Adenosine A(2B) receptor deficiency promotes host defenses against gram-negative bacterial pneumonia.** Am J Respir Crit Care Med 2012, **186**(10):1044-1050.

5. Ben J, Jin G, Zhang Y, Ma B, Bai H, Chen J, Zhang H, Gong Q, Zhou X, Zhang H, Qian L, Zhu X, Li X, Yang Q, Hu Z, Xu Y, Shen H, Chen Q: **Class A scavenger receptor deficiency exacerbates lung tumorigenesis by cultivating a procarcinogenic microenvironment in humans and mice.** Am J Respir Crit Care Med 2012, **186**(8):763-772.

6. Chaudhary N, Datta K, Askin FB, Staab JF, Marr KA: **Cystic fibrosis transmembrane conductance regulator regulates epithelial cell response to Aspergillus and resultant pulmonary inflammation.** Am J Respir Crit Care Med 2012, **185**(3):301-310.

7. Churg A, Marshall CV, Sin DD, Bolton S, Zhou S, Thain K, Cadogan EB, Maltby J, Soars MG, Mallinder PR, Wright JL: **Late intervention with a myeloperoxidase inhibitor stops progression of experimental chronic obstructive pulmonary disease.** Am J Respir Crit Care Med 2012, **185**(1):34-43.

8. Dallas ML, Yang Z, Boyle JP, Boycott HE, Scragg JL, Milligan CJ, Elies J, Duke A, Thireau J, Reboul C, Richard S, Bernus O, Steele DS, Peers C: **Carbon monoxide induces cardiac arrhythmia via induction of the late Na+ current.** Am J Respir Crit Care Med 2012, **186**(7):648-656.

9. Dhaliwal K, Scholefield E, Ferenbach D, Gibbons M, Duffin R, Dorward DA, Morris AC, Humphries D, MacKinnon A, Wilkinson TS, Wallace WA, van Rooijen N, Mack M, Rossi AG, Davidson DJ, Hirani N, Hughes J, Haslett C, Simpson AJ: **Monocytes control second-phase neutrophil emigration in established lipopolysaccharide-induced murine lung injury.** Am J Respir Crit Care Med 2012, **186**(6):514-524.

10. Dolinay T, Kim YS, Howrylak J, Hunninghake GM, An CH, Fredenburgh L, Massaro AF, Rogers A, Gazourian L, Nakahira K, Haspel JA, Landazury R, Eppanapally S, Christie JD, Meyer NJ, Ware LB, Christiani DC, Ryter SW, Baron RM, Choi AM: **Inflammasome-regulated cytokines are critical mediators of acute lung injury.** Am J Respir Crit Care Med 2012, **185**(11):1225-1234.

11. Files DC, D'Alessio FR, Johnston LF, Kesari P, Aggarwal NR, Garibaldi BT, Mock JR, Simmers JL, DeGorordo A, Murdoch J, Willis MS, Patterson C, Tankersley CG, Messi ML, Liu C, Delbono O, Furlow JD, Bodine SC, Cohn RD, King LS, Crow MT: **A critical role for muscle ring finger-1 in acute lung injury-associated skeletal muscle wasting.** Am J Respir Crit Care Med 2012, **185**(8):825-834.

12. Griesenbach U, Inoue M, Meng C, Farley R, Chan M, Newman NK, Brum A, You J, Kerton A, Shoemark A, Boyd AC, Davies JC, Higgins TE, Gill DR, Hyde SC, Innes JA, Porteous DJ, Hasegawa M, Alton EW: **Assessment of F/HN-pseudotyped lentivirus as a clinically relevant vector for lung gene therapy.** Am J Respir Crit Care Med 2012, **186**(9):846-856.

13. Grommes J, Alard JE, Drechsler M, Wantha S, Morgelin M, Kuebler WM, Jacobs M, von Hundelshausen P, Markart P, Wygrecka M, Preissner KT, Hackeng TM, Koenen RR, Weber C, Soehnlein O: **Disruption of platelet-derived chemokine heteromers prevents neutrophil extravasation in acute lung injury.** Am J Respir Crit Care Med 2012, **185**(6):628-636.

14. Herzig DS, Driver BR, Fang G, Toliver-Kinsky TE, Shute EN, Sherwood ER: **Regulation of lymphocyte trafficking by CXC chemokine receptor 3 during septic shock.** Am J Respir Crit Care Med 2012, **185**(3):291-300.

15. Kang MJ, Choi JM, Kim BH, Lee CM, Cho WK, Choe G, Kim DH, Lee CG, Elias JA: **IL-18 induces emphysema and airway and vascular remodeling via IFN-gamma, IL-17A, and IL-13.** Am J Respir Crit Care Med 2012, **185**(11):1205-1217.

16. Lahm T, Albrecht M, Fisher AJ, Selej M, Patel NG, Brown JA, Justice MJ, Brown MB, Van Demark M, Trulock KM, Dieudonne D, Reddy JG, Presson RG, Petrache I: **17beta-Estradiol attenuates hypoxic pulmonary hypertension via estrogen receptor-mediated effects.** Am J Respir Crit Care Med 2012, **185**(9):965-980.

17. Linch SN, Danielson ET, Kelly AM, Tamakawa RA, Lee JJ, Gold JA: **Interleukin 5 is protective during sepsis in an eosinophil-independent manner.** Am J Respir Crit Care Med 2012, **186**(3):246-254.

18. MacGarvey NC, Suliman HB, Bartz RR, Fu P, Withers CM, Welty-Wolf KE, Piantadosi CA: **Activation of mitochondrial biogenesis by heme oxygenase-1-mediated NF-E2-related factor-2 induction rescues mice from lethal Staphylococcus aureus sepsis.** Am J Respir Crit Care Med 2012, **185**(8):851-861.

19. Mackinnon AC, Gibbons MA, Farnworth SL, Leffler H, Nilsson UJ, Delaine T, Simpson AJ, Forbes SJ, Hirani N, Gauldie J, Sethi T: **Regulation of transforming growth factor-beta1-driven lung fibrosis by galectin-3.** Am J Respir Crit Care Med 2012, **185**(5):537-546.

20. Marsboom G, Wietholt C, Haney CR, Toth PT, Ryan JJ, Morrow E, Thenappan T, Bache-Wiig P, Piao L, Paul J, Chen CT, Archer SL: **Lung (1)(8)F-fluorodeoxyglucose positron emission tomography for diagnosis and monitoring of pulmonary arterial hypertension.** Am J Respir Crit Care Med 2012, **185**(6):670-679.

21. Park MS, He Q, Edwards MG, Sergew A, Riches DW, Albert RK, Douglas IS: **Mitogen-activated protein kinase phosphatase-1 modulates regional effects of injurious mechanical ventilation in rodent lungs.** Am J Respir Crit Care Med 2012, **186**(1):72-81.

22. Pullamsetti SS, Doebele C, Fischer A, Savai R, Kojonazarov B, Dahal BK, Ghofrani HA, Weissmann N, Grimminger F, Bonauer A, Seeger W, Zeiher AM, Dimmeler S, Schermuly RT: **Inhibition of microRNA-17 improves lung and heart function in experimental pulmonary hypertension.** Am J Respir Crit Care Med 2012, **185**(4):409-419.

23. Samapati R, Yang Y, Yin J, Stoerger C, Arenz C, Dietrich A, Gudermann T, Adam D, Wu S, Freichel M, Flockerzi V, Uhlig S, Kuebler WM: **Lung endothelial Ca2+ and permeability response to platelet-activating factor is mediated by acid sphingomyelinase and transient receptor potential classical 6.** Am J Respir Crit Care Med 2012, **185**(2):160-170.

24. Shi Y, Cao J, Gao J, Zheng L, Goodwin A, An CH, Patel A, Lee JS, Duncan SR, Kaminski N, Pandit KV, Rosas IO, Choi AM, Morse D: **Retinoic acid-related orphan receptor-alpha is induced in the setting of DNA damage and promotes pulmonary emphysema.** Am J Respir Crit Care Med 2012, **186**(5):412-419.

25. Su G, Atakilit A, Li JT, Wu N, Bhattacharya M, Zhu J, Shieh JE, Li E, Chen R, Sun S, Su CP, Sheppard D: **Absence of integrin alphavbeta3 enhances vascular leak in mice by inhibiting endothelial cortical actin formation.** Am J Respir Crit Care Med 2012, **185**(1):58-66.

26. Tsujino K, Takeda Y, Arai T, Shintani Y, Inagaki R, Saiga H, Iwasaki T, Tetsumoto S, Jin Y, Ihara S, Minami T, Suzuki M, Nagatomo I, Inoue K, Kida H, Kijima T, Ito M, Kitaichi M, Inoue Y, Tachibana I, Takeda K, Okumura M, Hemler ME, Kumanogoh A: **Tetraspanin CD151 protects against pulmonary fibrosis by maintaining epithelial integrity.** Am J Respir Crit Care Med 2012, **186**(2):170-180.

27. Tu L, De Man FS, Girerd B, Huertas A, Chaumais MC, Lecerf F, Francois C, Perros F, Dorfmuller P, Fadel E, Montani D, Eddahibi S, Humbert M, Guignabert C: **A critical role for p130Cas in the progression of pulmonary hypertension in humans and rodents.** Am J Respir Crit Care Med 2012, **186**(7):666-676.

28. Vadivel A, van Haaften T, Alphonse RS, Rey-Parra GJ, Ionescu L, Haromy A, Eaton F, Michelakis E, Thebaud B: **Critical role of the axonal guidance cue EphrinB2 in lung growth, angiogenesis, and repair.** Am J Respir Crit Care Med 2012, **185**(5):564-574.

29. Wang L, Zhao L, Lv J, Yin Q, Liang X, Chu Y, He R: **BLT1-dependent alveolar recruitment of CD4(+)CD25(+) Foxp3(+) regulatory T cells is important for resolution of acute lung injury.** Am J Respir Crit Care Med 2012, **186**(10):989-998.

30. Wu W, Huang J, Duan B, Traficante DC, Hong H, Risech M, Lory S, Priebe GP: **Th17-stimulating protein vaccines confer protection against Pseudomonas aeruginosa pneumonia.** Am J Respir Crit Care Med 2012, **186**(5):420-427.

31. Young LR, Gulleman PM, Bridges JP, Weaver TE, Deutsch GH, Blackwell TS, McCormack FX: **The alveolar epithelium determines susceptibility to lung fibrosis in Hermansky-Pudlak syndrome.** Am J Respir Crit Care Med 2012, **186**(10):1014-1024.

32. Yu G, Kovkarova-Naumovski E, Jara P, Parwani A, Kass D, Ruiz V, Lopez-Otin C, Rosas IO, Gibson KF, Cabrera S, Ramirez R, Yousem SA, Richards TJ, Chensny LJ, Selman M, Kaminski N, Pardo A: **Matrix metalloproteinase-19 is a key regulator of lung fibrosis in mice and humans.** Am J Respir Crit Care Med 2012, **186**(8):752-762.

33. Zafrani L, Gerotziafas G, Byrnes C, Hu X, Perez J, Levi C, Placier S, Letavernier E, Leelahavanichkul A, Haymann JP, Elalamy I, Miller JL, Star RA, Yuen PS, Baud L: **Calpastatin controls polymicrobial sepsis by limiting procoagulant microparticle release.** Am J Respir Crit Care Med 2012, **185**(7):744-755.

**12. Critical Care Medicine**

1. Agten A, Maes K, Thomas D, Cielen N, Van Hees HW, Dekhuijzen RP, Decramer M, Gayan-Ramirez G: **Bortezomib partially protects the rat diaphragm from ventilator-induced diaphragm dysfunction.** Crit Care Med 2012, **40**(8):2449-2455.

2. Alhaddad H, Cisternino S, Decleves X, Tournier N, Schlatter J, Chiadmi F, Risede P, Smirnova M, Besengez C, Scherrmann JM, Baud FJ, Megarbane B: **Respiratory toxicity of buprenorphine results from the blockage of P-glycoprotein-mediated efflux of norbuprenorphine at the blood-brain barrier in mice.** Crit Care Med 2012, **40**(12):3215-3223.

3. Altay O, Hasegawa Y, Sherchan P, Suzuki H, Khatibi NH, Tang J, Zhang JH: **Isoflurane delays the development of early brain injury after subarachnoid hemorrhage through sphingosine-related pathway activation in mice.** Crit Care Med 2012, **40**(6):1908-1913.

4. Benavides U, Gonzalez-Murguiondo M, Harii N, Lewis CJ, Schwartz AL, Giuliani C, Napolitano G, Dagia NM, Malgor R, McCall KD, Kohn LD: **Phenylmethimazole inhibits production of proinflammatory mediators and is protective in an experimental model of endotoxic shock*.** Crit Care Med 2012, **40**(3):886-894.

5. Beurskens CJ, Aslami H, Kuipers MT, Horn J, Vroom MB, van Kuilenburg AB, Roelofs JJ, Schultz MJ, Juffermans NP: **Induced hypothermia is protective in a rat model of pneumococcal pneumonia associated with increased adenosine triphosphate availability and turnover*.** Crit Care Med 2012, **40**(3):919-926.

6. Bitto A, Polito F, Irrera N, Calo M, Spaccapelo L, Marini HR, Giuliani D, Ottani A, Rinaldi M, Minutoli L, Guarini S, Squadrito F, Altavilla D: **Protective effects of melanocortins on short-term changes in a rat model of traumatic brain injury*.** Crit Care Med 2012, **40**(3):945-951.

7. Breuillard C, Darquy S, Curis E, Neveux N, Garnier JP, Cynober L, De Bandt JP: **Effects of a diabetes-specific enteral nutrition on nutritional and immune status of diabetic, obese, and endotoxemic rats: interest of a graded arginine supply.** Crit Care Med 2012, **40**(8):2423-2430.

8. Cabrera-Benitez NE, Parotto M, Post M, Han B, Spieth PM, Cheng WE, Valladares F, Villar J, Liu M, Sato M, Zhang H, Slutsky AS: **Mechanical stress induces lung fibrosis by epithelial-mesenchymal transition.** Crit Care Med 2012, **40**(2):510-517.

9. Chan YL, Orie NN, Dyson A, Taylor V, Stidwill RP, Clapp LH, Singer M: **Inhibition of vascular adenosine triphosphate-sensitive potassium channels by sympathetic tone during sepsis.** Crit Care Med 2012, **40**(4):1261-1268.

10. Chen TY, Lin MH, Lee WT, Huang SY, Chen YH, Lee AC, Lin HW, Lee EJ: **Nicotinamide inhibits nuclear factor-kappa B translocation after transient focal cerebral ischemia.** Crit Care Med 2012, **40**(2):532-537.

11. Chien MH, Bien MY, Ku CC, Chang YC, Pao HY, Yang YL, Hsiao M, Chen CL, Ho JH: **Systemic human orbital fat-derived stem/stromal cell transplantation ameliorates acute inflammation in lipopolysaccharide-induced acute lung injury.** Crit Care Med 2012, **40**(4):1245-1253.

12. Chierichetti M, Engelberts D, El-Khuffash A, Babyn P, Post M, Kavanagh BP: **Continuous negative abdominal distension augments recruitment of atelectatic lung.** Crit Care Med 2012, **40**(6):1864-1872.

13. Contreras M, Ansari B, Curley G, Higgins BD, Hassett P, O'Toole D, Laffey JG: **Hypercapnic acidosis attenuates ventilation-induced lung injury by a nuclear factor-kappaB-dependent mechanism.** Crit Care Med 2012, **40**(9):2622-2630.

14. David S, Mukherjee A, Ghosh CC, Yano M, Khankin EV, Wenger JB, Karumanchi SA, Shapiro NI, Parikh SM: **Angiopoietin-2 may contribute to multiple organ dysfunction and death in sepsis*.** Crit Care Med 2012, **40**(11):3034-3041.

15. Davis RT,3rd, Bruells CS, Stabley JN, McCullough DJ, Powers SK, Behnke BJ: **Mechanical ventilation reduces rat diaphragm blood flow and impairs oxygen delivery and uptake.** Crit Care Med 2012, **40**(10):2858-2866.

16. Dong C, Rovnaghi CR, Anand KJ: **Ketamine alters the neurogenesis of rat cortical neural stem progenitor cells.** Crit Care Med 2012, **40**(8):2407-2416.

17. Empey PE, Miller TM, Philbrick AH, Melick JA, Kochanek PM, Poloyac SM: **Mild hypothermia decreases fentanyl and midazolam steady-state clearance in a rat model of cardiac arrest.** Crit Care Med 2012, **40**(4):1221-1228.

18. Esen F, Senturk E, Ozcan PE, Ahishali B, Arican N, Orhan N, Ekizoglu O, Kucuk M, Kaya M: **Intravenous immunoglobulins prevent the breakdown of the blood-brain barrier in experimentally induced sepsis.** Crit Care Med 2012, **40**(4):1214-1220.

19. Gao M, Ha T, Zhang X, Liu L, Wang X, Kelley J, Singh K, Kao R, Gao X, Williams D, Li C: **Toll-like receptor 3 plays a central role in cardiac dysfunction during polymicrobial sepsis.** Crit Care Med 2012, **40**(8):2390-2399.

20. Heckel K, Winkelmann B, Strunden MS, Basedow A, Schuster A, Schumacher U, Kiefmann R, Reuter DA, Goetz AE: **Tetrastarch sustains pulmonary microvascular perfusion and gas exchange during systemic inflammation.** Crit Care Med 2012, **40**(2):518-531.

21. Heming N, Letteron P, Driss F, Millot S, El Benna J, Tourret J, Denamur E, Montravers P, Beaumont C, Lasocki S: **Efficacy and toxicity of intravenous iron in a mouse model of critical care anemia*.** Crit Care Med 2012, **40**(7):2141-2148.

22. Hu H, Xenocostas A, Chin-Yee N, Lu X, Chin-Yee I, Feng Q: **Transfusion of fresh but not old stored blood reduces infarct size and improves cardiac function after acute myocardial infarction in anemic rats*.** Crit Care Med 2012, **40**(3):740-746.

23. Hu R, Sun H, Zhang Q, Chen J, Wu N, Meng H, Cui G, Hu S, Li F, Lin J, Wan Q, Feng H: **G-protein coupled estrogen receptor 1 mediated estrogenic neuroprotection against spinal cord injury.** Crit Care Med 2012, **40**(12):3230-3237.

24. Hudson MB, Smuder AJ, Nelson WB, Bruells CS, Levine S, Powers SK: **Both high level pressure support ventilation and controlled mechanical ventilation induce diaphragm dysfunction and atrophy.** Crit Care Med 2012, **40**(4):1254-1260.

25. Kager LM, Wiersinga WJ, Roelofs JJ, Meijers JC, Levi M, Van't Veer C, van der Poll T: **Endogenous tissue-type plasminogen activator impairs host defense during severe experimental Gram-negative sepsis (melioidosis)*.** Crit Care Med 2012, **40**(7):2168-2175.

26. Kuper C, Fraek ML, Muller HH, Beck FX, Neuhofer W: **Sepsis-induced urinary concentration defect is related to nitric oxide-dependent inactivation of TonEBP/NFAT5, which downregulates renal medullary solute transport proteins and aquaporin-2.** Crit Care Med 2012, **40**(6):1887-1895.

27. Lee BF, Wang LW, Lin SH, Jhuo TJ, Chiu NT, Huang CC, Hsia CC, Shen LH: **Tc-99m-HL91 imaging in the early detection of neuronal injury in a neonatal rat model of hypoxic ischemia.** Crit Care Med 2012, **40**(6):1930-1938.

28. Lee SY, Lee YS, Choi HM, Ko YS, Lee HY, Jo SK, Cho WY, Kim HK: **Distinct pathophysiologic mechanisms of septic acute kidney injury: role of immune suppression and renal tubular cell apoptosis in murine model of septic acute kidney injury.** Crit Care Med 2012, **40**(11):2997-3006.

29. Leon K, Pichavant-Rafini K, Quemener E, Sebert P, Egreteau PY, Ollivier H, Carre JL, L'Her E: **Oxygen blood transport during experimental sepsis: effect of hypothermia*.** Crit Care Med 2012, **40**(3):912-918.

30. Letson HL, Pecheniuk NM, Mhango LP, Dobson GP: **Reversal of acute coagulopathy during hypotensive resuscitation using small-volume 7.5% NaCl adenocaine and Mg2+ in the rat model of severe hemorrhagic shock.** Crit Care Med 2012, **40**(8):2417-2422.

31. Liu MS, Liu CH, Wu G, Zhou Y: **Antisense inhibition of secretory and cytosolic phospholipase A2 reduces the mortality in rats with sepsis*.** Crit Care Med 2012, **40**(7):2132-2140.

32. Maddens B, Vandendriessche B, Demon D, Vanholder R, Chiers K, Cauwels A, Meyer E: **Severity of sepsis-induced acute kidney injury in a novel mouse model is age dependent.** Crit Care Med 2012, **40**(9):2638-2646.

33. Maybhate A, Hu C, Bazley FA, Yu Q, Thakor NV, Kerr CL, All AH: **Potential long-term benefits of acute hypothermia after spinal cord injury: assessments with somatosensory-evoked potentials.** Crit Care Med 2012, **40**(2):573-579.

34. Nelson WB, Smuder AJ, Hudson MB, Talbert EE, Powers SK: **Cross-talk between the calpain and caspase-3 proteolytic systems in the diaphragm during prolonged mechanical ventilation.** Crit Care Med 2012, **40**(6):1857-1863.

35. Noppens RR, Kelm RF, Lindemann R, Engelhard K, Werner C, Kempski O: **Effects of a single-dose hypertonic saline hydroxyethyl starch on cerebral blood flow, long-term outcome, neurogenesis, and neuronal survival after cardiac arrest and cardiopulmonary resuscitation in rats*.** Crit Care Med 2012, **40**(7):2149-2156.

36. Partownavid P, Umar S, Li J, Rahman S, Eghbali M: **Fatty-acid oxidation and calcium homeostasis are involved in the rescue of bupivacaine-induced cardiotoxicity by lipid emulsion in rats.** Crit Care Med 2012, **40**(8):2431-2437.

37. Pecchiari M, Monaco A, Koutsoukou A, D'Angelo E: **Plasma membrane disruptions with different modes of injurious mechanical ventilation in normal rat lungs*.** Crit Care Med 2012, **40**(3):869-875.

38. Pejo E, Feng Y, Chao W, Cotten JF, Le Ge R, Raines DE: **Differential effects of etomidate and its pyrrole analogue carboetomidate on the adrenocortical and cytokine responses to endotoxemia.** Crit Care Med 2012, **40**(1):187-192.

39. Peng ZY, Wang HZ, Srisawat N, Wen X, Rimmele T, Bishop J, Singbartl K, Murugan R, Kellum JA: **Bactericidal antibiotics temporarily increase inflammation and worsen acute kidney injury in experimental sepsis.** Crit Care Med 2012, **40**(2):538-543.

40. Philippart F, Fitting C, Cavaillon JM: **Lung microenvironment contributes to the resistance of alveolar macrophages to develop tolerance to endotoxin*.** Crit Care Med 2012, **40**(11):2987-2996.

41. Sheng C, Yu YH, Zhao KS, Huang LP, Lodato RF, Wang ZJ, Wang CH, Wang SH, Zha DS: **Acute lung inflammatory response and injury after hemorrhagic shock are more severe in postpartum rabbits.** Crit Care Med 2012, **40**(5):1570-1577.

42. Smeding L, Leong-Poi H, Hu P, Shan Y, Haitsma JJ, Horvath E, Furmli S, Masoom H, Kuiper JW, Slutsky AS, Parker TG, Plotz FB, dos Santos CC: **Salutary effect of resveratrol on sepsis-induced myocardial depression.** Crit Care Med 2012, **40**(6):1896-1907.

43. Smuder AJ, Hudson MB, Nelson WB, Kavazis AN, Powers SK: **Nuclear factor-kappaB signaling contributes to mechanical ventilation-induced diaphragm weakness*.** Crit Care Med 2012, **40**(3):927-934.

44. Sun CK, Chang CL, Lin YC, Kao YH, Chang LT, Yen CH, Shao PL, Chen CH, Leu S, Yip HK: **Systemic administration of autologous adipose-derived mesenchymal stem cells alleviates hepatic ischemia-reperfusion injury in rats.** Crit Care Med 2012, **40**(4):1279-1290.

45. Timaru-Kast R, Wyschkon S, Luh C, Schaible EV, Lehmann F, Merk P, Werner C, Engelhard K, Thal SC: **Delayed inhibition of angiotensin II receptor type 1 reduces secondary brain damage and improves functional recovery after experimental brain trauma*.** Crit Care Med 2012, **40**(3):935-944.

46. Trevelin SC, Alves-Filho JC, Sonego F, Turato W, Nascimento DC, Souto FO, Cunha TM, Gazzinelli RT, Cunha FQ: **Toll-like receptor 9 activation in neutrophils impairs chemotaxis and reduces sepsis outcome.** Crit Care Med 2012, **40**(9):2631-2637.

47. Wilson MR, Patel BV, Takata M: **Ventilation with "clinically relevant" high tidal volumes does not promote stretch-induced injury in the lungs of healthy mice.** Crit Care Med 2012, **40**(10):2850-2857.

48. Wohlauer M, Moore EE, Silliman CC, Fragoso M, Gamboni F, Harr J, Accurso F, Wright F, Haenel J, Fullerton D, Banerjee A: **Nebulized hypertonic saline attenuates acute lung injury following trauma and hemorrhagic shock via inhibition of matrix metalloproteinase-13.** Crit Care Med 2012, **40**(9):2647-2653.

49. Wu SY, Wu CP, Kang BH, Li MH, Chu SJ, Huang KL: **Hypercapnic acidosis attenuates reperfusion injury in isolated and perfused rat lungs.** Crit Care Med 2012, **40**(2):553-559.

50. Xuan W, Wu B, Chen C, Chen B, Zhang W, Xu D, Bin J, Liao Y: **Resveratrol improves myocardial ischemia and ischemic heart failure in mice by antagonizing the detrimental effects of fractalkine*.** Crit Care Med 2012, **40**(11):3026-3033.

51. Yabluchanskiy A, Sawle P, Homer-Vanniasinkam S, Green CJ, Foresti R, Motterlini R: **CORM-3, a carbon monoxide-releasing molecule, alters the inflammatory response and reduces brain damage in a rat model of hemorrhagic stroke.** Crit Care Med 2012, **40**(2):544-552.

52. Ye R, Yang Q, Kong X, Li N, Zhang Y, Han J, Xiong L, Liu X, Zhao G: **Sevoflurane preconditioning improves mitochondrial function and long-term neurologic sequelae after transient cerebral ischemia: role of mitochondrial permeability transition.** Crit Care Med 2012, **40**(9):2685-2693.

53. Yeh KH, Sheu JJ, Lin YC, Sun CK, Chang LT, Kao YH, Yen CH, Shao PL, Tsai TH, Chen YL, Chua S, Leu S, Yip HK: **Benefit of combined extracorporeal shock wave and bone marrow-derived endothelial progenitor cells in protection against critical limb ischemia in rats.** Crit Care Med 2012, **40**(1):169-177.

54. Yoshida T, Uchiyama A, Matsuura N, Mashimo T, Fujino Y: **Spontaneous breathing during lung-protective ventilation in an experimental acute lung injury model: high transpulmonary pressure associated with strong spontaneous breathing effort may worsen lung injury.** Crit Care Med 2012, **40**(5):1578-1585.

55. Zhan Y, Chen C, Suzuki H, Hu Q, Zhi X, Zhang JH: **Hydrogen gas ameliorates oxidative stress in early brain injury after subarachnoid hemorrhage in rats.** Crit Care Med 2012, **40**(4):1291-1296.

56. Zhou J, Huang WQ, Li C, Wu GY, Li YS, Wen SH, Lei WL, Liu KX: **Intestinal ischemia/reperfusion enhances microglial activation and induces cerebral injury and memory dysfunction in rats.** Crit Care Med 2012, **40**(8):2438-2448.

57. Zhuang L, Yang T, Zhao H, Fidalgo AR, Vizcaychipi MP, Sanders RD, Yu B, Takata M, Johnson MR, Ma D: **The protective profile of argon, helium, and xenon in a model of neonatal asphyxia in rats.** Crit Care Med 2012, **40**(6):1724-1730.

**13. Chest**

1. Bartelds B, van Loon RL, Mohaupt S, Wijnberg H, Dickinson MG, Boersma B, Takens J, van Albada M, Berger RM: **Mast cell inhibition improves pulmonary vascular remodeling in pulmonary hypertension.** Chest 2012, **141**(3):651-660.

2. Liu KS, Liu SJ, Chen HY, Huang YK, Peng YJ, Wu RC, Ueng SW: **Steady antibiotic release from biodegradable beads in the pleural cavity: an in vitro and in vivo study.** Chest 2012, **141**(5):1197-1202.

3. Smith JA, Hilton EC, Saulsberry L, Canning BJ: **Antitussive effects of memantine in guinea pigs.** Chest 2012, **141**(4):996-1002.

4. Tanabe T, Kanoh S, Moskowitz WB, Rubin BK: **Cardiac asthma: transforming growth factor-beta from the failing heart leads to squamous metaplasia in human airway cells and in the murine lung.** Chest 2012, **142**(5):1274-1283.

**14. Intensive Care Medicine**

1. Derive M, Bouazza Y, Alauzet C, Gibot S: **Myeloid-derived suppressor cells control microbial sepsis.** Intensive Care Med 2012, **38**(6):1040-1049.

2. Engelberts D, Malhotra A, Butler JP, Topulos GP, Loring SH, Kavanagh BP: **Relative effects of negative versus positive pressure ventilation depend on applied conditions.** Intensive Care Med 2012, **38**(5):879-885.

3. Finney SJ, Leaver SK, Evans TW, Burke-Gaffney A: **Differences in lipopolysaccharide- and lipoteichoic acid-induced cytokine/chemokine expression.** Intensive Care Med 2012, **38**(2):324-332.

4. Fortis S, Spieth PM, Lu WY, Parotto M, Haitsma JJ, Slutsky AS, Zhong N, Mazer CD, Zhang H: **Effects of anesthetic regimes on inflammatory responses in a rat model of acute lung injury.** Intensive Care Med 2012, **38**(9):1548-1555.

5. Grasselli G, Beck J, Mirabella L, Pesenti A, Slutsky AS, Sinderby C: **Assessment of patient-ventilator breath contribution during neurally adjusted ventilatory assist.** Intensive Care Med 2012, **38**(7):1224-1232.

6. Lourenco AP, Vasques-Novoa F, Oliveira-Pinto J, Fontoura D, Roncon-Albuquerque R,Jr, Leite-Moreira AF: **Haemodynamic and neuroendocrine effects of tezosentan in chronic experimental pulmonary hypertension.** Intensive Care Med 2012, **38**(6):1050-1060.

7. Matsui T, Tasaki M, Yoshioka T, Motoki Y, Tsuneoka H, Nojima J: **Temperature- and time-dependent changes in TLR2-activated microglial NF-kappaB activity and concentrations of inflammatory and anti-inflammatory factors.** Intensive Care Med 2012, **38**(8):1392-1399.

8. Neye N, Enigk F, Shiva S, Habazettl H, Plesnila N, Kuppe H, Gladwin MT, Kuebler WM: **Inhalation of NO during myocardial ischemia reduces infarct size and improves cardiac function.** Intensive Care Med 2012, **38**(8):1381-1391.

9. Oyaizu T, Fung SY, Shiozaki A, Guan Z, Zhang Q, dos Santos CC, Han B, Mura M, Keshavjee S, Liu M: **Src tyrosine kinase inhibition prevents pulmonary ischemia-reperfusion-induced acute lung injury.** Intensive Care Med 2012, **38**(5):894-905.

10. Santos CL, Moraes L, Santos RS, Oliveira MG, Silva JD, Maron-Gutierrez T, Ornellas DS, Morales MM, Capelozzi VL, Jamel N, Pelosi P, Rocco PR, Garcia CS: **Effects of different tidal volumes in pulmonary and extrapulmonary lung injury with or without intraabdominal hypertension.** Intensive Care Med 2012, **38**(3):499-508.

11. Shah KG, Wu R, Jacob A, Molmenti EP, Nicastro J, Coppa GF, Wang P: **Recombinant human milk fat globule-EGF factor 8 produces dose-dependent benefits in sepsis.** Intensive Care Med 2012, **38**(1):128-136.

12. van Hees HW, Schellekens WJ, Andrade Acuna GL, Linkels M, Hafmans T, Ottenheijm CA, Granzier HL, Scheffer GJ, van der Hoeven JG, Dekhuijzen PN, Heunks LM: **Titin and diaphragm dysfunction in mechanically ventilated rats.** Intensive Care Med 2012, **38**(4):702-709.

13. Waisman D, Faingersh A, Levy C, Konyukhov E, Klotzman FI, Rotschild A, Landesberg A: **Early detection of deteriorating ventilation by monitoring bilateral chest wall dynamics in the rabbit.** Intensive Care Med 2012, **38**(1):120-127.

14. Wei X, Duan L, Bai L, Tian M, Li W, Zhang B: **Effects of exogenous hydrogen sulfide on brain metabolism and early neurological function in rabbits after cardiac arrest.** Intensive Care Med 2012, **38**(11):1877-1885.

15. Zambelli V, Di Grigoli G, Scanziani M, Valtorta S, Amigoni M, Belloli S, Messa C, Pesenti A, Fazio F, Bellani G, Moresco RM: **Time course of metabolic activity and cellular infiltration in a murine model of acid-induced lung injury.** Intensive Care Med 2012, **38**(4):694-701.

16. Zlotnik A, Leibowitz A, Gurevich B, Ohayon S, Boyko M, Klein M, Knyazer B, Shapira Y, Teichberg VI: **Effect of estrogens on blood glutamate levels in relation to neurological outcome after TBI in male rats.** Intensive Care Med 2012, **38**(1):137-144.

**15. Critical Care**

1. Alfieri A, Watson JJ, Kammerer RA, Tasab M, Progias P, Reeves K, Brown NJ, Brookes ZL: **Angiopoietin-1 variant reduces LPS-induced microvascular dysfunction in a murine model of sepsis.** Crit Care 2012, **16**(5):R182.

2. Bitto A, Minutoli L, David A, Irrera N, Rinaldi M, Venuti FS, Squadrito F, Altavilla D: **Flavocoxid, a dual inhibitor of COX-2 and 5-LOX of natural origin, attenuates the inflammatory response and protects mice from sepsis.** Crit Care 2012, **16**(1):R32.

3. C C, X Y, Z L, N Z, H H, M W, G J, H S, Z L, S Y: **Hypertonic saline reduces lipopolysaccharide-induced mouse brain edema through inhibiting aquaporin 4 expression.** Crit Care 2012, **16**(5):R186.

4. Calisto KL, Camacho AC, Mittestainer FC, Carvalho BM, Guadagnini D, Carvalheira JB, Saad MJ: **Diacerhein attenuates the inflammatory response and improves survival in a model of severe sepsis.** Crit Care 2012, **16**(4):R158.

5. Chintagari NR, Liu L: **GABA receptor ameliorates ventilator-induced lung injury in rats by improving alveolar fluid clearance.** Crit Care 2012, **16**(2):R55.

6. Herzig DS, Guo Y, Fang G, Toliver-Kinsky TE, Sherwood ER: **Therapeutic efficacy of CXCR3 blockade in an experimental model of severe sepsis.** Crit Care 2012, **16**(5):R168.

7. Lehmann C, Kianian M, Zhou J, Kuster I, Kuschnereit R, Whynot S, Hung O, Shukla R, Johnston B, Cerny V, Pavlovic D, Spassov A, Kelly ME: **Cannabinoid receptor 2 activation reduces intestinal leukocyte recruitment and systemic inflammatory mediator release in acute experimental sepsis.** Crit Care 2012, **16**(2):R47.

8. Meng W, Paunel-Gorgulu A, Flohe S, Hoffmann A, Witte I, Mackenzie C, Baldus SE, Windolf J, Logters TT: **Depletion of neutrophil extracellular traps in vivo results in hypersusceptibility to polymicrobial sepsis in mice.** Crit Care 2012, **16**(4):R137.

9. Reis Goncalves CT, Reis Goncalves CG, de Almeida FM, Dos Santos Lopes FD, Dos Santos Durao AC, Dos Santos FA, da Silva LF, Marcourakis T, Castro-Faria-Neto HC, Vieira RD, Dolhnikoff M: **Protective effects of aerobic exercise on acute lung injury induced by LPS in mice.** Crit Care 2012, **16**(5):R199.

10. Rossaint J, Nadler JL, Ley K, Zarbock A: **Eliminating or blocking 12/15-lipoxygenase reduces neutrophil recruitment in mouse models of acute lung injury.** Crit Care 2012, **16**(5):R166.

11. Schouten M, Van't Veer C, Roelofs JJ, Levi M, van der Poll T: **Protease-activated receptor-1 impairs host defense in murine pneumococcal pneumonia: a controlled laboratory study.** Crit Care 2012, **16**(6):R238.

12. Tsaknis G, Siempos II, Kopterides P, Maniatis NA, Magkou C, Kardara M, Panoutsou S, Kotanidou A, Roussos C, Armaganidis A: **Metformin attenuates ventilator-induced lung injury.** Crit Care 2012, **16**(4):R134.

13. Van de Louw A, Haouzi P: **Oxygen deficit and H2S in hemorrhagic shock in rats.** Crit Care 2012, **16**(5):R178.

14. Yang R, Zou X, Koskinen ML, Tenhunen J: **Ethyl pyruvate reduces liver injury at early phase but impairs regeneration at late phase in acetaminophen overdose.** Crit Care 2012, **16**(1):R9.

15. Yeh YC, Wang MJ, Lin CP, Fan SZ, Tsai JC, Sun WZ, Ko WJ: **Enoxaparin sodium prevents intestinal microcirculatory dysfunction in endotoxemic rats.** Crit Care 2012, **16**(2):R59.

**16. Journal of Neurotrauma**

1. Ahmed AI, Shtaya AB, Zaben MJ, Owens EV, Kiecker C, Gray WP: **Endogenous GFAP-positive neural stem/progenitor cells in the postnatal mouse cortex are activated following traumatic brain injury.** J Neurotrauma 2012, **29**(5):828-842.

2. Ajao DO, Pop V, Kamper JE, Adami A, Rudobeck E, Huang L, Vlkolinsky R, Hartman RE, Ashwal S, Obenaus A, Badaut J: **Traumatic brain injury in young rats leads to progressive behavioral deficits coincident with altered tissue properties in adulthood.** J Neurotrauma 2012, **29**(11):2060-2074.

3. Alant JD, Kemp SW, Khu KJ, Kumar R, Webb AA, Midha R: **Traumatic neuroma in continuity injury model in rodents.** J Neurotrauma 2012, **29**(8):1691-1703.

4. Alessandri B, Schwandt E, Kamada Y, Nagata M, Heimann A, Kempski O: **The neuroprotective effect of lactate is not due to improved glutamate uptake after controlled cortical impact in rats.** J Neurotrauma 2012, **29**(12):2181-2191.

5. Austin JW, Afshar M, Fehlings MG: **The relationship between localized subarachnoid inflammation and parenchymal pathophysiology after spinal cord injury.** J Neurotrauma 2012, **29**(10):1838-1849.

6. Balakathiresan N, Bhomia M, Chandran R, Chavko M, McCarron RM, Maheshwari RK: **MicroRNA let-7i is a promising serum biomarker for blast-induced traumatic brain injury.** J Neurotrauma 2012, **29**(7):1379-1387.

7. Bao F, Omana V, Brown A, Weaver LC: **The systemic inflammatory response after spinal cord injury in the rat is decreased by alpha4beta1 integrin blockade.** J Neurotrauma 2012, **29**(8):1626-1637.

8. Bao F, Shultz SR, Hepburn JD, Omana V, Weaver LC, Cain DP, Brown A: **A CD11d monoclonal antibody treatment reduces tissue injury and improves neurological outcome after fluid percussion brain injury in rats.** J Neurotrauma 2012, **29**(14):2375-2392.

9. Bedi SS, Lago MT, Masha LI, Crook RJ, Grill RJ, Walters ET: **Spinal cord injury triggers an intrinsic growth-promoting state in nociceptors.** J Neurotrauma 2012, **29**(5):925-935.

10. Benedict AL, Mountney A, Hurtado A, Bryan KE, Schnaar RL, Dinkova-Kostova AT, Talalay P: **Neuroprotective effects of sulforaphane after contusive spinal cord injury.** J Neurotrauma 2012, **29**(16):2576-2586.

11. Blanie A, Vigue B, Benhamou D, Duranteau J, Geeraerts T: **The frontal lobe and thalamus have different sensitivities to hypoxia-hypotension after traumatic brain injury: a microdialysis study in rats.** J Neurotrauma 2012, **29**(18):2782-2790.

12. Bolkvadze T, Pitkanen A: **Development of post-traumatic epilepsy after controlled cortical impact and lateral fluid-percussion-induced brain injury in the mouse.** J Neurotrauma 2012, **29**(5):789-812.

13. Bonilla C, Zurita M, Otero L, Aguayo C, Rico MA, Rodriguez A, Vaquero J: **Failure of delayed intravenous administration of bone marrow stromal cells after traumatic brain injury.** J Neurotrauma 2012, **29**(2):394-400.

14. Cabaj AM, Slawinska U: **Riluzole treatment reduces motoneuron death induced by axotomy in newborn rats.** J Neurotrauma 2012, **29**(7):1506-1517.

15. Campbell JN, Low B, Kurz JE, Patel SS, Young MT, Churn SB: **Mechanisms of dendritic spine remodeling in a rat model of traumatic brain injury.** J Neurotrauma 2012, **29**(2):218-234.

16. Campbell JN, Register D, Churn SB: **Traumatic brain injury causes an FK506-sensitive loss and an overgrowth of dendritic spines in rat forebrain.** J Neurotrauma 2012, **29**(2):201-217.

17. Cengiz N, Ozturk G, Erdogan E, Him A, Oguz EK: **Consequences of neurite transection in vitro.** J Neurotrauma 2012, **29**(15):2465-2474.

18. Chen Z, Leung LY, Mountney A, Liao Z, Yang W, Lu XC, Dave J, Deng-Bryant Y, Wei G, Schmid K, Shear DA, Tortella FC: **A novel animal model of closed-head concussive-induced mild traumatic brain injury: development, implementation, and characterization.** J Neurotrauma 2012, **29**(2):268-280.

19. Cheng JP, Shaw KE, Monaco CM, Hoffman AN, Sozda CN, Olsen AS, Kline AE: **A relatively brief exposure to environmental enrichment after experimental traumatic brain injury confers long-term cognitive benefits.** J Neurotrauma 2012, **29**(17):2684-2688.

20. Coordes A, Groschel M, Ernst A, Basta D: **Apoptotic cascades in the central auditory pathway after noise exposure.** J Neurotrauma 2012, **29**(6):1249-1254.

21. Crawford F, Crynen G, Reed J, Mouzon B, Bishop A, Katz B, Ferguson S, Phillips J, Ganapathi V, Mathura V, Roses A, Mullan M: **Identification of plasma biomarkers of TBI outcome using proteomic approaches in an APOE mouse model.** J Neurotrauma 2012, **29**(2):246-260.

22. Eakin K, Miller JP: **Mild traumatic brain injury is associated with impaired hippocampal spatiotemporal representation in the absence of histological changes.** J Neurotrauma 2012, **29**(6):1180-1187.

23. Elder GA, Dorr NP, De Gasperi R, Gama Sosa MA, Shaughness MC, Maudlin-Jeronimo E, Hall AA, McCarron RM, Ahlers ST: **Blast exposure induces post-traumatic stress disorder-related traits in a rat model of mild traumatic brain injury.** J Neurotrauma 2012, **29**(16):2564-2575.

24. Elias PZ, Spector M: **Characterization of a bilateral penetrating brain injury in rats and evaluation of a collagen biomaterial for potential treatment.** J Neurotrauma 2012, **29**(11):2086-2102.

25. Feng JF, Zhao X, Gurkoff GG, Van KC, Shahlaie K, Lyeth BG: **Post-traumatic hypoxia exacerbates neuronal cell death in the hippocampus.** J Neurotrauma 2012, **29**(6):1167-1179.

26. Figueroa JD, Cordero K, Baldeosingh K, Torrado AI, Walker RL, Miranda JD, Leon MD: **Docosahexaenoic acid pretreatment confers protection and functional improvements after acute spinal cord injury in adult rats.** J Neurotrauma 2012, **29**(3):551-566.

27. Foerch C, You Z, Wang H, Lo EH, Whalen MJ: **Traumatic brain injury during warfarin anticoagulation: an experimental study in mice.** J Neurotrauma 2012, **29**(6):1150-1155.

28. Fujita M, Wei EP, Povlishock JT: **Effects of hypothermia on cerebral autoregulatory vascular responses in two rodent models of traumatic brain injury.** J Neurotrauma 2012, **29**(7):1491-1498.

29. Fujita M, Wei EP, Povlishock JT: **Intensity- and interval-specific repetitive traumatic brain injury can evoke both axonal and microvascular damage.** J Neurotrauma 2012, **29**(12):2172-2180.

30. Furutani R, Kibayashi K: **Morphological alteration and reduction of MAP2-immunoreactivity in pyramidal neurons of cerebral cortex in a rat model of focal cortical compression.** J Neurotrauma 2012, **29**(6):1266-1276.

31. Gao WS, Dong CJ, Li SQ, Kunwar KJ, Li B: **Re-innervation of the bladder through end-to-side neurorrhaphy of autonomic nerve and somatic nerve in rats.** J Neurotrauma 2012, **29**(8):1704-1713.

32. Gatson JW, Liu MM, Abdelfattah K, Wigginton JG, Smith S, Wolf S, Simpkins JW, Minei JP: **Estrone is neuroprotective in rats after traumatic brain injury.** J Neurotrauma 2012, **29**(12):2209-2219.

33. Geremia NM, Bao F, Rosenzweig TE, Hryciw T, Weaver L, Dekaban GA, Brown A: **CD11d Antibody Treatment Improves Recovery in Spinal Cord-Injured Mice.** J Neurotrauma 2012, **29**(3):539-550.

34. Gonzenbach RR, Zoerner B, Schnell L, Weinmann O, Mir AK, Schwab ME: **Delayed anti-nogo-a antibody application after spinal cord injury shows progressive loss of responsiveness.** J Neurotrauma 2012, **29**(3):567-578.

35. Griesbach GS, Tio DL, Vincelli J, McArthur DL, Taylor AN: **Differential effects of voluntary and forced exercise on stress responses after traumatic brain injury.** J Neurotrauma 2012, **29**(7):1426-1433.

36. Hama AT, Broadhead A, Lorrain DS, Sagen J: **The antinociceptive effect of the asthma drug ibudilast in rat models of peripheral and central neuropathic pain.** J Neurotrauma 2012, **29**(3):600-610.

37. Hanell A, Clausen F, Djupsjo A, Vallstedt A, Patra K, Israelsson C, Larhammar M, Bjork M, Paixao S, Kullander K, Marklund N: **Functional and histological outcome after focal traumatic brain injury is not improved in conditional EphA4 knockout mice.** J Neurotrauma 2012, **29**(17):2660-2671.

38. Harris NG, Mironova YA, Chen SF, Richards HK, Pickard JD: **Preventing flow-metabolism uncoupling acutely reduces axonal injury after traumatic brain injury.** J Neurotrauma 2012, **29**(7):1469-1482.

39. Hemerka JN, Wu X, Dixon CE, Garman RH, Exo JL, Shellington DK, Blasiole B, Vagni VA, Janesko-Feldman K, Xu M, Wisniewski SR, Bayir H, Jenkins LW, Clark RS, Tisherman SA, Kochanek PM: **Severe brief pressure-controlled hemorrhagic shock after traumatic brain injury exacerbates functional deficits and long-term neuropathological damage in mice.** J Neurotrauma 2012, **29**(12):2192-2208.

40. Hill CE, Brodak DM, Bartlett Bunge M: **Dissociated predegenerated peripheral nerve transplants for spinal cord injury repair: a comprehensive assessment of their effects on regeneration and functional recovery compared to Schwann cell transplants.** J Neurotrauma 2012, **29**(12):2226-2243.

41. Hinzman JM, Thomas TC, Quintero JE, Gerhardt GA, Lifshitz J: **Disruptions in the regulation of extracellular glutamate by neurons and glia in the rat striatum two days after diffuse brain injury.** J Neurotrauma 2012, **29**(6):1197-1208.

42. Hussain ZM, Fitting S, Watanabe H, Usynin I, Yakovleva T, Knapp PE, Scheff SW, Hauser KF, Bakalkin G: **Lateralized response of dynorphin a peptide levels after traumatic brain injury.** J Neurotrauma 2012, **29**(9):1785-1793.

43. Indraswari F, Wang H, Lei B, James ML, Kernagis D, Warner DS, Dawson HN, Laskowitz DT: **Statins improve outcome in murine models of intracranial hemorrhage and traumatic brain injury: a translational approach.** J Neurotrauma 2012, **29**(7):1388-1400.

44. Inskip JA, Ramer LM, Ramer MS, Krassioukov AV, Claydon VE: **Spectral analyses of cardiovascular control in rodents with spinal cord injury.** J Neurotrauma 2012, **29**(8):1638-1649.

45. Ji J, Tyurina YY, Tang M, Feng W, Stolz DB, Clark RS, Meaney DF, Kochanek PM, Kagan VE, Bayir H: **Mitochondrial injury after mechanical stretch of cortical neurons in vitro: biomarkers of apoptosis and selective peroxidation of anionic phospholipids.** J Neurotrauma 2012, **29**(5):776-788.

46. Jiang Y, Brody DL: **Administration of COG1410 reduces axonal amyloid precursor protein immunoreactivity and microglial activation after controlled cortical impact in mice.** J Neurotrauma 2012, **29**(13):2332-2341.

47. Jones TA, Liput DJ, Maresh EL, Donlan N, Parikh TJ, Marlowe D, Kozlowski DA: **Use-dependent dendritic regrowth is limited after unilateral controlled cortical impact to the forelimb sensorimotor cortex.** J Neurotrauma 2012, **29**(7):1455-1468.

48. Kabadi SV, Stoica BA, Loane DJ, Byrnes KR, Hanscom M, Cabatbat RM, Tan MT, Faden AI: **Cyclin D1 gene ablation confers neuroprotection in traumatic brain injury.** J Neurotrauma 2012, **29**(5):813-827.

49. Kamei N, Kwon SM, Ishikawa M, Ii M, Nakanishi K, Yamada K, Hozumi K, Kawamoto A, Ochi M, Asahara T: **Endothelial progenitor cells promote astrogliosis following spinal cord injury through Jagged1-dependent Notch signaling.** J Neurotrauma 2012, **29**(9):1758-1769.

50. Kesherwani V, Agrawal SK: **Upregulation of RyR2 in hypoxic/reperfusion injury.** J Neurotrauma 2012, **29**(6):1255-1265.

51. Khaing ZZ, Geissler SA, Jiang S, Milman BD, Aguilar SV, Schmidt CE, Schallert T: **Assessing forelimb function after unilateral cervical spinal cord injury: novel forelimb tasks predict lesion severity and recovery.** J Neurotrauma 2012, **29**(3):488-498.

52. Khuman J, Zhang J, Park J, Carroll JD, Donahue C, Whalen MJ: **Low-level laser light therapy improves cognitive deficits and inhibits microglial activation after controlled cortical impact in mice.** J Neurotrauma 2012, **29**(2):408-417.

53. Kim MS, Seo DH, Lim MH, Kim TU, Lee SJ, Hyun JK: **Skin temperature changes following sciatic nerve injury in rats.** J Neurotrauma 2012, **29**(18):2738-2747.

54. Kline AE, Olsen AS, Sozda CN, Hoffman AN, Cheng JP: **Evaluation of a combined treatment paradigm consisting of environmental enrichment and the 5-HT1A receptor agonist buspirone after experimental traumatic brain injury.** J Neurotrauma 2012, **29**(10):1960-1969.

55. Kobayashi S, Kato K, Rodriguez Guerrero A, Baba H, Yoshizawa H: **Experimental syringohydromyelia induced by adhesive arachnoiditis in the rabbit: changes in the blood-spinal cord barrier, neuroinflammatory foci, and syrinx formation.** J Neurotrauma 2012, **29**(9):1803-1816.

56. Koopmans GC, Deumens R, Honig WM, Hamers FP, Mey J, van Kleef M, Joosten EA: **Functional recovery, serotonergic sprouting, and endogenous progenitor fates in response to delayed environmental enrichment after spinal cord injury.** J Neurotrauma 2012, **29**(3):514-527.

57. Kovacic U, Zele T, Tomsic M, Sketelj J, Bajrovic FF: **Influence of breaching the connective sheaths of the donor nerve on its myelinated sensory axons and on their sprouting into the end-to-side coapted nerve in the rat.** J Neurotrauma 2012, **29**(18):2805-2815.

58. Krisa L, Frederick KL, Canver JC, Stackhouse SK, Shumsky JS, Murray M: **Amphetamine-enhanced motor training after cervical contusion injury.** J Neurotrauma 2012, **29**(5):971-989.

59. Lee HJ, Bian S, Jakovcevski I, Wu B, Irintchev A, Schachner M: **Delayed applications of L1 and chondroitinase ABC promote recovery after spinal cord injury.** J Neurotrauma 2012, **29**(10):1850-1863.

60. Lee J, Satkunendrarajah K, Fehlings MG: **Development and characterization of a novel rat model of cervical spondylotic myelopathy: the impact of chronic cord compression on clinical, neuroanatomical, and neurophysiological outcomes.** J Neurotrauma 2012, **29**(5):1012-1027.

61. Lei Z, Deng P, Li J, Xu ZC: **Alterations of A-type potassium channels in hippocampal neurons after traumatic brain injury.** J Neurotrauma 2012, **29**(2):235-245.

62. Li J, Gu L, Feng DF, Ding F, Zhu G, Rong J: **Exploring temporospatial changes in glucose metabolic disorder, learning, and memory dysfunction in a rat model of diffuse axonal injury.** J Neurotrauma 2012, **29**(17):2635-2646.

63. Li Z, Wang B, Kan Z, Zhang B, Yang Z, Chen J, Wang D, Wei H, Zhang JN, Jiang R: **Progesterone increases circulating endothelial progenitor cells and induces neural regeneration after traumatic brain injury in aged rats.** J Neurotrauma 2012, **29**(2):343-353.

64. Lin CY, Lee YS, Lin VW, Silver J: **Fibronectin inhibits chronic pain development after spinal cord injury.** J Neurotrauma 2012, **29**(3):589-599.

65. Lopez NE, Krzyzaniak MJ, Blow C, Putnam J, Ortiz-Pomales Y, Hageny AM, Eliceiri B, Coimbra R, Bansal V: **Ghrelin prevents disruption of the blood-brain barrier after traumatic brain injury.** J Neurotrauma 2012, **29**(2):385-393.

66. Lutton C, Young YW, Williams R, Meedeniya AC, Mackay-Sim A, Goss B: **Combined VEGF and PDGF treatment reduces secondary degeneration after spinal cord injury.** J Neurotrauma 2012, **29**(5):957-970.

67. Ma M, Shofer FS, Neumar RW: **Calpastatin overexpression protects axonal transport in an in vivo model of traumatic axonal injury.** J Neurotrauma 2012, **29**(16):2555-2563.

68. Mackenzie SJ, Smirnov I, Calancie B: **Cauda equina repair in the rat: part 2. Time course of ventral root conduction failure.** J Neurotrauma 2012, **29**(8):1683-1690.

69. Mao JC, Pace E, Pierozynski P, Kou Z, Shen Y, VandeVord P, Haacke EM, Zhang X, Zhang J: **Blast-induced tinnitus and hearing loss in rats: behavioral and imaging assays.** J Neurotrauma 2012, **29**(2):430-444.

70. Mao SS, Hua R, Zhao XP, Qin X, Sun ZQ, Zhang Y, Wu YQ, Jia MX, Cao JL, Zhang YM: **Exogenous administration of PACAP alleviates traumatic brain injury in rats through a mechanism involving the TLR4/MyD88/NF-kappaB pathway.** J Neurotrauma 2012, **29**(10):1941-1959.

71. Martens KM, Vonder Haar C, Hutsell BA, Hoane MR: **A discrimination task used as a novel method of testing decision-making behavior following traumatic brain injury.** J Neurotrauma 2012, **29**(15):2505-2512.

72. Mesfin MN, von Reyn CR, Mott RE, Putt ME, Meaney DF: **In vitro stretch injury induces time- and severity-dependent alterations of STEP phosphorylation and proteolysis in neurons.** J Neurotrauma 2012, **29**(10):1982-1998.

73. Moinard C, Butel MJ, Bureau MF, Choisy C, Waligora-Dupriet AJ, Moulis J, Marc J, Cynober L, Charrueau C: **In vivo bioluminescent imaging of a new model of infectious complications in head-injury rats.** J Neurotrauma 2012, **29**(2):335-342.

74. Mouzon B, Chaytow H, Crynen G, Bachmeier C, Stewart J, Mullan M, Stewart W, Crawford F: **Repetitive mild traumatic brain injury in a mouse model produces learning and memory deficits accompanied by histological changes.** J Neurotrauma 2012, **29**(18):2761-2773.

75. Myers SA, DeVries WH, Gruenthal MJ, Andres KR, Hagg T, Whittemore SR: **Sildenafil improves epicenter vascular perfusion but not hindlimb functional recovery after contusive spinal cord injury in mice.** J Neurotrauma 2012, **29**(3):528-538.

76. Nakajima H, Uchida K, Guerrero AR, Watanabe S, Sugita D, Takeura N, Yoshida A, Long G, Wright KT, Johnson WE, Baba H: **Transplantation of mesenchymal stem cells promotes an alternative pathway of macrophage activation and functional recovery after spinal cord injury.** J Neurotrauma 2012, **29**(8):1614-1625.

77. Navarro JC, Pillai S, Cherian L, Garcia R, Grill RJ, Robertson CS: **Histopathological and behavioral effects of immediate and delayed hemorrhagic shock after mild traumatic brain injury in rats.** J Neurotrauma 2012, **29**(2):322-334.

78. Ng SY, Semple BD, Morganti-Kossmann MC, Bye N: **Attenuation of microglial activation with minocycline is not associated with changes in neurogenesis after focal traumatic brain injury in adult mice.** J Neurotrauma 2012, **29**(7):1410-1425.

79. Nicaise C, Putatunda R, Hala TJ, Regan KA, Frank DM, Brion JP, Leroy K, Pochet R, Wright MC, Lepore AC: **Degeneration of phrenic motor neurons induces long-term diaphragm deficits following mid-cervical spinal contusion in mice.** J Neurotrauma 2012, **29**(18):2748-2760.

80. Nito C, Kamada H, Endo H, Narasimhan P, Lee YS, Chan PH: **Involvement of mitogen-activated protein kinase pathways in expression of the water channel protein aquaporin-4 after ischemia in rat cortical astrocytes.** J Neurotrauma 2012, **29**(14):2404-2412.

81. Nout YS, Beattie MS, Bresnahan JC: **Severity of locomotor and cardiovascular derangements after experimental high-thoracic spinal cord injury is anesthesia dependent in rats.** J Neurotrauma 2012, **29**(5):990-999.

82. Ohayon S, Boyko M, Saad A, Douvdevani A, Gruenbaum BF, Melamed I, Shapira Y, Teichberg VI, Zlotnik A: **Cell-free DNA as a marker for prediction of brain damage in traumatic brain injury in rats.** J Neurotrauma 2012, **29**(2):261-267.

83. Ohri SS, Maddie MA, Zhang Y, Shields CB, Hetman M, Whittemore SR: **Deletion of the pro-apoptotic endoplasmic reticulum stress response effector CHOP does not result in improved locomotor function after severe contusive spinal cord injury.** J Neurotrauma 2012, **29**(3):579-588.

84. Olsen AS, Sozda CN, Cheng JP, Hoffman AN, Kline AE: **Traumatic brain injury-induced cognitive and histological deficits are attenuated by delayed and chronic treatment with the 5-HT1A-receptor agonist buspirone.** J Neurotrauma 2012, **29**(10):1898-1907.

85. Oron A, Oron U, Streeter J, De Taboada L, Alexandrovich A, Trembovler V, Shohami E: **Near infrared transcranial laser therapy applied at various modes to mice following traumatic brain injury significantly reduces long-term neurological deficits.** J Neurotrauma 2012, **29**(2):401-407.

86. Peterson TC, Anderson GD, Kantor ED, Hoane MR: **A comparison of the effects of nicotinamide and progesterone on functional recovery of cognitive behavior following cortical contusion injury in the rat.** J Neurotrauma 2012, **29**(18):2823-2830.

87. Pham V, Albiston AL, Downes CE, Wong CH, Diwakarla S, Ng L, Lee S, Crack PJ, Chai SY: **Insulin-regulated aminopeptidase deficiency provides protection against ischemic stroke in mice.** J Neurotrauma 2012, **29**(6):1243-1248.

88. Plantman S, Ng KC, Lu J, Davidsson J, Risling M: **Characterization of a novel rat model of penetrating traumatic brain injury.** J Neurotrauma 2012, **29**(6):1219-1232.

89. Raible DJ, Frey LC, Cruz Del Angel Y, Russek SJ, Brooks-Kayal AR: **GABA(A) receptor regulation after experimental traumatic brain injury.** J Neurotrauma 2012, **29**(16):2548-2554.

90. Robertson CS, Cherian L, Shah M, Garcia R, Navarro JC, Grill RJ, Hand CC, Tian TS, Hannay HJ: **Neuroprotection with an erythropoietin mimetic peptide (pHBSP) in a model of mild traumatic brain injury complicated by hemorrhagic shock.** J Neurotrauma 2012, **29**(6):1156-1166.

91. Robins-Steele S, Nguyen DH, Fehlings MG: **The delayed post-injury administration of soluble fas receptor attenuates post-traumatic neural degeneration and enhances functional recovery after traumatic cervical spinal cord injury.** J Neurotrauma 2012, **29**(8):1586-1599.

92. Rodgers KM, Bercum FM, McCallum DL, Rudy JW, Frey LC, Johnson KW, Watkins LR, Barth DS: **Acute neuroimmune modulation attenuates the development of anxiety-like freezing behavior in an animal model of traumatic brain injury.** J Neurotrauma 2012, **29**(10):1886-1897.

93. Sakurai A, Atkins CM, Alonso OF, Bramlett HM, Dietrich WD: **Mild hyperthermia worsens the neuropathological damage associated with mild traumatic brain injury in rats.** J Neurotrauma 2012, **29**(2):313-321.

94. Santos-Nogueira E, Redondo Castro E, Mancuso R, Navarro X: **Randall-Selitto test: a new approach for the detection of neuropathic pain after spinal cord injury.** J Neurotrauma 2012, **29**(5):898-904.

95. Sarntinoranont M, Lee SJ, Hong Y, King MA, Subhash G, Kwon J, Moore DF: **High-strain-rate brain injury model using submerged acute rat brain tissue slices.** J Neurotrauma 2012, **29**(2):418-429.

96. Saxena T, Gilbert J, Stelzner D, Hasenwinkel J: **Mechanical characterization of the injured spinal cord after lateral spinal hemisection injury in the rat.** J Neurotrauma 2012, **29**(9):1747-1757.

97. Schober ME, Ke X, Xing B, Block BP, Requena DF, McKnight R, Lane RH: **Traumatic brain injury increased IGF-1B mRNA and altered IGF-1 exon 5 and promoter region epigenetic characteristics in the rat pup hippocampus.** J Neurotrauma 2012, **29**(11):2075-2085.

98. Scremin OU, Norman KM, Roch M, Holschneider DP, Scremin AM: **Acetylcholinesterase inhibition interacts with training to reverse spatial learning deficits after cortical impact injury.** J Neurotrauma 2012, **29**(15):2457-2464.

99. Sekiguchi A, Kanno H, Ozawa H, Yamaya S, Itoi E: **Rapamycin promotes autophagy and reduces neural tissue damage and locomotor impairment after spinal cord injury in mice.** J Neurotrauma 2012, **29**(5):946-956.

100. Semple BD, Canchola SA, Noble-Haeusslein LJ: **Deficits in social behavior emerge during development after pediatric traumatic brain injury in mice.** J Neurotrauma 2012, **29**(17):2672-2683.

101. Shin SS, Bray ER, Dixon CE: **Effects of nicotine administration on striatal dopamine signaling after traumatic brain injury in rats.** J Neurotrauma 2012, **29**(5):843-850.

102. Shin YJ, Kim HL, Park JM, Cho JM, Kim CY, Choi KJ, Kweon HS, Cha JH, Lee MY: **Overlapping distribution of osteopontin and calcium in the ischemic core of rat brain after transient focal ischemia.** J Neurotrauma 2012, **29**(7):1530-1538.

103. Shultz SR, Bao F, Omana V, Chiu C, Brown A, Cain DP: **Repeated mild lateral fluid percussion brain injury in the rat causes cumulative long-term behavioral impairments, neuroinflammation, and cortical loss in an animal model of repeated concussion.** J Neurotrauma 2012, **29**(2):281-294.

104. Siddiq I, Park E, Liu E, Spratt SK, Surosky R, Lee G, Ando D, Giedlin M, Hare GM, Fehlings MG, Baker AJ: **Treatment of traumatic brain injury using zinc-finger protein gene therapy targeting VEGF-A.** J Neurotrauma 2012, **29**(17):2647-2659.

105. Siopi E, Calabria S, Plotkine M, Marchand-Leroux C, Jafarian-Tehrani M: **Minocycline restores olfactory bulb volume and olfactory behavior after traumatic brain injury in mice.** J Neurotrauma 2012, **29**(2):354-361.

106. Sukumari-Ramesh S, Alleyne CH,Jr, Dhandapani KM: **Astrocyte-specific expression of survivin after intracerebral hemorrhage in mice: a possible role in reactive gliosis?** J Neurotrauma 2012, **29**(18):2798-2804.

107. Sun W, Fu Y, Shi Y, Cheng JX, Cao P, Shi R: **Paranodal myelin damage after acute stretch in Guinea pig spinal cord.** J Neurotrauma 2012, **29**(3):611-619.

108. Sundaramurthy A, Alai A, Ganpule S, Holmberg A, Plougonven E, Chandra N: **Blast-induced biomechanical loading of the rat: an experimental and anatomically accurate computational blast injury model.** J Neurotrauma 2012, **29**(13):2352-2364.

109. Thau-Zuchman O, Shohami E, Alexandrovich AG, Trembovler V, Leker RR: **The anti-inflammatory drug carprofen improves long-term outcome and induces gliogenesis after traumatic brain injury.** J Neurotrauma 2012, **29**(2):375-384.

110. Thomas TC, Hinzman JM, Gerhardt GA, Lifshitz J: **Hypersensitive glutamate signaling correlates with the development of late-onset behavioral morbidity in diffuse brain-injured circuitry.** J Neurotrauma 2012, **29**(2):187-200.

111. Tu Y, Chen C, Sun HT, Cheng SX, Liu XZ, Qu Y, Li XH, Zhang S: **Combination of temperature-sensitive stem cells and mild hypothermia: a new potential therapy for severe traumatic brain injury.** J Neurotrauma 2012, **29**(14):2393-2403.

112. Voor MJ, Brown EH, Xu Q, Waddell SW, Burden RL,Jr, Burke DA, Magnuson DS: **Bone loss following spinal cord injury in a rat model.** J Neurotrauma 2012, **29**(8):1676-1682.

113. Wang E, Gao J, Yang Q, Parsley MO, Dunn TJ, Zhang L, DeWitt DS, Denner L, Prough DS, Wu P: **Molecular mechanisms underlying effects of neural stem cells against traumatic axonal injury.** J Neurotrauma 2012, **29**(2):295-312.

114. Wang LC, Huang CY, Wang HK, Wu MH, Tsai KJ: **Magnesium sulfate and nimesulide have synergistic effects on rescuing brain damage after transient focal ischemia.** J Neurotrauma 2012, **29**(7):1518-1529.

115. Wang X, de Rivero Vaccari JP, Wang H, Diaz P, German R, Marcillo AE, Keane RW: **Activation of the nuclear factor E2-related factor 2/antioxidant response element pathway is neuroprotective after spinal cord injury.** J Neurotrauma 2012, **29**(5):936-945.

116. Ward PJ, Hubscher CH: **Persistent polyuria in a rat spinal contusion model.** J Neurotrauma 2012, **29**(15):2490-2498.

117. Warren KM, Reeves TM, Phillips LL: **MT5-MMP, ADAM-10, and N-cadherin act in concert to facilitate synapse reorganization after traumatic brain injury.** J Neurotrauma 2012, **29**(10):1922-1940.

118. Washington PM, Forcelli PA, Wilkins T, Zapple DN, Parsadanian M, Burns MP: **The effect of injury severity on behavior: a phenotypic study of cognitive and emotional deficits after mild, moderate, and severe controlled cortical impact injury in mice.** J Neurotrauma 2012, **29**(13):2283-2296.

119. Weckbach S, Neher M, Losacco JT, Bolden AL, Kulik L, Flierl MA, Bell SE, Holers VM, Stahel PF: **Challenging the role of adaptive immunity in neurotrauma: Rag1(-/-) mice lacking mature B and T cells do not show neuroprotection after closed head injury.** J Neurotrauma 2012, **29**(6):1233-1242.

120. Wei XE, Zhang YZ, Li YH, Li MH, Li WB: **Dynamics of rabbit brain edema in focal lesion and perilesion area after traumatic brain injury: a MRI study.** J Neurotrauma 2012, **29**(14):2413-2420.

121. Willie JT, Lim MM, Bennett RE, Azarion AA, Schwetye KE, Brody DL: **Controlled cortical impact traumatic brain injury acutely disrupts wakefulness and extracellular orexin dynamics as determined by intracerebral microdialysis in mice.** J Neurotrauma 2012, **29**(10):1908-1921.

122. Woller SA, Moreno GL, Hart N, Wellman PJ, Grau JW, Hook MA: **Analgesia or addiction?: implications for morphine use after spinal cord injury.** J Neurotrauma 2012, **29**(8):1650-1662.

123. Won SJ, Choi BY, Yoo BH, Sohn M, Ying W, Swanson RA, Suh SW: **Prevention of traumatic brain injury-induced neuron death by intranasal delivery of nicotinamide adenine dinucleotide.** J Neurotrauma 2012, **29**(7):1401-1409.

124. Wu Y, Zhao J, Zhao W, Pan J, Bauman WA, Cardozo CP: **Nandrolone normalizes determinants of muscle mass and fiber type after spinal cord injury.** J Neurotrauma 2012, **29**(8):1663-1675.

125. Yang S, Ma Y, Liu Y, Que H, Zhu C, Liu S: **Arachidonic acid: a bridge between traumatic brain injury and fracture healing.** J Neurotrauma 2012, **29**(17):2696-2705.

126. Yu F, Wang Z, Tchantchou F, Chiu CT, Zhang Y, Chuang DM: **Lithium ameliorates neurodegeneration, suppresses neuroinflammation, and improves behavioral performance in a mouse model of traumatic brain injury.** J Neurotrauma 2012, **29**(2):362-374.

127. Yu F, Zhang Y, Chuang DM: **Lithium reduces BACE1 overexpression, beta amyloid accumulation, and spatial learning deficits in mice with traumatic brain injury.** J Neurotrauma 2012, **29**(13):2342-2351.

128. Yuan F, Tang Y, Lin X, Xi Y, Guan Y, Xiao T, Chen J, Zhang Z, Yang GY, Wang Y: **Optimizing suture middle cerebral artery occlusion model in C57BL/6 mice circumvents posterior communicating artery dysplasia.** J Neurotrauma 2012, **29**(7):1499-1505.

129. Zhang L, Liu J, Cheng C, Yuan Y, Yu B, Shen A, Yan M: **The neuroprotective effect of pyrroloquinoline quinone on traumatic brain injury.** J Neurotrauma 2012, **29**(5):851-864.

130. Zhao J, Pati S, Redell JB, Zhang M, Moore AN, Dash PK: **Caffeic Acid phenethyl ester protects blood-brain barrier integrity and reduces contusion volume in rodent models of traumatic brain injury.** J Neurotrauma 2012, **29**(6):1209-1218.

131. Zhao Z, Loane DJ, Murray MG,2nd, Stoica BA, Faden AI: **Comparing the predictive value of multiple cognitive, affective, and motor tasks after rodent traumatic brain injury.** J Neurotrauma 2012, **29**(15):2475-2489.

**17. Resuscitation**

1. Aksu U, Bezemer R, Yavuz B, Kandil A, Demirci C, Ince C: **Balanced vs unbalanced crystalloid resuscitation in a near-fatal model of hemorrhagic shock and the effects on renal oxygenation, oxidative stress, and inflammation.** Resuscitation 2012, **83**(6):767-773.

2. Almac E, Aksu U, Bezemer R, Jong W, Kandil A, Yuruk K, Demirci-Tansel C, Ince C: **The acute effects of acetate-balanced colloid and crystalloid resuscitation on renal oxygenation in a rat model of hemorrhagic shock.** Resuscitation 2012, **83**(9):1166-1172.

3. Drabek T, Janata A, Jackson EK, End B, Stezoski J, Vagni VA, Janesko-Feldman K, Wilson CD, van Rooijen N, Tisherman SA, Kochanek PM: **Microglial depletion using intrahippocampal injection of liposome-encapsulated clodronate in prolonged hypothermic cardiac arrest in rats.** Resuscitation 2012, **83**(4):517-526.

4. Fang X, Huang Z, Zhu J, Jiang L, Li H, Fu Y, Sun S, Tang W: **Ultrastructural evidence of mitochondrial abnormalities in postresuscitation myocardial dysfunction.** Resuscitation 2012, **83**(3):386-394.

5. Gong J, Guo S, Li HB, Yuan SY, Shang Y, Yao SL: **BML-111, a lipoxin receptor agonist, protects haemorrhagic shock-induced acute lung injury in rats.** Resuscitation 2012, **83**(7):907-912.

6. Kawano Y, Kawaguchi M, Hirota K, Kai S, Konishi N, Furuya H: **Effects of n-propyl gallate on neuronal survival after forebrain ischemia in rats.** Resuscitation 2012, **83**(2):249-252.

7. Kida K, Minamishima S, Wang H, Ren J, Yigitkanli K, Nozari A, Mandeville JB, Liu PK, Liu CH, Ichinose F: **Sodium sulfide prevents water diffusion abnormality in the brain and improves long term outcome after cardiac arrest in mice.** Resuscitation 2012, **83**(10):1292-1297.

8. Kim K, Li Y, Jin G, Chong W, Liu B, Lu J, Lee K, Demoya M, Velmahos GC, Alam HB: **Effect of valproic acid on acute lung injury in a rodent model of intestinal ischemia reperfusion.** Resuscitation 2012, **83**(2):243-248.

9. Liu H, Sarnaik SM, Manole MD, Chen Y, Shinde SN, Li W, Rose M, Alexander H, Chen J, Clark RS, Graham SH, Hickey RW: **Increased cytochrome c in rat cerebrospinal fluid after cardiac arrest and its effects on hypoxic neuronal survival.** Resuscitation 2012, **83**(12):1491-1496.

10. Mersmann J, Tran N, Latsch K, Habeck K, Iskandar F, Zimmermann R, Zacharowski K: **Akt or phosphoinositide-3-kinase inhibition reverses cardio-protection in Toll-like receptor 2 deficient mice.** Resuscitation 2012, **83**(11):1404-1410.

11. Mochizuki T, Yu S, Katoh T, Aoki K, Sato S: **Cardioprotective effect of therapeutic hypothermia at 34 degrees C against ischaemia/reperfusion injury mediated by PI3K and nitric oxide in a rat isolated heart model.** Resuscitation 2012, **83**(2):238-242.

12. Paine MG, Che D, Li L, Neumar RW: **Cerebellar Purkinje cell neurodegeneration after cardiac arrest: effect of therapeutic hypothermia.** Resuscitation 2012, **83**(12):1511-1516.

13. Schneider A, Teschendorf P, Vogel P, Russ N, Knapp J, Bottiger BW, Popp E: **Facilitation of hypothermia by quinpirole and 8-OH-DPAT in a rat model of cardiac arrest.** Resuscitation 2012, **83**(2):232-237.

14. Taguchi N, Nakayama S, Tanaka M: **Fluoxetine has neuroprotective effects after cardiac arrest and cardiopulmonary resuscitation in mouse.** Resuscitation 2012, **83**(5):652-656.

15. Wang T, Sun S, Wan Z, Weil MH, Tang W: **Effects of bone marrow mesenchymal stem cells in a rat model of myocardial infarction.** Resuscitation 2012, **83**(11):1391-1396.

16. Weng Y, Sun S, Park J, Ye S, Weil MH, Tang W: **Cannabinoid 1 (CB1) receptor mediates WIN55, 212-2 induced hypothermia and improved survival in a rat post-cardiac arrest model.** Resuscitation 2012, **83**(9):1145-1151.

17. Wu CL, Su SB, Chen JL, Chang CP, Guo HR: **Tetramethylammonium ion causes respiratory failure related mortality in a rat model.** Resuscitation 2012, **83**(1):119-124.

18. Yin XL, Zhang W, Yang Y, Shen H: **Increasing expression of (CCAAT enhancer binding protein) homologous protein induced by endoplasmic reticulum stress in myocardium after cardiac arrest and resuscitation in rat.** Resuscitation 2012, **83**(3):378-385.

**18. Neurocritical Care**

No eligible articles were identified.

**19. Current Opinion in Critical Care**

No eligible articles were identified.

**20. Seminars in Respiratory and Critical Care Medicine**

No eligible articles were identified.

**III. Category: Respiratory System**

**21. Thorax**

1. Bastarache JA, Sebag SC, Clune JK, Grove BS, Lawson WE, Janz DR, Roberts LJ,2nd, Dworski R, Mackman N, Ware LB: **Low levels of tissue factor lead to alveolar haemorrhage, potentiating murine acute lung injury and oxidative stress.** Thorax 2012, **67**(12):1032-1039.

2. Bertok S, Wilson MR, Morley PJ, de Wildt R, Bayliffe A, Takata M: **Selective inhibition of intra-alveolar p55 TNF receptor attenuates ventilator-induced lung injury.** Thorax 2012, **67**(3):244-251.

3. Curley GF, Hayes M, Ansari B, Shaw G, Ryan A, Barry F, O'Brien T, O'Toole D, Laffey JG: **Mesenchymal stem cells enhance recovery and repair following ventilator-induced lung injury in the rat.** Thorax 2012, **67**(6):496-501.

4. Essilfie AT, Simpson JL, Dunkley ML, Morgan LC, Oliver BG, Gibson PG, Foster PS, Hansbro PM: **Combined Haemophilus influenzae respiratory infection and allergic airways disease drives chronic infection and features of neutrophilic asthma.** Thorax 2012, **67**(7):588-599.

5. Ghobadi G, Bartelds B, van der Veen SJ, Dickinson MG, Brandenburg S, Berger RM, Langendijk JA, Coppes RP, van Luijk P: **Lung irradiation induces pulmonary vascular remodelling resembling pulmonary arterial hypertension.** Thorax 2012, **67**(4):334-341.

6. Grace M, Birrell MA, Dubuis E, Maher SA, Belvisi MG: **Transient receptor potential channels mediate the tussive response to prostaglandin E2 and bradykinin.** Thorax 2012, **67**(10):891-900.

7. Gupta N, Krasnodembskaya A, Kapetanaki M, Mouded M, Tan X, Serikov V, Matthay MA: **Mesenchymal stem cells enhance survival and bacterial clearance in murine Escherichia coli pneumonia.** Thorax 2012, **67**(6):533-539.

8. Marriott HM, Daigneault M, Thompson AA, Walmsley SR, Gill SK, Witcher DR, Wroblewski VJ, Hellewell PG, Whyte MK, Dockrell DH: **A decoy receptor 3 analogue reduces localised defects in phagocyte function in pneumococcal pneumonia.** Thorax 2012, **67**(11):985-992.

9. McGrath EE, Lawrie A, Marriott HM, Mercer P, Cross SS, Arnold N, Singleton V, Thompson AA, Walmsley SR, Renshaw SA, Sabroe I, Chambers RC, Dockrell DH, Whyte MK: **Deficiency of tumour necrosis factor-related apoptosis-inducing ligand exacerbates lung injury and fibrosis.** Thorax 2012, **67**(9):796-803.

10. Mutlu GM, Budinger GR, Wu M, Lam AP, Zirk A, Rivera S, Urich D, Chiarella SE, Go LH, Ghosh AK, Selman M, Pardo A, Varga J, Kamp DW, Chandel NS, Sznajder JI, Jain M: **Proteasomal inhibition after injury prevents fibrosis by modulating TGF-beta(1) signalling.** Thorax 2012, **67**(2):139-146.

11. Post S, Nawijn MC, Hackett TL, Baranowska M, Gras R, van Oosterhout AJ, Heijink IH: **The composition of house dust mite is critical for mucosal barrier dysfunction and allergic sensitisation.** Thorax 2012, **67**(6):488-495.

**22. European Respiratory Journal**

1. Chevalier E, Lagente V, Dupont M, Fargeau H, Palazzi X, Richard V, Dassaud M, Fric M, Coupe M, Carre C, Leduc S, Bernardelli P, Vergne F, Berna P, Bertrand CP: **Lack of involvement of type 7 phosphodiesterase in an experimental model of asthma.** Eur Respir J 2012, **39**(3):582-588.

2. Cho WS, Duffin R, Bradley M, Megson IL, Macnee W, Howie SE, Donaldson K: **NiO and Co3O4 nanoparticles induce lung DTH-like responses and alveolar lipoproteinosis.** Eur Respir J 2012, **39**(3):546-557.

3. Courboulin A, Barrier M, Perreault T, Bonnet P, Tremblay VL, Paulin R, Tremblay E, Lambert C, Jacob MH, Bonnet SN, Provencher S, Bonnet S: **Plumbagin reverses proliferation and resistance to apoptosis in experimental PAH.** Eur Respir J 2012, **40**(3):618-629.

4. Del Rio R, Moya EA, Parga MJ, Madrid C, Iturriaga R: **Carotid body inflammation and cardiorespiratory alterations in intermittent hypoxia.** Eur Respir J 2012, **39**(6):1492-1500.

5. Dumas de La Roque E, Bellance N, Rossignol R, Begueret H, Billaud M, dos Santos P, Ducret T, Marthan R, Dahan D, Ramos-Barbon D, Amor-Carro O, Savineau JP, Fayon M: **Dehydroepiandrosterone reverses chronic hypoxia/reoxygenation-induced right ventricular dysfunction in rats.** Eur Respir J 2012, **40**(6):1420-1429.

6. Grommes J, Morgelin M, Soehnlein O: **Pioglitazone attenuates endotoxin-induced acute lung injury by reducing neutrophil recruitment.** Eur Respir J 2012, **40**(2):416-423.

7. Hennus MP, Janssen R, Pennings JL, Hodemaekers HM, Kruijsen D, Jansen NJ, Meyaard L, van Vught AJ, Bont LJ: **Host response to mechanical ventilation for viral respiratory tract infection.** Eur Respir J 2012, **40**(6):1508-1515.

8. Lee YJ, Moon C, Lee SH, Park HJ, Seoh JY, Cho MS, Kang JL: **Apoptotic cell instillation after bleomycin attenuates lung injury through hepatocyte growth factor induction.** Eur Respir J 2012, **40**(2):424-435.

9. Mubarak KK, Montes-Worboys A, Regev D, Nasreen N, Mohammed KA, Faruqi I, Hensel E, Baz MA, Akindipe OA, Fernandez-Bussy S, Nathan SD, Antony VB: **Parenchymal trafficking of pleural mesothelial cells in idiopathic pulmonary fibrosis.** Eur Respir J 2012, **39**(1):133-140.

10. Patel BV, Wilson MR, Takata M: **Resolution of acute lung injury and inflammation: a translational mouse model.** Eur Respir J 2012, **39**(5):1162-1170.

11. Pichon A, Connes P, Quidu P, Marchant D, Brunet J, Levy BI, Vilar J, Safeukui I, Cymbalista F, Maignan M, Richalet JP, Favret F: **Acetazolamide and chronic hypoxia: effects on haemorheology and pulmonary haemodynamics.** Eur Respir J 2012, **40**(6):1401-1409.

12. Reynolds AM, Holmes MD, Danilov SM, Reynolds PN: **Targeted gene delivery of BMPR2 attenuates pulmonary hypertension.** Eur Respir J 2012, **39**(2):329-343.

13. Sonar SS, Ehmke M, Marsh LM, Dietze J, Dudda JC, Conrad ML, Renz H, Nockher WA: **Clara cells drive eosinophil accumulation in allergic asthma.** Eur Respir J 2012, **39**(2):429-438.

14. Takemasa A, Ishii Y, Fukuda T: **A neutrophil elastase inhibitor prevents bleomycin-induced pulmonary fibrosis in mice.** Eur Respir J 2012, **40**(6):1475-1482.

15. Toledo AC, Magalhaes RM, Hizume DC, Vieira RP, Biselli PJ, Moriya HT, Mauad T, Lopes FD, Martins MA: **Aerobic exercise attenuates pulmonary injury induced by exposure to cigarette smoke.** Eur Respir J 2012, **39**(2):254-264.

16. Wong RS, Larcombe AN, Fernandes LB, Zosky GR, Noble PB: **The mechanism of deep inspiration-induced bronchoprotection: evidence from a mouse model.** Eur Respir J 2012, **40**(4):982-989.

**23. Journal of Heart and Lung Transplantation**

1. Beazley KE, Zhang T, Lima F, Pozharskaya T, Niger C, Tzitzikov E, Azimzadeh AM, Nurminskaya M: **Implication for transglutaminase 2-mediated activation of beta-catenin signaling in neointimal vascular smooth muscle cells in chronic cardiac allograft rejection.** J Heart Lung Transplant 2012, **31**(9):1009-1017.

2. Floerchinger B, Ge X, Lee YL, Jurisch A, Padera RF, Schmid C, Tullius SG: **Graft-specific immune cells communicate inflammatory immune responses after brain death.** J Heart Lung Transplant 2012, **31**(12):1293-1300.

3. Gareau AJ, Nashan B, Hirsch GM, Lee TD: **Cyclosporine immunosuppression does not prevent the production of donor-specific antibody capable of mediating allograft vasculopathy.** J Heart Lung Transplant 2012, **31**(8):874-880.

4. He W, Ye L, Li S, Liu H, Wu B, Wang Q, Fu X, Han W, Chen Z: **Construction of vascularized cardiac tissue from genetically modified mouse embryonic stem cells.** J Heart Lung Transplant 2012, **31**(2):204-212.

5. Jungraithmayr W, Draenert A, Marquardt K, Weder W: **Ultrastructural changes in acute lung allograft rejection: novel insights from an animal study.** J Heart Lung Transplant 2012, **31**(1):94-100.

6. Knudsen L, Boxler L, Muhlfeld C, Schaefer IM, Becker L, Bussinger C, von Stietencron I, Madershahian N, Richter J, Wahlers T, Wittwer T, Ochs M: **Lung preservation in experimental ischemia/reperfusion injury and lung transplantation: a comparison of natural and synthetic surfactants.** J Heart Lung Transplant 2012, **31**(1):85-93.

7. Lv X, Tan J, Liu D, Wu P, Cui X: **Intratracheal administration of p38alpha short-hairpin RNA plasmid ameliorates lung ischemia-reperfusion injury in rats.** J Heart Lung Transplant 2012, **31**(6):655-662.

8. Plenter RJ, Grazia TJ, Doan AN, Gill RG, Pietra BA: **CD4 T cells mediate cardiac xenograft rejection via host MHC Class II.** J Heart Lung Transplant 2012, **31**(9):1018-1024.

9. Ropponen JO, Syrjala SO, Hollmen M, Tuuminen R, Krebs R, Keranen MA, Vaali K, Nykanen AI, Lemstrom KB, Tikkanen JM: **Effect of simvastatin on development of obliterative airway disease: an experimental study.** J Heart Lung Transplant 2012, **31**(2):194-203.

10. Santana-Rodriguez N, Garcia-Herrera R, Clavo B, Llontop P, Ponce-Gonzalez MA, Villar J, Lopez-Garcia A, Fiuza MD, Rodriguez-Bermejo JC, Garcia-Castellano JM, Machin RP, Ruiz-Caballero JA, Brito Y, Fernandez-Perez L: **Searching for novel molecular targets of chronic rejection in an orthotopic experimental lung transplantation model.** J Heart Lung Transplant 2012, **31**(2):213-221.

11. Skelton TS, Tejpal N, Gong Y, Kubiak JZ, Kloc M, Ghobrial RM: **Allochimeric molecules and mechanisms in abrogation of cardiac allograft rejection.** J Heart Lung Transplant 2012, **31**(1):73-84.

12. Takenaka M, Subramanian V, Tiriveedhi V, Phelan D, Hachem R, Trulock E, Gelman AE, Patterson GA, Hoshinaga K, Mohanakumar T: **Complement activation is not required for obliterative airway disease induced by antibodies to major histocompatibility complex class I: Implications for chronic lung rejection.** J Heart Lung Transplant 2012, **31**(11):1214-1222.

13. van der Kaaij NP, Kluin J, Lachmann RA, den Bakker MA, Lambrecht BN, Lachmann B, de Bruin RW, Bogers AJ: **Alveolar preservation with high inflation pressure and intermediate oxygen concentration reduces ischemia-reperfusion injury of the lung.** J Heart Lung Transplant 2012, **31**(5):531-537.

14. Weber J, Tiriveedhi V, Takenaka M, Lu W, Hachem R, Trulock E, Patterson GA, Mohanakumar T: **Inhibition of renin angiotensin aldosterone system causes abrogation of obliterative airways disease through inhibition of tumor necrosis factor-alpha-dependant interleukin-17.** J Heart Lung Transplant 2012, **31**(4):419-426.

**24. Journal of Thoracic Oncology**

1. John T, Yanagawa N, Kohler D, Craddock KJ, Bandarchi-Chamkhaleh B, Pintilie M, Sykes J, To C, Li M, Panchal D, Chen W, Shepherd FA, Tsao MS: **Characterization of lymphomas developing in immunodeficient mice implanted with primary human non-small cell lung cancer.** J Thorac Oncol 2012, **7**(7):1101-1108.

2. Koizumi H, Yamada T, Takeuchi S, Nakagawa T, Kita K, Nakamura T, Matsumoto K, Suda K, Mitsudomi T, Yano S: **Hsp90 inhibition overcomes HGF-triggering resistance to EGFR-TKIs in EGFR-mutant lung cancer by decreasing client protein expression and angiogenesis.** J Thorac Oncol 2012, **7**(7):1078-1085.

3. Li X, Wan L, Geng J, Wu CL, Bai X: **Aldehyde dehydrogenase 1A1 possesses stem-like properties and predicts lung cancer patient outcome.** J Thorac Oncol 2012, **7**(8):1235-1245.

4. Siegfried JM, Gubish CT, Rothstein ME, Henry C, Stabile LP: **Combining the multitargeted tyrosine kinase inhibitor vandetanib with the antiestrogen fulvestrant enhances its antitumor effect in non-small cell lung cancer.** J Thorac Oncol 2012, **7**(3):485-495.

**25. American Journal of Respiratory Cell and Molecular Biology**

1. Aeffner F, Bratasz A, Flano E, Powell KA, Davis IC: **Postinfection A77-1726 treatment improves cardiopulmonary function in H1N1 influenza-infected mice.** Am J Respir Cell Mol Biol 2012, **47**(4):543-551.

2. Ahmad T, Kumar M, Mabalirajan U, Pattnaik B, Aggarwal S, Singh R, Singh S, Mukerji M, Ghosh B, Agrawal A: **Hypoxia response in asthma: differential modulation on inflammation and epithelial injury.** Am J Respir Cell Mol Biol 2012, **47**(1):1-10.

3. Anjum F, Joshi K, Grinkina N, Gowda S, Cutaia M, Wadgaonkar R: **Role of sphingomyelin synthesis in pulmonary endothelial cell cytoskeletal activation and endotoxin-induced lung injury.** Am J Respir Cell Mol Biol 2012, **47**(1):94-103.

4. Auten RL, Gilmour MI, Krantz QT, Potts EN, Mason SN, Foster WM: **Maternal diesel inhalation increases airway hyperreactivity in ozone-exposed offspring.** Am J Respir Cell Mol Biol 2012, **46**(4):454-460.

5. Bae HB, Zmijewski JW, Deshane JS, Zhi D, Thompson LC, Peterson CB, Chaplin DD, Abraham E: **Vitronectin inhibits neutrophil apoptosis through activation of integrin-associated signaling pathways.** Am J Respir Cell Mol Biol 2012, **46**(6):790-796.

6. Baek HA, Kim do S, Park HS, Jang KY, Kang MJ, Lee DG, Moon WS, Chae HJ, Chung MJ: **Involvement of endoplasmic reticulum stress in myofibroblastic differentiation of lung fibroblasts.** Am J Respir Cell Mol Biol 2012, **46**(6):731-739.

7. Banerjee A, Trivedi CM, Damera G, Jiang M, Jester W, Hoshi T, Epstein JA, Panettieri RA,Jr: **Trichostatin A abrogates airway constriction, but not inflammation, in murine and human asthma models.** Am J Respir Cell Mol Biol 2012, **46**(2):132-138.

8. Barbier D, Garcia-Verdugo I, Pothlichet J, Khazen R, Descamps D, Rousseau K, Thornton D, Si-Tahar M, Touqui L, Chignard M, Sallenave JM: **Influenza A induces the major secreted airway mucin MUC5AC in a protease-EGFR-extracellular regulated kinase-Sp1-dependent pathway.** Am J Respir Cell Mol Biol 2012, **47**(2):149-157.

9. Bhandari V, Choo-Wing R, Harijith A, Sun H, Syed MA, Homer RJ, Elias JA: **Increased hyperoxia-induced lung injury in nitric oxide synthase 2 null mice is mediated via angiopoietin 2.** Am J Respir Cell Mol Biol 2012, **46**(5):668-676.

10. Bogaard HJ, Mizuno S, Guignabert C, Al Hussaini AA, Farkas D, Ruiter G, Kraskauskas D, Fadel E, Allegood JC, Humbert M, Vonk Noordegraaf A, Spiegel S, Farkas L, Voelkel NF: **Copper dependence of angioproliferation in pulmonary arterial hypertension in rats and humans.** Am J Respir Cell Mol Biol 2012, **46**(5):582-591.

11. Bundesmann MM, Wagner TE, Chow YH, Altemeier WA, Steinbach T, Schnapp LM: **Role of urokinase plasminogen activator receptor-associated protein in mouse lung.** Am J Respir Cell Mol Biol 2012, **46**(2):233-239.

12. Burnight ER, Wang G, McCray PB,Jr, Sinn PL: **Transcriptional targeting in the airway using novel gene regulatory elements.** Am J Respir Cell Mol Biol 2012, **47**(2):227-233.

13. Cardini S, Dalli J, Fineschi S, Perretti M, Lungarella G, Lucattelli M: **Genetic ablation of the fpr1 gene confers protection from smoking-induced lung emphysema in mice.** Am J Respir Cell Mol Biol 2012, **47**(3):332-339.

14. Carlin CM, Celnik DF, Pak O, Wadsworth R, Peacock AJ, Welsh DJ: **Low-dose fluvastatin reverses the hypoxic pulmonary adventitial fibroblast phenotype in experimental pulmonary hypertension.** Am J Respir Cell Mol Biol 2012, **47**(2):140-148.

15. Carpenter TC, Schroeder W, Stenmark KR, Schmidt EP: **Eph-A2 promotes permeability and inflammatory responses to bleomycin-induced lung injury.** Am J Respir Cell Mol Biol 2012, **46**(1):40-47.

16. Chamoto K, Gibney BC, Lee GS, Lin M, Collings-Simpson D, Voswinckel R, Konerding MA, Tsuda A, Mentzer SJ: **CD34+ progenitor to endothelial cell transition in post-pneumonectomy angiogenesis.** Am J Respir Cell Mol Biol 2012, **46**(3):283-289.

17. Chand HS, Woldegiorgis Z, Schwalm K, McDonald J, Tesfaigzi Y: **Acute inflammation induces insulin-like growth factor-1 to mediate Bcl-2 and Muc5ac expression in airway epithelial cells.** Am J Respir Cell Mol Biol 2012, **47**(6):784-791.

18. Chen L, Acciani T, Le Cras T, Lutzko C, Perl AK: **Dynamic regulation of platelet-derived growth factor receptor alpha expression in alveolar fibroblasts during realveolarization.** Am J Respir Cell Mol Biol 2012, **47**(4):517-527.

19. Chu KH, Chiang BL: **Regulatory T cells induced by mucosal B cells alleviate allergic airway hypersensitivity.** Am J Respir Cell Mol Biol 2012, **46**(5):651-659.

20. Dai C, Yao X, Keeran KJ, Zywicke GJ, Qu X, Yu ZX, Dagur PK, McCoy JP, Remaley AT, Levine SJ: **Apolipoprotein A-I attenuates ovalbumin-induced neutrophilic airway inflammation via a granulocyte colony-stimulating factor-dependent mechanism.** Am J Respir Cell Mol Biol 2012, **47**(2):186-195.

21. D'Alessandro-Gabazza CN, Kobayashi T, Boveda-Ruiz D, Takagi T, Toda M, Gil-Bernabe P, Miyake Y, Yasukawa A, Matsuda Y, Suzuki N, Saito H, Yano Y, Fukuda A, Hasegawa T, Toyobuku H, Rennard SI, Wagner PD, Morser J, Takei Y, Taguchi O, Gabazza EC: **Development and preclinical efficacy of novel transforming growth factor-beta1 short interfering RNAs for pulmonary fibrosis.** Am J Respir Cell Mol Biol 2012, **46**(3):397-406.

22. Du L, Zhou J, Zhang J, Yan M, Gong L, Liu X, Chen M, Tao K, Luo N, Liu J: **Actin filament reorganization is a key step in lung inflammation induced by systemic inflammatory response syndrome.** Am J Respir Cell Mol Biol 2012, **47**(5):597-603.

23. Fanucchi MV, Bracher A, Doran SF, Squadrito GL, Fernandez S, Postlethwait EM, Bowen L, Matalon S: **Post-exposure antioxidant treatment in rats decreases airway hyperplasia and hyperreactivity due to chlorine inhalation.** Am J Respir Cell Mol Biol 2012, **46**(5):599-606.

24. Fitz LJ, DeClercq C, Brooks J, Kuang W, Bates B, Demers D, Winkler A, Nocka K, Jiao A, Greco RM, Mason LE, Fleming M, Quazi A, Wright J, Goldman S, Hubeau C, Williams CM: **Acidic mammalian chitinase is not a critical target for allergic airway disease.** Am J Respir Cell Mol Biol 2012, **46**(1):71-79.

25. Funke M, Zhao Z, Xu Y, Chun J, Tager AM: **The lysophosphatidic acid receptor LPA1 promotes epithelial cell apoptosis after lung injury.** Am J Respir Cell Mol Biol 2012, **46**(3):355-364.

26. Furuhashi K, Suda T, Hasegawa H, Suzuki Y, Hashimoto D, Enomoto N, Fujisawa T, Nakamura Y, Inui N, Shibata K, Nakamura H, Chida K: **Mouse lung CD103+ and CD11bhigh dendritic cells preferentially induce distinct CD4+ T-cell responses.** Am J Respir Cell Mol Biol 2012, **46**(2):165-172.

27. Gowdy KM, Nugent JL, Martinu T, Potts E, Snyder LD, Foster WM, Palmer SM: **Protective role of T-bet and Th1 cytokines in pulmonary graft-versus-host disease and peribronchiolar fibrosis.** Am J Respir Cell Mol Biol 2012, **46**(2):249-256.

28. Green DE, Murphy TC, Kang BY, Kleinhenz JM, Szyndralewiez C, Page P, Sutliff RL, Hart CM: **The Nox4 inhibitor GKT137831 attenuates hypoxia-induced pulmonary vascular cell proliferation.** Am J Respir Cell Mol Biol 2012, **47**(5):718-726.

29. Grinnell KL, Chichger H, Braza J, Duong H, Harrington EO: **Protection against LPS-induced pulmonary edema through the attenuation of protein tyrosine phosphatase-1B oxidation.** Am J Respir Cell Mol Biol 2012, **46**(5):623-632.

30. Groves AM, Gow AJ, Massa CB, Laskin JD, Laskin DL: **Prolonged injury and altered lung function after ozone inhalation in mice with chronic lung inflammation.** Am J Respir Cell Mol Biol 2012, **47**(6):776-783.

31. Guetta J, Klorin G, Tal R, Berger G, Ismael-Badarneh R, Bishara B, Sabo E, Abassi Z, Azzam ZS: **Vasopressin-2 receptor antagonist attenuates the ability of the lungs to clear edema in an experimental model.** Am J Respir Cell Mol Biol 2012, **47**(5):583-588.

32. Hacha J, Tomlinson K, Maertens L, Paulissen G, Rocks N, Foidart JM, Noel A, Palframan R, Gueders M, Cataldo DD: **Nebulized anti-IL-13 monoclonal antibody Fab' fragment reduces allergen-induced asthma.** Am J Respir Cell Mol Biol 2012, **47**(5):709-717.

33. Hogmalm A, Backstrom E, Bry M, Lappalainen U, Lukkarinen HP, Bry K: **Role of CXC chemokine receptor-2 in a murine model of bronchopulmonary dysplasia.** Am J Respir Cell Mol Biol 2012, **47**(6):746-758.

34. Hollenhorst MI, Lips KS, Weitz A, Krasteva G, Kummer W, Fronius M: **Evidence for functional atypical nicotinic receptors that activate K+-dependent Cl- secretion in mouse tracheal epithelium.** Am J Respir Cell Mol Biol 2012, **46**(1):106-114.

35. Howden R, Cho HY, Miller-DeGraff L, Walker C, Clark JA, Myers PH, Rouse DC, Kleeberger SR: **Cardiac physiologic and genetic predictors of hyperoxia-induced acute lung injury in mice.** Am J Respir Cell Mol Biol 2012, **46**(4):470-478.

36. Hristova M, Spiess PC, Kasahara DI, Randall MJ, Deng B, van der Vliet A: **The tobacco smoke component, acrolein, suppresses innate macrophage responses by direct alkylation of c-Jun N-terminal kinase.** Am J Respir Cell Mol Biol 2012, **46**(1):23-33.

37. Hsieh WY, Chou CC, Ho CC, Yu SL, Chen HY, Chou HY, Chen JJ, Chen HW, Yang PC: **Single-walled carbon nanotubes induce airway hyperreactivity and parenchymal injury in mice.** Am J Respir Cell Mol Biol 2012, **46**(2):257-267.

38. Huang WT, Vayalil PK, Miyata T, Hagood J, Liu RM: **Therapeutic value of small molecule inhibitor to plasminogen activator inhibitor-1 for lung fibrosis.** Am J Respir Cell Mol Biol 2012, **46**(1):87-95.

39. Huang Y, Kempen MB, Munck AB, Swagemakers S, Driegen S, Mahavadi P, Meijer D, van Ijcken W, van der Spek P, Grosveld F, Gunther A, Tibboel D, Rottier RJ: **Hypoxia-inducible factor 2alpha plays a critical role in the formation of alveoli and surfactant.** Am J Respir Cell Mol Biol 2012, **46**(2):224-232.

40. Hudock KM, Liu Y, Mei J, Marino RC, Hale JE, Dai N, Worthen GS: **Delayed resolution of lung inflammation in Il-1rn-/- mice reflects elevated IL-17A/granulocyte colony-stimulating factor expression.** Am J Respir Cell Mol Biol 2012, **47**(4):436-444.

41. Iken K, Liu K, Liu H, Bizargity P, Wang L, Hancock WW, Visner GA: **Indoleamine 2,3-dioxygenase and metabolites protect murine lung allografts and impair the calcium mobilization of T cells.** Am J Respir Cell Mol Biol 2012, **47**(4):405-416.

42. Ionescu LI, Alphonse RS, Arizmendi N, Morgan B, Abel M, Eaton F, Duszyk M, Vliagoftis H, Aprahamian TR, Walsh K, Thebaud B: **Airway delivery of soluble factors from plastic-adherent bone marrow cells prevents murine asthma.** Am J Respir Cell Mol Biol 2012, **46**(2):207-216.

43. Jiang D, Liang J, Guo R, Xie T, Kelly FL, Martinu T, Yang T, Lovgren AK, Chia J, Liu N, Jung Y, Palmer SM, Noble PW: **Long-term exposure of chemokine CXCL10 causes bronchiolitis-like inflammation.** Am J Respir Cell Mol Biol 2012, **46**(5):592-598.

44. Jiang D, Liang J, Guo R, Xie T, Kelly FL, Martinu T, Yang T, Lovgren AK, Chia J, Liu N, Jung Y, Palmer SM, Noble PW: **Long-term exposure of chemokine CXCL10 causes bronchiolitis-like inflammation.** Am J Respir Cell Mol Biol 2012, **46**(5):592-598.

45. Kim JK, Vinarsky V, Wain J, Zhao R, Jung K, Choi J, Lam A, Pardo-Saganta A, Breton S, Rajagopal J, Yun SH: **In vivo imaging of tracheal epithelial cells in mice during airway regeneration.** Am J Respir Cell Mol Biol 2012, **47**(6):864-868.

46. Kim SY, Lee JH, Huh JW, Kim HJ, Park MK, Ro JY, Oh YM, Lee SD, Lee YS: **Bortezomib alleviates experimental pulmonary arterial hypertension.** Am J Respir Cell Mol Biol 2012, **47**(5):698-708.

47. Kim TH, Chow YH, Gill SE, Schnapp LM: **Effect of insulin-like growth factor blockade on hyperoxia-induced lung injury.** Am J Respir Cell Mol Biol 2012, **47**(3):372-378.

48. Kordonowy LL, Burg E, Lenox CC, Gauthier LM, Petty JM, Antkowiak M, Palvinskaya T, Ubags N, Rincon M, Dixon AE, Vernooy JH, Fessler MB, Poynter ME, Suratt BT: **Obesity is associated with neutrophil dysfunction and attenuation of murine acute lung injury.** Am J Respir Cell Mol Biol 2012, **47**(1):120-127.

49. Krishnaswamy JK, Jirmo AC, Baru AM, Ebensen T, Guzman CA, Sparwasser T, Behrens GM: **Toll-like receptor-2 agonist-allergen coupling efficiently redirects Th2 cell responses and inhibits allergic airway eosinophilia.** Am J Respir Cell Mol Biol 2012, **47**(6):852-863.

50. Lagares D, Busnadiego O, Garcia-Fernandez RA, Lamas S, Rodriguez-Pascual F: **Adenoviral gene transfer of endothelin-1 in the lung induces pulmonary fibrosis through the activation of focal adhesion kinase.** Am J Respir Cell Mol Biol 2012, **47**(6):834-842.

51. Lai PS, Fresco JM, Pinilla MA, Macias AA, Brown RD, Englert JA, Hofmann O, Lederer JA, Hide W, Christiani DC, Cernadas M, Baron RM: **Chronic endotoxin exposure produces airflow obstruction and lung dendritic cell expansion.** Am J Respir Cell Mol Biol 2012, **47**(2):209-217.

52. Langen RC, Haegens A, Vernooy JH, Wouters EF, de Winther MP, Carlsen H, Steele C, Shoelson SE, Schols AM: **NF-kappaB activation is required for the transition of pulmonary inflammation to muscle atrophy.** Am J Respir Cell Mol Biol 2012, **47**(3):288-297.

53. Lapar DJ, Hajzus VA, Zhao Y, Lau CL, French BA, Kron IL, Sharma AK, Laubach VE: **Acute hyperglycemic exacerbation of lung ischemia-reperfusion injury is mediated by receptor for advanced glycation end-products signaling.** Am J Respir Cell Mol Biol 2012, **46**(3):299-305.

54. Lazrak A, Chen L, Jurkuvenaite A, Doran SF, Liu G, Li Q, Lancaster JR,Jr, Matalon S: **Regulation of alveolar epithelial Na+ channels by ERK1/2 in chlorine-breathing mice.** Am J Respir Cell Mol Biol 2012, **46**(3):342-354.

55. Lee SH, Eren M, Vaughan DE, Schleimer RP, Cho SH: **A plasminogen activator inhibitor-1 inhibitor reduces airway remodeling in a murine model of chronic asthma.** Am J Respir Cell Mol Biol 2012, **46**(6):842-846.

56. Leikauf GD, Pope-Varsalona H, Concel VJ, Liu P, Bein K, Berndt A, Martin TM, Ganguly K, Jang AS, Brant KA, Dopico RA,Jr, Upadhyay S, Di YP, Li Q, Hu Z, Vuga LJ, Medvedovic M, Kaminski N, You M, Alexander DC, McDunn JE, Prows DR, Knoell DL, Fabisiak JP: **Integrative assessment of chlorine-induced acute lung injury in mice.** Am J Respir Cell Mol Biol 2012, **47**(2):234-244.

57. Lembrechts R, Brouns I, Schnorbusch K, Pintelon I, Timmermans JP, Adriaensen D: **Neuroepithelial bodies as mechanotransducers in the intrapulmonary airway epithelium: involvement of TRPC5.** Am J Respir Cell Mol Biol 2012, **47**(3):315-323.

58. Li HH, Li Q, Liu P, Liu Y, Li J, Wasserloos K, Chao W, You M, Oury TD, Chhinder S, Hackam DJ, Billiar TR, Leikauf GD, Pitt BR, Zhang LM: **WNT1-inducible signaling pathway protein 1 contributes to ventilator-induced lung injury.** Am J Respir Cell Mol Biol 2012, **47**(4):528-535.

59. Li Y, Connolly M, Nagaraj C, Tang B, Balint Z, Popper H, Smolle-Juettner FM, Lindenmann J, Kwapiszewska G, Aaronson PI, Wohlkoenig C, Leithner K, Olschewski H, Olschewski A: **Peroxisome proliferator-activated receptor-beta/delta, the acute signaling factor in prostacyclin-induced pulmonary vasodilation.** Am J Respir Cell Mol Biol 2012, **46**(3):372-379.

60. Li Y, Linnoila RI: **Multidirectional differentiation of Achaete-Scute homologue-1-defined progenitors in lung development and injury repair.** Am J Respir Cell Mol Biol 2012, **47**(6):768-775.

61. Madala SK, Schmidt S, Davidson C, Ikegami M, Wert S, Hardie WD: **MEK-ERK pathway modulation ameliorates pulmonary fibrosis associated with epidermal growth factor receptor activation.** Am J Respir Cell Mol Biol 2012, **46**(3):380-388.

62. Makena PS, Gorantla VK, Ghosh MC, Bezawada L, Kandasamy K, Balazs L, Luellen CL, Thompson KE, Parthasarathi K, Ichijo H, Waters CM, Sinclair SE: **Deletion of apoptosis signal-regulating kinase-1 prevents ventilator-induced lung injury in mice.** Am J Respir Cell Mol Biol 2012, **46**(4):461-469.

63. Makino F, Ito J, Abe Y, Harada N, Kamachi F, Yagita H, Takahashi K, Okumura K, Akiba H: **Blockade of CD70-CD27 interaction inhibits induction of allergic lung inflammation in mice.** Am J Respir Cell Mol Biol 2012, **47**(3):298-305.

64. Manzo ND, Foster WM, Stripp BR: **Amphiregulin-dependent mucous cell metaplasia in a model of nonallergic lung injury.** Am J Respir Cell Mol Biol 2012, **47**(3):349-357.

65. Matsumoto K, Asai Y, Fukuyama S, Kan-O K, Matsunaga Y, Noda N, Kitajima H, Tanaka K, Nakanishi Y, Inoue H: **IL-6 induced by double-stranded RNA augments allergic inflammation via suppression of Foxp3+ T-cell/IL-10 axis.** Am J Respir Cell Mol Biol 2012, **46**(6):740-747.

66. Medina JL, Coalson JJ, Brooks EG, Winter VT, Chaparro A, Principe MF, Kannan TR, Baseman JB, Dube PH: **Mycoplasma pneumoniae CARDS toxin induces pulmonary eosinophilic and lymphocytic inflammation.** Am J Respir Cell Mol Biol 2012, **46**(6):815-822.

67. Mekontso Dessap A, Voiriot G, Zhou T, Marcos E, Dudek SM, Jacobson JR, Machado R, Adnot S, Brochard L, Maitre B, Garcia JG: **Conflicting physiological and genomic cardiopulmonary effects of recruitment maneuvers in murine acute lung injury.** Am J Respir Cell Mol Biol 2012, **46**(4):541-550.

68. Mizuno S, Farkas L, Al Husseini A, Farkas D, Gomez-Arroyo J, Kraskauskas D, Nicolls MR, Cool CD, Bogaard HJ, Voelkel NF: **Severe pulmonary arterial hypertension induced by SU5416 and ovalbumin immunization.** Am J Respir Cell Mol Biol 2012, **47**(5):679-687.

69. Mundhenk L, Johannesson B, Anagnostopoulou P, Braun J, Bothe MK, Schultz C, Mall MA, Gruber AD: **mCLCA3 does not contribute to calcium-activated chloride conductance in murine airways.** Am J Respir Cell Mol Biol 2012, **47**(1):87-93.

70. Munitz A, Cole ET, Karo-Atar D, Finkelman FD, Rothenberg ME: **Resistin-like molecule-alpha regulates IL-13-induced chemokine production but not allergen-induced airway responses.** Am J Respir Cell Mol Biol 2012, **46**(5):703-713.

71. Nakav S, Cohen S, Feigelson SW, Bialik S, Shoseyov D, Kimchi A, Alon R: **Tumor suppressor death-associated protein kinase attenuates inflammatory responses in the lung.** Am J Respir Cell Mol Biol 2012, **46**(3):313-322.

72. Ohle SJ, Anandaiah A, Fabian AJ, Fine A, Kotton DN: **Maintenance and repair of the lung endothelium does not involve contributions from marrow-derived endothelial precursor cells.** Am J Respir Cell Mol Biol 2012, **47**(1):11-19.

73. Oikonomou N, Mouratis MA, Tzouvelekis A, Kaffe E, Valavanis C, Vilaras G, Karameris A, Prestwich GD, Bouros D, Aidinis V: **Pulmonary autotaxin expression contributes to the pathogenesis of pulmonary fibrosis.** Am J Respir Cell Mol Biol 2012, **47**(5):566-574.

74. Plant PJ, North ML, Ward A, Ward M, Khanna N, Correa J, Scott JA, Batt J: **Hypertrophic airway smooth muscle mass correlates with increased airway responsiveness in a murine model of asthma.** Am J Respir Cell Mol Biol 2012, **46**(4):532-540.

75. Poole JA, Gleason AM, Bauer C, West WW, Alexis N, van Rooijen N, Reynolds SJ, Romberger DJ, Kielian TL: **CD11c(+)/CD11b(+) cells are critical for organic dust-elicited murine lung inflammation.** Am J Respir Cell Mol Biol 2012, **47**(5):652-659.

76. Pritchard KA,Jr, Feroah TR, Nandedkar SD, Holzhauer SL, Hutchins W, Schulte ML, Strunk RC, Debaun MR, Hillery CA: **Effects of experimental asthma on inflammation and lung mechanics in sickle cell mice.** Am J Respir Cell Mol Biol 2012, **46**(3):389-396.

77. Riffo-Vasquez Y, Coates AR, Page CP, Spina D: **Mycobacterium tuberculosis chaperonin 60.1 inhibits leukocyte diapedesis in a murine model of allergic lung inflammation.** Am J Respir Cell Mol Biol 2012, **47**(2):245-252.

78. Roberson EC, Tully JE, Guala AS, Reiss JN, Godburn KE, Pociask DA, Alcorn JF, Riches DW, Dienz O, Janssen-Heininger YM, Anathy V: **Influenza induces endoplasmic reticulum stress, caspase-12-dependent apoptosis, and c-Jun N-terminal kinase-mediated transforming growth factor-beta release in lung epithelial cells.** Am J Respir Cell Mol Biol 2012, **46**(5):573-581.

79. Robertson JA, Sauer D, Gold JA, Nonas SA: **The role of cyclooxygenase-2 in mechanical ventilation-induced lung injury.** Am J Respir Cell Mol Biol 2012, **47**(3):387-394.

80. Rzepka JP, Haick AK, Miura TA: **Virus-infected alveolar epithelial cells direct neutrophil chemotaxis and inhibit their apoptosis.** Am J Respir Cell Mol Biol 2012, **46**(6):833-841.

81. Sato A, Xu Y, Whitsett JA, Ikegami M: **CCAAT/enhancer binding protein-alpha regulates the protease/antiprotease balance required for bronchiolar epithelium regeneration.** Am J Respir Cell Mol Biol 2012, **47**(4):454-463.

82. Schroeder BW, Verhaeghe C, Park SW, Nguyenvu LT, Huang X, Zhen G, Erle DJ: **AGR2 is induced in asthma and promotes allergen-induced mucin overproduction.** Am J Respir Cell Mol Biol 2012, **47**(2):178-185.

83. Shin YS, Takeda K, Shiraishi Y, Jia Y, Wang M, Jackson L, Wright AD, Carter L, Robinson J, Hicken E, Gelfand EW: **Inhibition of Pim1 kinase activation attenuates allergen-induced airway hyperresponsiveness and inflammation.** Am J Respir Cell Mol Biol 2012, **46**(4):488-497.

84. Shivshankar P, Brampton C, Miyasato S, Kasper M, Thannickal VJ, Le Saux CJ: **Caveolin-1 deficiency protects from pulmonary fibrosis by modulating epithelial cell senescence in mice.** Am J Respir Cell Mol Biol 2012, **47**(1):28-36.

85. Skelly JR, Edge D, Shortt CM, Jones JF, Bradford A, O'Halloran KD: **Tempol ameliorates pharyngeal dilator muscle dysfunction in a rodent model of chronic intermittent hypoxia.** Am J Respir Cell Mol Biol 2012, **46**(2):139-148.

86. Smith RW, Hicks DA, Reynolds SD: **Roles for beta-catenin and doxycycline in the regulation of respiratory epithelial cell frequency and function.** Am J Respir Cell Mol Biol 2012, **46**(1):115-124.

87. Stogsdill JA, Stogsdill MP, Porter JL, Hancock JM, Robinson AB, Reynolds PR: **Embryonic overexpression of receptors for advanced glycation end-products by alveolar epithelium induces an imbalance between proliferation and apoptosis.** Am J Respir Cell Mol Biol 2012, **47**(1):60-66.

88. Stolley JM, Gong D, Farley K, Zhao P, Cooley J, Crouch EC, Benarafa C, Remold-O'Donnell E: **Increased surfactant protein D fails to improve bacterial clearance and inflammation in serpinB1-/- mice.** Am J Respir Cell Mol Biol 2012, **47**(6):792-799.

89. Sun X, Singleton PA, Letsiou E, Zhao J, Belvitch P, Sammani S, Chiang ET, Moreno-Vinasco L, Wade MS, Zhou T, Liu B, Parastatidis I, Thomson L, Ischiropoulos H, Natarajan V, Jacobson JR, Machado RF, Dudek SM, Garcia JG: **Sphingosine-1-phosphate receptor-3 is a novel biomarker in acute lung injury.** Am J Respir Cell Mol Biol 2012, **47**(5):628-636.

90. Suresh MV, Wagner MC, Rosania GR, Stringer KA, Min KA, Risler L, Shen DD, Georges GE, Reddy AT, Parkkinen J, Reddy RC: **Pulmonary administration of a water-soluble curcumin complex reduces severity of acute lung injury.** Am J Respir Cell Mol Biol 2012, **47**(3):280-287.

91. Suresh MV, Yu B, Machado-Aranda D, Bender MD, Ochoa-Frongia L, Helinski JD, Davidson BA, Knight PR, Hogaboam CM, Moore BB, Raghavendran K: **Role of macrophage chemoattractant protein-1 in acute inflammation after lung contusion.** Am J Respir Cell Mol Biol 2012, **46**(6):797-806.

92. Suzuki Y, Suda T, Furuhashi K, Shibata K, Hashimoto D, Enomto N, Fujisawa T, Nakamura Y, Inui N, Nakamura H, Chida K: **Mouse CD11bhigh lung dendritic cells have more potent capability to induce IgA than CD103+ lung dendritic cells in vitro.** Am J Respir Cell Mol Biol 2012, **46**(6):773-780.

93. Swain SD, Meissner NN, Siemsen DW, McInnerney K, Harmsen AG: **Pneumocystis elicits a STAT6-dependent, strain-specific innate immune response and airway hyperresponsiveness.** Am J Respir Cell Mol Biol 2012, **46**(3):290-298.

94. Tanaka A, Jin Y, Lee SJ, Zhang M, Kim HP, Stolz DB, Ryter SW, Choi AM: **Hyperoxia-induced LC3B interacts with the Fas apoptotic pathway in epithelial cell death.** Am J Respir Cell Mol Biol 2012, **46**(4):507-514.

95. Tanino Y, Chang MY, Wang X, Gill SE, Skerrett S, McGuire JK, Sato S, Nikaido T, Kojima T, Munakata M, Mongovin S, Parks WC, Martin TR, Wight TN, Frevert CW: **Syndecan-4 regulates early neutrophil migration and pulmonary inflammation in response to lipopolysaccharide.** Am J Respir Cell Mol Biol 2012, **47**(2):196-202.

96. Thaikoottathil JV, Martin RJ, Di PY, Minor M, Case S, Zhang B, Zhang G, Huang H, Chu HW: **SPLUNC1 deficiency enhances airway eosinophilic inflammation in mice.** Am J Respir Cell Mol Biol 2012, **47**(2):253-260.

97. Torok JA, Brahmajothi MV, Zhu H, Tinch BT, Auten RL, McMahon TJ: **Transpulmonary flux of S-nitrosothiols and pulmonary vasodilation during nitric oxide inhalation: role of transport.** Am J Respir Cell Mol Biol 2012, **47**(1):37-43.

98. Torres-Gonzalez E, Bueno M, Tanaka A, Krug LT, Cheng DS, Polosukhin VV, Sorescu D, Lawson WE, Blackwell TS, Rojas M, Mora AL: **Role of endoplasmic reticulum stress in age-related susceptibility to lung fibrosis.** Am J Respir Cell Mol Biol 2012, **46**(6):748-756.

99. Tosiek MJ, Bader SR, Gruber AD, Buer J, Gereke M, Bruder D: **CD8(+) T cells responding to alveolar self-antigen lack CD25 expression and fail to precipitate autoimmunity.** Am J Respir Cell Mol Biol 2012, **47**(6):869-878.

100. Tulapurkar ME, Almutairy EA, Shah NG, He JR, Puche AC, Shapiro P, Singh IS, Hasday JD: **Febrile-range hyperthermia modifies endothelial and neutrophilic functions to promote extravasation.** Am J Respir Cell Mol Biol 2012, **46**(6):807-814.

101. Tully JE, Nolin JD, Guala AS, Hoffman SM, Roberson EC, Lahue KG, van der Velden J, Anathy V, Blackwell TS, Janssen-Heininger YM: **Cooperation between classical and alternative NF-kappaB pathways regulates proinflammatory responses in epithelial cells.** Am J Respir Cell Mol Biol 2012, **47**(4):497-508.

102. Varisco BM, Ambalavanan N, Whitsett JA, Hagood JS: **Thy-1 signals through PPARgamma to promote lipofibroblast differentiation in the developing lung.** Am J Respir Cell Mol Biol 2012, **46**(6):765-772.

103. Vaz M, Reddy NM, Rajasekaran S, Reddy SP: **Genetic disruption of Fra-1 decreases susceptibility to endotoxin-induced acute lung injury and mortality in mice.** Am J Respir Cell Mol Biol 2012, **46**(1):55-62.

104. Venkatesan N, Siddiqui S, Jo T, Martin JG, Ludwig MS: **Allergen-induced airway remodeling in brown norway rats: structural and metabolic changes in glycosaminoglycans.** Am J Respir Cell Mol Biol 2012, **46**(1):96-105.

105. Wang L, Yang J, Guo L, Uyeminami D, Dong H, Hammock BD, Pinkerton KE: **Use of a soluble epoxide hydrolase inhibitor in smoke-induced chronic obstructive pulmonary disease.** Am J Respir Cell Mol Biol 2012, **46**(5):614-622.

106. Wang T, Lang GD, Moreno-Vinasco L, Huang Y, Goonewardena SN, Peng YJ, Svensson EC, Natarajan V, Lang RM, Linares JD, Breysse PN, Geyh AS, Samet JM, Lussier YA, Dudley S, Prabhakar NR, Garcia JG: **Particulate matter induces cardiac arrhythmias via dysregulation of carotid body sensitivity and cardiac sodium channels.** Am J Respir Cell Mol Biol 2012, **46**(4):524-531.

107. Wilkosz S, Edwards LA, Bielsa S, Hyams C, Taylor A, Davies RJ, Laurent GJ, Chambers RC, Brown JS, Lee YC: **Characterization of a new mouse model of empyema and the mechanisms of pleural invasion by Streptococcus pneumoniae.** Am J Respir Cell Mol Biol 2012, **46**(2):180-187.

108. Williams L, Tucker TA, Koenig K, Allen T, Rao LV, Pendurthi U, Idell S: **Tissue factor pathway inhibitor attenuates the progression of malignant pleural mesothelioma in nude mice.** Am J Respir Cell Mol Biol 2012, **46**(2):173-179.

109. Wong MH, Chapin OC, Johnson MD: **LPS-stimulated cytokine production in type I cells is modulated by the renin-angiotensin system.** Am J Respir Cell Mol Biol 2012, **46**(5):641-650.

110. Wongtrakool C, Wang N, Hyde DM, Roman J, Spindel ER: **Prenatal nicotine exposure alters lung function and airway geometry through alpha7 nicotinic receptors.** Am J Respir Cell Mol Biol 2012, **46**(5):695-702.

111. Xiao R, Perveen Z, Paulsen D, Rouse R, Ambalavanan N, Kearney M, Penn AL: **In utero exposure to second-hand smoke aggravates adult responses to irritants: adult second-hand smoke.** Am J Respir Cell Mol Biol 2012, **47**(6):843-851.

112. Yamabayashi C, Koya T, Kagamu H, Kawakami H, Kimura Y, Furukawa T, Sakagami T, Hasegawa T, Sakai Y, Matsumoto K, Nakayama M, Gelfand EW, Suzuki E, Narita I: **A novel prostacyclin agonist protects against airway hyperresponsiveness and remodeling in mice.** Am J Respir Cell Mol Biol 2012, **47**(2):170-177.

113. Yang HH, Hou CC, Lin MT, Chang CP: **Attenuating heat-induced acute lung inflammation and injury by dextromethorphan in rats.** Am J Respir Cell Mol Biol 2012, **46**(3):407-413.

**26. Respiratory Research**

1. Baay-Guzman GJ, Bebenek IG, Zeidler M, Hernandez-Pando R, Vega MI, Garcia-Zepeda EA, Antonio-Andres G, Bonavida B, Riedl M, Kleerup E, Tashkin DP, Hankinson O, Huerta-Yepez S: **HIF-1 expression is associated with CCL2 chemokine expression in airway inflammatory cells: implications in allergic airway inflammation.** Respir Res 2012, **13**:60-9921-13-60.

2. Benson HL, Suzuki H, Lott J, Fisher AJ, Walline C, Heidler KM, Brutkiewicz R, Blum JS, Wilkes DS: **Donor lung derived myeloid and plasmacytoid dendritic cells differentially regulate T cell proliferation and cytokine production.** Respir Res 2012, **13**:25-9921-13-25.

3. Botelho FM, Nikota JK, Bauer CM, Morissette MC, Iwakura Y, Kolbeck R, Finch D, Humbles AA, Stampfli MR: **Cigarette smoke-induced accumulation of lung dendritic cells is interleukin-1alpha-dependent in mice.** Respir Res 2012, **13**:81-9921-13-81.

4. Deng W, Li CY, Tong J, Zhang W, Wang DX: **Regulation of ENaC-mediated alveolar fluid clearance by insulin via PI3K/Akt pathway in LPS-induced acute lung injury.** Respir Res 2012, **13**:29-9921-13-29.

5. Duan Y, Learoyd J, Meliton AY, Leff AR, Zhu X: **Inhibition of Pyk2 blocks lung inflammation and injury in a mouse model of acute lung injury.** Respir Res 2012, **13**:4-9921-13-4.

6. Feng JT, Wu XM, Li XZ, Zou YQ, Qin L, Hu CP: **Transformation of adrenal medullary chromaffin cells increases asthmatic susceptibility in pups from allergen-sensitized rats.** Respir Res 2012, **13**:99-9921-13-99.

7. Gil S, Farnand AW, Altemeier WA, Gill SE, Kurdowska A, Krupa A, Florence JM, Matute-Bello G: **Fas-deficient mice have impaired alveolar neutrophil recruitment and decreased expression of anti-KC autoantibody:KC complexes in a model of acute lung injury.** Respir Res 2012, **13**:91-9921-13-91.

8. Hackstein H, Wachtendorf A, Kranz S, Lohmeyer J, Bein G, Baal N: **Heterogeneity of respiratory dendritic cell subsets and lymphocyte populations in inbred mouse strains.** Respir Res 2012, **13**:94-9921-13-94.

9. Hardaker L, Bahra P, de Billy BC, Freeman M, Kupfer N, Wyss D, Trifilieff A: **The ion channel transient receptor potential melastatin-2 does not play a role in inflammatory mouse models of chronic obstructive pulmonary diseases.** Respir Res 2012, **13**:30-9921-13-30.

10. Jiang Z, Fehrenbach ML, Ravaioli G, Kokalari B, Redai IG, Sheardown SA, Wilson S, Macphee C, Haczku A: **The effect of lipoprotein-associated phospholipase A2 deficiency on pulmonary allergic responses in Aspergillus fumigatus sensitized mice.** Respir Res 2012, **13**:100-9921-13-100.

11. Ketabchi F, Ghofrani HA, Schermuly RT, Seeger W, Grimminger F, Egemnazarov B, Shid-Moosavi SM, Dehghani GA, Weissmann N, Sommer N: **Effects of hypercapnia and NO synthase inhibition in sustained hypoxic pulmonary vasoconstriction.** Respir Res 2012, **13**:7-9921-13-7.

12. Kinjo T, Tomaru K, Haines DC, Klinman DM: **The counter regulatory response induced by CpG oligonucleotides prevents bleomycin induced pneumopathy.** Respir Res 2012, **13**:47-9921-13-47.

13. Liao Z, Dong J, Wu W, Yang T, Wang T, Guo L, Chen L, Xu D, Wen F: **Resolvin D1 attenuates inflammation in lipopolysaccharide-induced acute lung injury through a process involving the PPARgamma/NF-kappaB pathway.** Respir Res 2012, **13**:110-9921-13-110.

14. Madala SK, Edukulla R, Davis KR, Schmidt S, Davidson C, Kitzmiller JA, Hardie WD, Korfhagen TR: **Resistin-like molecule alpha1 (Fizz1) recruits lung dendritic cells without causing pulmonary fibrosis.** Respir Res 2012, **13**:51-9921-13-51.

15. Manoli SE, Smith LA, Vyhlidal CA, An CH, Porrata Y, Cardoso WV, Baron RM, Haley KJ: **Maternal smoking and the retinoid pathway in the developing lung.** Respir Res 2012, **13**:42-9921-13-42.

16. Min JH, Codipilly CN, Nasim S, Miller EJ, Ahmed MN: **Synergistic protection against hyperoxia-induced lung injury by neutrophils blockade and EC-SOD overexpression.** Respir Res 2012, **13**:58-9921-13-58.

17. Musah S, Chen J, Hoyle GW: **Repair of tracheal epithelium by basal cells after chlorine-induced injury.** Respir Res 2012, **13**:107-9921-13-107.

18. Pan C, Wang J, Liu W, Liu L, Jing L, Yang Y, Qiu H: **Low tidal volume protects pulmonary vasomotor function from "second-hit" injury in acute lung injury rats.** Respir Res 2012, **13**:77-9921-13-77.

19. Patterson CM, Morrison RL, D'Souza A, Teng XS, Happel KI: **Inhaled fluticasone propionate impairs pulmonary clearance of Klebsiella pneumoniae in mice.** Respir Res 2012, **13**:40-9921-13-40.

20. Petak F, Albu G, Lele E, Beghetti M, Habre W: **Prevention of airway hyperresponsiveness induced by left ventricular dysfunction in rats.** Respir Res 2012, **13**:114-9921-13-114.

21. Shimodaira K, Okubo Y, Ochiai E, Nakayama H, Katano H, Wakayama M, Shinozaki M, Ishiwatari T, Sasai D, Tochigi N, Nemoto T, Saji T, Kamei K, Shibuya K: **Gene expression analysis of a murine model with pulmonary vascular remodeling compared to end-stage IPAH lungs.** Respir Res 2012, **13**:103-9921-13-103.

22. Smeding L, Plotz FB, Lamberts RR, van der Laarse WJ, Kneyber MC, Groeneveld AB: **Mechanical ventilation with high tidal volumes attenuates myocardial dysfunction by decreasing cardiac edema in a rat model of LPS-induced peritonitis.** Respir Res 2012, **13**:23-9921-13-23.

23. Sopi RB, Zaidi SI, Mladenov M, Sahiti H, Istrefi Z, Gjorgoski I, Lajci A, Jakupaj M: **L-citrulline supplementation reverses the impaired airway relaxation in neonatal rats exposed to hyperoxia.** Respir Res 2012, **13**:68-9921-13-68.

24. Tankersley CG, Moldobaeva A, Wagner EM: **Strain variation in response to lung ischemia: role of MMP-12.** Respir Res 2012, **13**:93-9921-13-93.

25. Willems CH, Urlichs F, Seidenspinner S, Kunzmann S, Speer CP, Kramer BW: **Poractant alfa (Curosurf(R)) increases phagocytosis of apoptotic neutrophils by alveolar macrophages in vivo.** Respir Res 2012, **13**:17-9921-13-17.

26. You K, Xu X, Fu J, Xu S, Yue X, Yu Z, Xue X: **Hyperoxia disrupts pulmonary epithelial barrier in newborn rats via the deterioration of occludin and ZO-1.** Respir Res 2012, **13**:36-9921-13-36.

27. Zeki AA, Thai P, Kenyon NJ, Wu R: **Differential effects of simvastatin on IL-13-induced cytokine gene expression in primary mouse tracheal epithelial cells.** Respir Res 2012, **13**:38.

**27. Journal of Thoracic and Cardiovascular Surgery**

1. Apitz C, Honjo O, Humpl T, Li J, Assad RS, Cho MY, Hong J, Friedberg MK, Redington AN: **Biventricular structural and functional responses to aortic constriction in a rabbit model of chronic right ventricular pressure overload.** J Thorac Cardiovasc Surg 2012, **144**(6):1494-1501.

2. Gallo A, Saad A, Ali R, Dardik A, Tellides G, Geirsson A: **Circulating interferon-gamma-inducible Cys-X-Cys chemokine receptor 3 ligands are elevated in humans with aortic aneurysms and Cys-X-Cys chemokine receptor 3 is necessary for aneurysm formation in mice.** J Thorac Cardiovasc Surg 2012, **143**(3):704-710.

3. Hibino N, Duncan DR, Nalbandian A, Yi T, Qyang Y, Shinoka T, Breuer CK: **Evaluation of the use of an induced puripotent stem cell sheet for the construction of tissue-engineered vascular grafts.** J Thorac Cardiovasc Surg 2012, **143**(3):696-703.

4. Neef K, Choi YH, Perumal Srinivasan S, Treskes P, Cowan DB, Stamm C, Rubach M, Adelmann R, Wittwer T, Wahlers T: **Mechanical preconditioning enables electrophysiologic coupling of skeletal myoblast cells to myocardium.** J Thorac Cardiovasc Surg 2012, **144**(5):1176-1184.e1.

5. Novotny L, Crha M, Rauser P, Hep A, Misik J, Necas A, Vondrys D: **Novel biodegradable polydioxanone stents in a rabbit airway model.** J Thorac Cardiovasc Surg 2012, **143**(2):437-444.

6. Rungatscher A, Linardi D, Tessari M, Menon T, Luciani GB, Mazzucco A, Faggian G: **Levosimendan is superior to epinephrine in improving myocardial function after cardiopulmonary bypass with deep hypothermic circulatory arrest in rats.** J Thorac Cardiovasc Surg 2012, **143**(1):209-214.

7. Toba H, Sakiyama S, Kenzaki K, Kawakami Y, Uyama K, Bando Y, Tangoku A: **Implantation of fetal rat lung fragments into bleomycin-induced pulmonary fibrosis.** J Thorac Cardiovasc Surg 2012, **143**(6):1429-1435.

8. Wada H, Yoshida S, Suzuki H, Sakairi Y, Mizobuchi T, Komura D, Sato Y, Yokoi S, Yoshino I: **Transplantation of alveolar type II cells stimulates lung regeneration during compensatory lung growth in adult rats.** J Thorac Cardiovasc Surg 2012, **143**(3):711-719.e2.

9. Yu JA, Mauchley D, Li H, Meng X, Nemenoff RA, Fullerton DA, Weyant MJ: **Knockdown of secretory phospholipase A2 IIa reduces lung cancer growth in vitro and in vivo.** J Thorac Cardiovasc Surg 2012, **144**(5):1185-1191.

**28. American Journal of Physiology – Lung Cellular and Molecular Physiology**

1. Abdala-Valencia H, Berdnikovs S, McCary CA, Urick D, Mahadevia R, Marchese ME, Swartz K, Wright L, Mutlu GM, Cook-Mills JM: **Inhibition of allergic inflammation by supplementation with 5-hydroxytryptophan.** Am J Physiol Lung Cell Mol Physiol 2012, **303**(8):L642-60.

2. Abid S, Houssaini A, Chevarin C, Marcos E, Tissot CM, Gary-Bobo G, Wan F, Mouraret N, Amsellem V, Dubois-Rande JL, Hamon M, Adnot S: **Inhibition of gut- and lung-derived serotonin attenuates pulmonary hypertension in mice.** Am J Physiol Lung Cell Mol Physiol 2012, **303**(6):L500-8.

3. Agarwal AR, Zhao L, Sancheti H, Sundar IK, Rahman I, Cadenas E: **Short-term cigarette smoke exposure induces reversible changes in energy metabolism and cellular redox status independent of inflammatory responses in mouse lungs.** Am J Physiol Lung Cell Mol Physiol 2012, **303**(10):L889-98.

4. An CH, Wang XM, Lam HC, Ifedigbo E, Washko GR, Ryter SW, Choi AM: **TLR4 deficiency promotes autophagy during cigarette smoke-induced pulmonary emphysema.** Am J Physiol Lung Cell Mol Physiol 2012, **303**(9):L748-57.

5. An SS, Wang WC, Koziol-White CJ, Ahn K, Lee DY, Kurten RC, Panettieri RA,Jr, Liggett SB: **TAS2R activation promotes airway smooth muscle relaxation despite beta(2)-adrenergic receptor tachyphylaxis.** Am J Physiol Lung Cell Mol Physiol 2012, **303**(4):L304-11.

6. Andonegui G, Ni A, Leger C, Kelly MM, Wong JF, Jalloul A, Winston BW: **Sequential expression of IGF-IB followed by active TGF-beta1 induces synergistic pulmonary fibroproliferation in vivo.** Am J Physiol Lung Cell Mol Physiol 2012, **303**(9):L788-98.

7. Aravamudan B, VanOosten SK, Meuchel LW, Vohra P, Thompson M, Sieck GC, Prakash YS, Pabelick CM: **Caveolin-1 knockout mice exhibit airway hyperreactivity.** Am J Physiol Lung Cell Mol Physiol 2012, **303**(8):L669-81.

8. Badea CT, Guo X, Clark D, Johnston SM, Marshall CD, Piantadosi CA: **Dual-energy micro-CT of the rodent lung.** Am J Physiol Lung Cell Mol Physiol 2012, **302**(10):L1088-97.

9. Basic VT, Tadele E, Elmabsout AA, Yao H, Rahman I, Sirsjo A, Abdel-Halim SM: **Exposure to cigarette smoke induces overexpression of von Hippel-Lindau tumor suppressor in mouse skeletal muscle.** Am J Physiol Lung Cell Mol Physiol 2012, **303**(6):L519-27.

10. Bhandary YP, Shetty SK, Marudamuthu AS, Gyetko MR, Idell S, Gharaee-Kermani M, Shetty RS, Starcher BC, Shetty S: **Regulation of alveolar epithelial cell apoptosis and pulmonary fibrosis by coordinate expression of components of the fibrinolytic system.** Am J Physiol Lung Cell Mol Physiol 2012, **302**(5):L463-73.

11. Bhattacharya M, Su G, Su X, Oses-Prieto JA, Li JT, Huang X, Hernandez H, Atakilit A, Burlingame AL, Matthay MA, Sheppard D: **IQGAP1 is necessary for pulmonary vascular barrier protection in murine acute lung injury and pneumonia.** Am J Physiol Lung Cell Mol Physiol 2012, **303**(1):L12-9.

12. Birukova AA, Tian Y, Meliton A, Leff A, Wu T, Birukov KG: **Stimulation of Rho signaling by pathologic mechanical stretch is a "second hit" to Rho-independent lung injury induced by IL-6.** Am J Physiol Lung Cell Mol Physiol 2012, **302**(9):L965-75.

13. Bongard RD, Myers CR, Lindemer BJ, Baumgardt S, Gonzalez FJ, Merker MP: **Coenzyme Q(1) as a probe for mitochondrial complex I activity in the intact perfused hyperoxia-exposed wild-type and Nqo1-null mouse lung.** Am J Physiol Lung Cell Mol Physiol 2012, **302**(9):L949-58.

14. Brand JD, Ballinger CA, Tuggle KL, Fanucchi MV, Schwiebert LM, Postlethwait EM: **Site-specific dynamics of CD11b+ and CD103+ dendritic cell accumulations following ozone exposure.** Am J Physiol Lung Cell Mol Physiol 2012, **303**(12):L1079-86.

15. Brueggemann LI, Kakad PP, Love RB, Solway J, Dowell ML, Cribbs LL, Byron KL: **Kv7 potassium channels in airway smooth muscle cells: signal transduction intermediates and pharmacological targets for bronchodilator therapy.** Am J Physiol Lung Cell Mol Physiol 2012, **302**(1):L120-32.

16. Buczynski BW, Yee M, Paige Lawrence B, O'Reilly MA: **Lung development and the host response to influenza A virus are altered by different doses of neonatal oxygen in mice.** Am J Physiol Lung Cell Mol Physiol 2012, **302**(10):L1078-87.

17. Buehler PW, Baek JH, Lisk C, Connor I, Sullivan T, Kominsky D, Majka S, Stenmark KR, Nozik-Grayck E, Bonaventura J, Irwin DC: **Free hemoglobin induction of pulmonary vascular disease: evidence for an inflammatory mechanism.** Am J Physiol Lung Cell Mol Physiol 2012, **303**(4):L312-26.

18. Campos R, Shimizu MH, Volpini RA, de Braganca AC, Andrade L, Lopes FD, Olivo C, Canale D, Seguro AC: **N-acetylcysteine prevents pulmonary edema and acute kidney injury in rats with sepsis submitted to mechanical ventilation.** Am J Physiol Lung Cell Mol Physiol 2012, **302**(7):L640-50.

19. Carpe N, Mandeville I, Kho AT, Qiu W, Martin JG, Tantisira KG, Raby BA, Weiss ST, Kaplan F: **Maternal allergen exposure reprograms the developmental lung transcriptome in atopic and normoresponsive rat pups.** Am J Physiol Lung Cell Mol Physiol 2012, **303**(10):L899-911.

20. Chang CC, Chiu JJ, Chen SL, Huang HC, Chiu HF, Lin BH, Yang CY: **Activation of HGF/c-Met signaling by ultrafine carbon particles and its contribution to alveolar type II cell proliferation.** Am J Physiol Lung Cell Mol Physiol 2012, **302**(8):L755-63.

21. Chang YT, Ringman Uggla A, Osterholm C, Tran PK, Eklof AC, Lengquist M, Hedin U, Tran-Lundmark K, Frenckner B: **Antenatal imatinib treatment reduces pulmonary vascular remodeling in a rat model of congenital diaphragmatic hernia.** Am J Physiol Lung Cell Mol Physiol 2012, **302**(11):L1159-66.

22. Chen W, Sammani S, Mitra S, Ma SF, Garcia JG, Jacobson JR: **Critical role for integrin-beta4 in the attenuation of murine acute lung injury by simvastatin.** Am J Physiol Lung Cell Mol Physiol 2012, **303**(4):L279-85.

23. Chichger H, Grinnell KL, Casserly B, Chung CS, Braza J, Lomas-Neira J, Ayala A, Rounds S, Klinger JR, Harrington EO: **Genetic disruption of protein kinase Cdelta reduces endotoxin-induced lung injury.** Am J Physiol Lung Cell Mol Physiol 2012, **303**(10):L880-8.

24. Cho KA, Suh JW, Sohn JH, Park JW, Lee H, Kang JL, Woo SY, Cho YJ: **IL-33 induces Th17-mediated airway inflammation via mast cells in ovalbumin-challenged mice.** Am J Physiol Lung Cell Mol Physiol 2012, **302**(4):L429-40.

25. Christou H, Reslan OM, Mam V, Tanbe AF, Vitali SH, Touma M, Arons E, Mitsialis SA, Kourembanas S, Khalil RA: **Improved pulmonary vascular reactivity and decreased hypertrophic remodeling during nonhypercapnic acidosis in experimental pulmonary hypertension.** Am J Physiol Lung Cell Mol Physiol 2012, **302**(9):L875-90.

26. Cioffi DL, Pandey S, Alvarez DF, Cioffi EA: **Terminal sialic acids are an important determinant of pulmonary endothelial barrier integrity.** Am J Physiol Lung Cell Mol Physiol 2012, **302**(10):L1067-77.

27. Cornell TT, Fleszar A, McHugh W, Blatt NB, Le Vine AM, Shanley TP: **Mitogen-activated protein kinase phosphatase 2, MKP-2, regulates early inflammation in acute lung injury.** Am J Physiol Lung Cell Mol Physiol 2012, **303**(3):L251-8.

28. Cyphert JM, Allen IC, Church RJ, Latour AM, Snouwaert JN, Coffman TM, Koller BH: **Allergic inflammation induces a persistent mechanistic switch in thromboxane-mediated airway constriction in the mouse.** Am J Physiol Lung Cell Mol Physiol 2012, **302**(1):L140-51.

29. Dahan D, Ducret T, Quignard JF, Marthan R, Savineau JP, Esteve E: **Implication of the ryanodine receptor in TRPV4-induced calcium response in pulmonary arterial smooth muscle cells from normoxic and chronically hypoxic rats.** Am J Physiol Lung Cell Mol Physiol 2012, **303**(9):L824-33.

30. Davies RJ, Holmes AM, Deighton J, Long L, Yang X, Barker L, Walker C, Budd DC, Upton PD, Morrell NW: **BMP type II receptor deficiency confers resistance to growth inhibition by TGF-beta in pulmonary artery smooth muscle cells: role of proinflammatory cytokines.** Am J Physiol Lung Cell Mol Physiol 2012, **302**(6):L604-15.

31. de Visser YP, Walther FJ, Laghmani el H, Steendijk P, Middeldorp M, van der Laarse A, Wagenaar GT: **Phosphodiesterase 4 inhibition attenuates persistent heart and lung injury by neonatal hyperoxia in rats.** Am J Physiol Lung Cell Mol Physiol 2012, **302**(1):L56-67.

32. Deiuliis JA, Kampfrath T, Zhong J, Oghumu S, Maiseyeu A, Chen LC, Sun Q, Satoskar AR, Rajagopalan S: **Pulmonary T cell activation in response to chronic particulate air pollution.** Am J Physiol Lung Cell Mol Physiol 2012, **302**(4):L399-409.

33. Deppong CM, Xu J, Brody SL, Green JM: **Airway epithelial cells suppress T cell proliferation by an IFNgamma/STAT1/TGFbeta-dependent mechanism.** Am J Physiol Lung Cell Mol Physiol 2012, **302**(1):L167-73.

34. Doherty TA, Khorram N, Chang JE, Kim HK, Rosenthal P, Croft M, Broide DH: **STAT6 regulates natural helper cell proliferation during lung inflammation initiated by Alternaria.** Am J Physiol Lung Cell Mol Physiol 2012, **303**(7):L577-88.

35. Downs CA, Kriener LH, Yu L, Eaton DC, Jain L, Helms MN: **beta-Adrenergic agonists differentially regulate highly selective and nonselective epithelial sodium channels to promote alveolar fluid clearance in vivo.** Am J Physiol Lung Cell Mol Physiol 2012, **302**(11):L1167-78.

36. Dull RO, Cluff M, Kingston J, Hill D, Chen H, Hoehne S, Malleske DT, Kaur R: **Lung heparan sulfates modulate K(fc) during increased vascular pressure: evidence for glycocalyx-mediated mechanotransduction.** Am J Physiol Lung Cell Mol Physiol 2012, **302**(9):L816-28.

37. Emin MT, Sun L, Huertas A, Das S, Bhattacharya J, Bhattacharya S: **Platelets induce endothelial tissue factor expression in a mouse model of acid-induced lung injury.** Am J Physiol Lung Cell Mol Physiol 2012, **302**(11):L1209-20.

38. Fernandez-Gonzalez A, Alex Mitsialis S, Liu X, Kourembanas S: **Vasculoprotective effects of heme oxygenase-1 in a murine model of hyperoxia-induced bronchopulmonary dysplasia.** Am J Physiol Lung Cell Mol Physiol 2012, **302**(8):L775-84.

39. Fisher BJ, Kraskauskas D, Martin EJ, Farkas D, Wegelin JA, Brophy D, Ward KR, Voelkel NF, Fowler AA,3rd, Natarajan R: **Mechanisms of attenuation of abdominal sepsis induced acute lung injury by ascorbic acid.** Am J Physiol Lung Cell Mol Physiol 2012, **303**(1):L20-32.

40. Folkesson HG, Kuzenko SR, Lipson DA, Matthay MA, Simmons MA: **The adenosine 2A receptor agonist GW328267C improves lung function after acute lung injury in rats.** Am J Physiol Lung Cell Mol Physiol 2012, **303**(3):L259-71.

41. Fuchs B, Sjoberg L, Moller Westerberg C, Ekoff M, Swedin L, Dahlen SE, Adner M, Nilsson GP: **Mast cell engraftment of the peripheral lung enhances airway hyperresponsiveness in a mouse asthma model.** Am J Physiol Lung Cell Mol Physiol 2012, **303**(12):L1027-36.

42. Gairhe S, Bauer NN, Gebb SA, McMurtry IF: **Serotonin passes through myoendothelial gap junctions to promote pulmonary arterial smooth muscle cell differentiation.** Am J Physiol Lung Cell Mol Physiol 2012, **303**(9):L767-77.

43. Gallos G, Yim P, Chang S, Zhang Y, Xu D, Cook JM, Gerthoffer WT, Emala CW S: **Targeting the restricted alpha-subunit repertoire of airway smooth muscle GABAA receptors augments airway smooth muscle relaxation.** Am J Physiol Lung Cell Mol Physiol 2012, **302**(2):L248-56.

44. Ghosh MC, Makena PS, Gorantla V, Sinclair SE, Waters CM: **CXCR4 regulates migration of lung alveolar epithelial cells through activation of Rac1 and matrix metalloproteinase-2.** Am J Physiol Lung Cell Mol Physiol 2012, **302**(9):L846-56.

45. Godbole MM, Rao G, Paul BN, Mohan V, Singh P, Khare D, Babu S, Nath A, Singh PK, Tiwari S: **Prenatal iodine deficiency results in structurally and functionally immature lungs in neonatal rats.** Am J Physiol Lung Cell Mol Physiol 2012, **302**(10):L1037-43.

46. Goldklang M, Golovatch P, Zelonina T, Trischler J, Rabinowitz D, Lemaitre V, D'Armiento J: **Activation of the TLR4 signaling pathway and abnormal cholesterol efflux lead to emphysema in ApoE-deficient mice.** Am J Physiol Lung Cell Mol Physiol 2012, **302**(11):L1200-8.

47. Goodson P, Kumar A, Jain L, Kundu K, Murthy N, Koval M, Helms MN: **Nadph oxidase regulates alveolar epithelial sodium channel activity and lung fluid balance in vivo via O(-)(2) signaling.** Am J Physiol Lung Cell Mol Physiol 2012, **302**(4):L410-9.

48. Greenwood KK, Proper SP, Saini Y, Bramble LA, Jackson-Humbles DN, Wagner JG, Harkema JR, LaPres JJ: **Neonatal epithelial hypoxia inducible factor-1alpha expression regulates the response of the lung to experimental asthma.** Am J Physiol Lung Cell Mol Physiol 2012, **302**(5):L455-62.

49. Grubb BR, O'Neal WK, Ostrowski LE, Kreda SM, Button B, Boucher RC: **Transgenic hCFTR expression fails to correct beta-ENaC mouse lung disease.** Am J Physiol Lung Cell Mol Physiol 2012, **302**(2):L238-47.

50. Hilgendorff A, Parai K, Ertsey R, Juliana Rey-Parra G, Thebaud B, Tamosiuniene R, Jain N, Navarro EF, Starcher BC, Nicolls MR, Rabinovitch M, Bland RD: **Neonatal mice genetically modified to express the elastase inhibitor elafin are protected against the adverse effects of mechanical ventilation on lung growth.** Am J Physiol Lung Cell Mol Physiol 2012, **303**(3):L215-27.

51. Hobi N, Ravasio A, Haller T: **Interfacial stress affects rat alveolar type II cell signaling and gene expression.** Am J Physiol Lung Cell Mol Physiol 2012, **303**(2):L117-29.

52. Hoffman SM, Tully JE, Lahue KG, Anathy V, Nolin JD, Guala AS, van der Velden JL, Ho YS, Aliyeva M, Daphtary N, Lundblad LK, Irvin CG, Janssen-Heininger YM: **Genetic ablation of glutaredoxin-1 causes enhanced resolution of airways hyperresponsiveness and mucus metaplasia in mice with allergic airways disease.** Am J Physiol Lung Cell Mol Physiol 2012, **303**(6):L528-38.

53. Ionescu L, Byrne RN, van Haaften T, Vadivel A, Alphonse RS, Rey-Parra GJ, Weissmann G, Hall A, Eaton F, Thebaud B: **Stem cell conditioned medium improves acute lung injury in mice: in vivo evidence for stem cell paracrine action.** Am J Physiol Lung Cell Mol Physiol 2012, **303**(11):L967-77.

54. Iosef C, Alastalo TP, Hou Y, Chen C, Adams ES, Lyu SC, Cornfield DN, Alvira CM: **Inhibiting NF-kappaB in the developing lung disrupts angiogenesis and alveolarization.** Am J Physiol Lung Cell Mol Physiol 2012, **302**(10):L1023-36.

55. Johnson JA, Hemnes AR, Perrien DS, Schuster M, Robinson LJ, Gladson S, Loibner H, Bai S, Blackwell TR, Tada Y, Harral JW, Talati M, Lane KB, Fagan KA, West J: **Cytoskeletal defects in Bmpr2-associated pulmonary arterial hypertension.** Am J Physiol Lung Cell Mol Physiol 2012, **302**(5):L474-84.

56. Josephson MB, Jiao J, Xu S, Hu A, Paranjape C, Grunstein JS, Grumbach Y, Nino G, Kreiger PA, McDonough J, Grunstein MM: **IL-13-induced changes in endogenous glucocorticoid metabolism in the lung regulate the proasthmatic response.** Am J Physiol Lung Cell Mol Physiol 2012, **303**(5):L382-90.

57. Kang BN, Ha SG, Ge XN, Reza Hosseinkhani M, Bahaie NS, Greenberg Y, Blumenthal MN, Puri KD, Rao SP, Sriramarao P: **The p110delta subunit of PI3K regulates bone marrow-derived eosinophil trafficking and airway eosinophilia in allergen-challenged mice.** Am J Physiol Lung Cell Mol Physiol 2012, **302**(11):L1179-91.

58. Karmouty-Quintana H, Siddiqui S, Hassan M, Tsuchiya K, Risse PA, Xicota-Vila L, Marti-Solano M, Martin JG: **Treatment with a sphingosine-1-phosphate analog inhibits airway remodeling following repeated allergen exposure.** Am J Physiol Lung Cell Mol Physiol 2012, **302**(8):L736-45.

59. Kearns MT, Dalal S, Horstmann SA, Richens TR, Tanaka T, Doe JM, Boe DM, Voelkel NF, Taraseviciene-Stewart L, Janssen WJ, Lee CG, Elias JA, Bratton D, Tuder RM, Henson PM, Vandivier RW: **Vascular endothelial growth factor enhances macrophage clearance of apoptotic cells.** Am J Physiol Lung Cell Mol Physiol 2012, **302**(7):L711-8.

60. Kim SY, Lee JH, Kim HJ, Park MK, Huh JW, Ro JY, Oh YM, Lee SD, Lee YS: **Mesenchymal stem cell-conditioned media recovers lung fibroblasts from cigarette smoke-induced damage.** Am J Physiol Lung Cell Mol Physiol 2012, **302**(9):L891-908.

61. Konrad FM, Witte E, Vollmer I, Stark S, Reutershan J: **Adenosine receptor A2b on hematopoietic cells mediates LPS-induced migration of PMNs into the lung interstitium.** Am J Physiol Lung Cell Mol Physiol 2012, **303**(5):L425-38.

62. Konsavage WM, Zhang L, Wu Y, Shenberger JS: **Hyperoxia-induced activation of the integrated stress response in the newborn rat lung.** Am J Physiol Lung Cell Mol Physiol 2012, **302**(1):L27-35.

63. Lakshmi SP, Reddy AT, Naik MU, Naik UP, Reddy RC: **Effects of JAM-A deficiency or blocking antibodies on neutrophil migration and lung injury in a murine model of ALI.** Am J Physiol Lung Cell Mol Physiol 2012, **303**(9):L758-66.

64. Lath NR, Galambos C, Rocha AB, Malek M, Gittes GK, Potoka DA: **Defective pulmonary innervation and autonomic imbalance in congenital diaphragmatic hernia.** Am J Physiol Lung Cell Mol Physiol 2012, **302**(4):L390-8.

65. Leggett K, Maylor J, Undem C, Lai N, Lu W, Schweitzer K, King LS, Myers AC, Sylvester JT, Sidhaye V, Shimoda LA: **Hypoxia-induced migration in pulmonary arterial smooth muscle cells requires calcium-dependent upregulation of aquaporin 1.** Am J Physiol Lung Cell Mol Physiol 2012, **303**(4):L343-53.

66. Li H, Yuan X, Tang J, Zhang Y: **Lipopolysaccharide disrupts the directional persistence of alveolar myofibroblast migration through EGF receptor.** Am J Physiol Lung Cell Mol Physiol 2012, **302**(6):L569-79.

67. Liang J, Jung Y, Tighe RM, Xie T, Liu N, Leonard M, Gunn MD, Jiang D, Noble PW: **A macrophage subpopulation recruited by CC chemokine ligand-2 clears apoptotic cells in noninfectious lung injury.** Am J Physiol Lung Cell Mol Physiol 2012, **302**(9):L933-40.

68. Lieu TM, Myers AC, Meeker S, Undem BJ: **TRPV1 induction in airway vagal low-threshold mechanosensory neurons by allergen challenge and neurotrophic factors.** Am J Physiol Lung Cell Mol Physiol 2012, **302**(9):L941-8.

69. Liu D, Yan Z, Minshall RD, Schwartz DE, Chen Y, Hu G: **Activation of calpains mediates early lung neutrophilic inflammation in ventilator-induced lung injury.** Am J Physiol Lung Cell Mol Physiol 2012, **302**(4):L370-9.

70. Luke T, Maylor J, Undem C, Sylvester JT, Shimoda LA: **Kinase-dependent activation of voltage-gated Ca2+ channels by ET-1 in pulmonary arterial myocytes during chronic hypoxia.** Am J Physiol Lung Cell Mol Physiol 2012, **302**(10):L1128-39.

71. Manders E, de Man FS, Handoko ML, Westerhof N, van Hees HW, Stienen GJ, Vonk-Noordegraaf A, Ottenheijm CA: **Diaphragm weakness in pulmonary arterial hypertension: role of sarcomeric dysfunction.** Am J Physiol Lung Cell Mol Physiol 2012, **303**(12):L1070-8.

72. Manni ML, Epperly MW, Han W, Blackwell TS, Duncan SR, Piganelli JD, Oury TD: **Leukocyte-derived extracellular superoxide dismutase does not contribute to airspace EC-SOD after interstitial pulmonary injury.** Am J Physiol Lung Cell Mol Physiol 2012, **302**(1):L160-6.

73. Meyer ML, Potts-Kant EN, Ghio AJ, Fischer BM, Foster WM, Voynow JA: **NAD(P)H quinone oxidoreductase 1 regulates neutrophil elastase-induced mucous cell metaplasia.** Am J Physiol Lung Cell Mol Physiol 2012, **303**(3):L181-8.

74. Mir-Kasimov M, Sturrock A, McManus M, Paine R,3rd: **Effect of alveolar epithelial cell plasticity on the regulation of GM-CSF expression.** Am J Physiol Lung Cell Mol Physiol 2012, **302**(6):L504-11.

75. Nelson MP, Christmann BS, Dunaway CW, Morris A, Steele C: **Experimental Pneumocystis lung infection promotes M2a alveolar macrophage-derived MMP12 production.** Am J Physiol Lung Cell Mol Physiol 2012, **303**(5):L469-75.

76. Nishijima K, Shukunami K, Yoshinari H, Takahashi J, Maeda H, Takagi H, Kotsuji F: **Interactions among pulmonary surfactant, vernix caseosa, and intestinal enterocytes: intra-amniotic administration of fluorescently liposomes to pregnant rabbits.** Am J Physiol Lung Cell Mol Physiol 2012, **303**(3):L208-14.

77. Ohta H, Chiba S, Ebina M, Furuse M, Nukiwa T: **Altered expression of tight junction molecules in alveolar septa in lung injury and fibrosis.** Am J Physiol Lung Cell Mol Physiol 2012, **302**(2):L193-205.

78. Oishi H, Takano K, Tomita K, Takebe M, Yokoo H, Yamazaki M, Hattori Y: **Olprinone and colforsin daropate alleviate septic lung inflammation and apoptosis through CREB-independent activation of the Akt pathway.** Am J Physiol Lung Cell Mol Physiol 2012, **303**(2):L130-40.

79. Olave N, Nicola T, Zhang W, Bulger A, James M, Oparil S, Chen YF, Ambalavanan N: **Transforming growth factor-beta regulates endothelin-1 signaling in the newborn mouse lung during hypoxia exposure.** Am J Physiol Lung Cell Mol Physiol 2012, **302**(9):L857-65.

80. Oldenburg PJ, Poole JA, Sisson JH: **Alcohol reduces airway hyperresponsiveness (AHR) and allergic airway inflammation in mice.** Am J Physiol Lung Cell Mol Physiol 2012, **302**(3):L308-15.

81. Parthasarathi K: **Endothelial connexin43 mediates acid-induced increases in pulmonary microvascular permeability.** Am J Physiol Lung Cell Mol Physiol 2012, **303**(1):L33-42.

82. Pulkkinen V, Manson ML, Safholm J, Adner M, Dahlen SE: **The bitter taste receptor (TAS2R) agonists denatonium and chloroquine display distinct patterns of relaxation of the guinea pig trachea.** Am J Physiol Lung Cell Mol Physiol 2012, **303**(11):L956-66.

83. Qian F, Deng J, Gantner BN, Flavell RA, Dong C, Christman JW, Ye RD: **Map kinase phosphatase 5 protects against sepsis-induced acute lung injury.** Am J Physiol Lung Cell Mol Physiol 2012, **302**(9):L866-74.

84. Rancourt RC, Veress LA, Guo X, Jones TN, Hendry-Hofer TB, White CW: **Airway tissue factor-dependent coagulation activity in response to sulfur mustard analog 2-chloroethyl ethyl sulfide.** Am J Physiol Lung Cell Mol Physiol 2012, **302**(1):L82-92.

85. Reszka KJ, Xiong Y, Sallans L, Pasula R, Olakanmi O, Hassett DJ, Britigan BE: **Inactivation of the potent Pseudomonas aeruginosa cytotoxin pyocyanin by airway peroxidases and nitrite.** Am J Physiol Lung Cell Mol Physiol 2012, **302**(10):L1044-56.

86. Robinson AB, Johnson KD, Bennion BG, Reynolds PR: **RAGE signaling by alveolar macrophages influences tobacco smoke-induced inflammation.** Am J Physiol Lung Cell Mol Physiol 2012, **302**(11):L1192-9.

87. Roszell BR, Tao JQ, Yu KJ, Huang S, Bates SR: **Characterization of the Niemann-Pick C pathway in alveolar type II cells and lamellar bodies of the lung.** Am J Physiol Lung Cell Mol Physiol 2012, **302**(9):L919-32.

88. Ruvin Kumara VM, Wessling-Resnick M: **Olfactory ferric and ferrous iron absorption in iron-deficient rats.** Am J Physiol Lung Cell Mol Physiol 2012, **302**(12):L1280-6.

89. Ruwanpura SM, McLeod L, Miller A, Jones J, Vlahos R, Ramm G, Longano A, Bardin PG, Bozinovski S, Anderson GP, Jenkins BJ: **Deregulated Stat3 signaling dissociates pulmonary inflammation from emphysema in gp130 mutant mice.** Am J Physiol Lung Cell Mol Physiol 2012, **302**(7):L627-39.

90. Sakamoto K, Hashimoto N, Kondoh Y, Imaizumi K, Aoyama D, Kohnoh T, Kusunose M, Kimura M, Kawabe T, Taniguchi H, Hasegawa Y: **Differential modulation of surfactant protein D under acute and persistent hypoxia in acute lung injury.** Am J Physiol Lung Cell Mol Physiol 2012, **303**(1):L43-53.

91. Schwingshackl A, Teng B, Ghosh M, West AN, Makena P, Gorantla V, Sinclair SE, Waters CM: **Regulation and function of the two-pore-domain (K2P) potassium channel Trek-1 in alveolar epithelial cells.** Am J Physiol Lung Cell Mol Physiol 2012, **302**(1):L93-L102.

92. Sewing AC, Kantores C, Ivanovska J, Lee AH, Masood A, Jain A, McNamara PJ, Tanswell AK, Jankov RP: **Therapeutic hypercapnia prevents bleomycin-induced pulmonary hypertension in neonatal rats by limiting macrophage-derived tumor necrosis factor-alpha.** Am J Physiol Lung Cell Mol Physiol 2012, **303**(1):L75-87.

93. Shigeta A, Tada Y, Wang JY, Ishizaki S, Tsuyusaki J, Yamauchi K, Kasahara Y, Iesato K, Tanabe N, Takiguchi Y, Sakamoto A, Tokuhisa T, Shibuya K, Hiroshima K, West J, Tatsumi K: **CD40 amplifies Fas-mediated apoptosis: a mechanism contributing to emphysema.** Am J Physiol Lung Cell Mol Physiol 2012, **303**(2):L141-51.

94. Smith MK, Koch PJ, Reynolds SD: **Direct and indirect roles for beta-catenin in facultative basal progenitor cell differentiation.** Am J Physiol Lung Cell Mol Physiol 2012, **302**(6):L580-94.

95. Soukup B, Benjamin A, Orogo-Wenn M, Walters D: **Physiological effect of protein kinase C on ENaC-mediated lung liquid regulation in the adult rat lung.** Am J Physiol Lung Cell Mol Physiol 2012, **302**(1):L133-9.

96. Standiford LR, Standiford TJ, Newstead MJ, Zeng X, Ballinger MN, Kovach MA, Reka AK, Bhan U: **TLR4-dependent GM-CSF protects against lung injury in Gram-negative bacterial pneumonia.** Am J Physiol Lung Cell Mol Physiol 2012, **302**(5):L447-54.

97. Sturrock A, Seedahmed E, Mir-Kasimov M, Boltax J, McManus ML, Paine R,3rd: **GM-CSF provides autocrine protection for murine alveolar epithelial cells from oxidant-induced mitochondrial injury.** Am J Physiol Lung Cell Mol Physiol 2012, **302**(3):L343-51.

98. Sutherland KM, Edwards PC, Combs TJ, Van Winkle LS: **Sex differences in the development of airway epithelial tolerance to naphthalene.** Am J Physiol Lung Cell Mol Physiol 2012, **302**(1):L68-81.

99. Tanaka K, Sato K, Aoshiba K, Azuma A, Mizushima T: **Superiority of PC-SOD to other anti-COPD drugs for elastase-induced emphysema and alteration in lung mechanics and respiratory function in mice.** Am J Physiol Lung Cell Mol Physiol 2012, **302**(12):L1250-61.

100. Tang JR, Karumanchi SA, Seedorf G, Markham N, Abman SH: **Excess soluble vascular endothelial growth factor receptor-1 in amniotic fluid impairs lung growth in rats: linking preeclampsia with bronchopulmonary dysplasia.** Am J Physiol Lung Cell Mol Physiol 2012, **302**(1):L36-46.

101. Thomas KC, Roberts JK, Deering-Rice CE, Romero EG, Dull RO, Lee J, Yost GS, Reilly CA: **Contributions of TRPV1, endovanilloids, and endoplasmic reticulum stress in lung cell death in vitro and lung injury.** Am J Physiol Lung Cell Mol Physiol 2012, **302**(1):L111-9.

102. Tropea KA, Leder E, Aslam M, Lau AN, Raiser DM, Lee JH, Balasubramaniam V, Fredenburgh LE, Alex Mitsialis S, Kourembanas S, Kim CF: **Bronchioalveolar stem cells increase after mesenchymal stromal cell treatment in a mouse model of bronchopulmonary dysplasia.** Am J Physiol Lung Cell Mol Physiol 2012, **302**(9):L829-37.

103. Tsuchiya K, Siddiqui S, Risse PA, Hirota N, Martin JG: **The presence of LPS in OVA inhalations affects airway inflammation and AHR but not remodeling in a rodent model of asthma.** Am J Physiol Lung Cell Mol Physiol 2012, **303**(1):L54-63.

104. Vaporidi K, Vergadi E, Kaniaris E, Hatziapostolou M, Lagoudaki E, Georgopoulos D, Zapol WM, Bloch KD, Iliopoulos D: **Pulmonary microRNA profiling in a mouse model of ventilator-induced lung injury.** Am J Physiol Lung Cell Mol Physiol 2012, **303**(3):L199-207.

105. Vohra PK, Hoeppner LH, Sagar G, Dutta SK, Misra S, Hubmayr RD, Mukhopadhyay D: **Dopamine inhibits pulmonary edema through the VEGF-VEGFR2 axis in a murine model of acute lung injury.** Am J Physiol Lung Cell Mol Physiol 2012, **302**(2):L185-92.

106. Wang J, Shimoda LA, Sylvester JT: **Ca2+ responses of pulmonary arterial myocytes to acute hypoxia require release from ryanodine and inositol trisphosphate receptors in sarcoplasmic reticulum.** Am J Physiol Lung Cell Mol Physiol 2012, **303**(2):L161-8.

107. Warg LA, Oakes JL, Burton R, Neidermyer AJ, Rutledge HR, Groshong S, Schwartz DA, Yang IV: **The role of the E2F1 transcription factor in the innate immune response to systemic LPS.** Am J Physiol Lung Cell Mol Physiol 2012, **303**(5):L391-400.

108. Westphalen K, Monma E, Islam MN, Bhattacharya J: **Acid contact in the rodent pulmonary alveolus causes proinflammatory signaling by membrane pore formation.** Am J Physiol Lung Cell Mol Physiol 2012, **303**(2):L107-16.

109. White LE, Santora RJ, Cui Y, Moore FA, Hassoun HT: **TNFR1-dependent pulmonary apoptosis during ischemic acute kidney injury.** Am J Physiol Lung Cell Mol Physiol 2012, **303**(5):L449-59.

110. Wu ZX, Benders KB, Hunter DD, Dey RD: **Early postnatal exposure of mice to side-steam tobacco smoke increases neuropeptide Y in lung.** Am J Physiol Lung Cell Mol Physiol 2012, **302**(1):L152-9.

111. Xu B, Chen H, Xu W, Zhang W, Buckley S, Zheng SG, Warburton D, Kolb M, Gauldie J, Shi W: **Molecular mechanisms of MMP9 overexpression and its role in emphysema pathogenesis of Smad3-deficient mice.** Am J Physiol Lung Cell Mol Physiol 2012, **303**(2):L89-96.

112. Yang S, Banerjee S, Freitas A, Cui H, Xie N, Abraham E, Liu G: **miR-21 regulates chronic hypoxia-induced pulmonary vascular remodeling.** Am J Physiol Lung Cell Mol Physiol 2012, **302**(6):L521-9.

113. Yang XR, Lin AH, Hughes JM, Flavahan NA, Cao YN, Liedtke W, Sham JS: **Upregulation of osmo-mechanosensitive TRPV4 channel facilitates chronic hypoxia-induced myogenic tone and pulmonary hypertension.** Am J Physiol Lung Cell Mol Physiol 2012, **302**(6):L555-68.

114. Yao X, Dai C, Fredriksson K, Lam J, Gao M, Keeran KJ, Nugent GZ, Qu X, Yu ZX, Jeffries N, Lin J, Kaler M, Shamburek R, Costello R, Csako G, Dahl M, Nordestgaard BG, Remaley AT, Levine SJ: **Human apolipoprotein E genotypes differentially modify house dust mite-induced airway disease in mice.** Am J Physiol Lung Cell Mol Physiol 2012, **302**(2):L206-15.

115. Yildirim E, Carey MA, Card JW, Dietrich A, Flake GP, Zhang Y, Bradbury JA, Rebolloso Y, Germolec DR, Morgan DL, Zeldin DC, Birnbaumer L: **Severely blunted allergen-induced pulmonary Th2 cell response and lung hyperresponsiveness in type 1 transient receptor potential channel-deficient mice.** Am J Physiol Lung Cell Mol Physiol 2012, **303**(6):L539-49.

**29. Annals of Thoracic Surgery**

1. Ali I, Gruenloh S, Gao Y, Clough A, Falck JR, Medhora M, Jacobs ER: **Protection by 20-5,14-HEDGE against surgically induced ischemia reperfusion lung injury in rats.** Ann Thorac Surg 2012, **93**(1):282-288.

2. Kimura N, Nakae S, Itoh S, Merk DR, Wang X, Gong Y, Okamura H, Chang PA, Adachi H, Robbins RC, Fischbein MP: **Potential role of gammadelta T cell-derived IL-17 in acute cardiac allograft rejection.** Ann Thorac Surg 2012, **94**(2):542-548.

3. Kuwabara F, Narita Y, Yamawaki-Ogata A, Kanie K, Kato R, Satake M, Kaneko H, Oshima H, Usui A, Ueda Y: **Novel small-caliber vascular grafts with trimeric Peptide for acceleration of endothelialization.** Ann Thorac Surg 2012, **93**(1):156-63; discussion 163.

4. Mishra DK, Thrall MJ, Baird BN, Ott HC, Blackmon SH, Kurie JM, Kim MP: **Human lung cancer cells grown on acellular rat lung matrix create perfusable tumor nodules.** Ann Thorac Surg 2012, **93**(4):1075-1081.

5. Nikolova A, Ablasser K, Wyler von Ballmoos MC, Poutias D, Kaza E, McGowan FX, Moses MA, Del Nido PJ, Friehs I: **Endogenous angiogenesis inhibitors prevent adaptive capillary growth in left ventricular pressure overload hypertrophy.** Ann Thorac Surg 2012, **94**(5):1509-1517.

6. Shahzad U, Li G, Zhang Y, Yau TM: **Transmyocardial revascularization induces mesenchymal stem cell engraftment in infarcted hearts.** Ann Thorac Surg 2012, **94**(2):556-562.

7. Sondergaard CS, Mathews G, Wang L, Jeffreys A, Sahota A, Wood M, Ripplinger CM, Si MS: **Contractile and electrophysiologic characterization of optimized self-organizing engineered heart tissue.** Ann Thorac Surg 2012, **94**(4):1241-8; discussion 1249.

**30. Lung Cancer**

1. Hsu HS, Lin JH, Hsu TW, Su K, Wang CW, Yang KY, Chiou SH, Hung SC: **Mesenchymal stem cells enhance lung cancer initiation through activation of IL-6/JAK2/STAT3 pathway.** Lung Cancer 2012, **75**(2):167-177.

2. Iochmann S, Lerondel S, Blechet C, Lavergne M, Pesnel S, Sobilo J, Heuze-Vourc'h N, Le Pape A, Reverdiau P: **Monitoring of tumour progression using bioluminescence imaging and computed tomography scanning in a nude mouse orthotopic model of human small cell lung cancer.** Lung Cancer 2012, **77**(1):70-76.

3. Kobayashi N, Toyooka S, Soh J, Yamamoto H, Dote H, Kawasaki K, Otani H, Kubo T, Jida M, Ueno T, Ando M, Ogino A, Kiura K, Miyoshi S: **The anti-proliferative effect of heat shock protein 90 inhibitor, 17-DMAG, on non-small-cell lung cancers being resistant to EGFR tyrosine kinase inhibitor.** Lung Cancer 2012, **75**(2):161-166.

4. Lin S, Lin CJ, Hsieh DP, Li LA: **ERalpha phenotype, estrogen level, and benzo[a]pyrene exposure modulate tumor growth and metabolism of lung adenocarcinoma cells.** Lung Cancer 2012, **75**(3):285-292.

5. Male H, Patel V, Jacob MA, Borrego-Diaz E, Wang K, Young DA, Wise AL, Huang C, Van Veldhuizen P, O'Brien-Ladner A, Williamson SK, Taylor SA, Tawfik O, Esfandyari T, Farassati F: **Inhibition of RalA signaling pathway in treatment of non-small cell lung cancer.** Lung Cancer 2012, **77**(2):252-259.

6. Mousa SA, Yalcin M, Bharali DJ, Meng R, Tang HY, Lin HY, Davis FB, Davis PJ: **Tetraiodothyroacetic acid and its nanoformulation inhibit thyroid hormone stimulation of non-small cell lung cancer cells in vitro and its growth in xenografts.** Lung Cancer 2012, **76**(1):39-45.

7. Stoyanov E, Uddin M, Mankuta D, Dubinett SM, Levi-Schaffer F: **Mast cells and histamine enhance the proliferation of non-small cell lung cancer cells.** Lung Cancer 2012, **75**(1):38-44.

8. Takata S, Takigawa N, Segawa Y, Kubo T, Ohashi K, Kozuki T, Teramoto N, Yamashita M, Toyooka S, Tanimoto M, Kiura K: **STAT3 expression in activating EGFR-driven adenocarcinoma of the lung.** Lung Cancer 2012, **75**(1):24-29.

9. Tezuka Y, Endo S, Matsui A, Sato A, Saito K, Semba K, Takahashi M, Murakami T: **Potential anti-tumor effect of IFN-lambda2 (IL-28A) against human lung cancer cells.** Lung Cancer 2012, **78**(3):185-192.

10. Xue X, Sun DF, Sun CC, Liu HP, Yue B, Zhao CR, Lou HX, Qu XJ: **Inhibitory effect of riccardin D on growth of human non-small cell lung cancer: in vitro and in vivo studies.** Lung Cancer 2012, **76**(3):300-308.
