# Supplementary material for: Anesthesia and Monitoring in Small Laboratory Mammals Used in Anesthesiology, Respiratory and Critical Care Research: A Systematic Review on the Current Reporting in Top-10 Impact Factor Ranked Journals
Source: PLoS One. 2015 Aug 25;10(8):e0134205. doi: 10.1371/journal.pone.0134205 (PMC4549323; doi:10.1371/journal.pone.0134205)
Supplement: S2 File — Contains Table A—Frequency of animal care approval, Table B—Animal characteristics and Table C—Dosage, route and application of anesthetic agents and fluid therapy. (DOCX) [file pone.0134205.s002.docx]

**Anesthesia and monitoring in small laboratory mammals used in anesthesiology, respiratory and critical care research: a systematic review on the current reporting in top-10 impact factor ranked journals**

**SUPPORTING INFORMATION File 2– Additional Results**

Christopher Uhlig, Hannes Krause, Thea Koch, Marcelo Gama de Abreu, Peter Markus Spieth*

Department of Anaesthesiology and Intensive Care Therapy, University Hospital Dresden, Technische Universität Dresden, Dresden, Germany

*C. Uhlig and H. Krause contributed equally to this paper.*

*Correspondence and reprint requests to*:

Dr. Peter Markus Spieth, Pulmonary Engineering Group, Department of Anesthesiology and Intensive Care Medicine, University Hospital Dresden, Technische Universität Dresden, Fetscherstraße 74, 01307 Dresden, Germany. Email: [peter.spieth@uniklinikum-dresden.de](mailto:mgabreu@uniklinikum-dresden.de), Tel: +4935145816006, Fax: +493514584336.

**Table A. Frequency of animal care approval**

| **Journal** | **Animal Care Approval** | | |
| --- | --- | --- | --- |
|  | **no** | **yes** | **total** |
| Pain | 11 (11.5) | 52 (82.5) | 63 (100.0) |
| Anesthesiology | 6 (8.6) | 64 (91.4) | 70 (100.0) |
| British Journal of Anaesthesia | 1 (4.5) | 21 (95.5) | 22 (100.0) |
| Anaesthesia | 0 (0.0) | 3 (100.0) | 3 (100.0) |
| Regional Anesthesia and Pain Medicine | 2 (18.2) | 9 (81.8) | 11 (100.0) |
| Anesthesia and Analgesia | 0 (0.0) | 43 (100.0) | 43 (100.0) |
| European Journal of Pain | 4 (13.3) | 26 (86.7) | 30 (100.0) |
| Minerva Anestesiologica | 0 (0.0) | 4 (100.0) | 4 (100.0) |
| American Journal of Respiratory and Critical Care Medicine | 8 (25) | 24 (75) | 32 (100.0) |
| Critical Care Medicine | 5 (8.8) | 52 (91.2) | 57 (100.0) |
| Chest | 0 (0.0) | 4 (100.0) | 4 (100.0) |
| Intensive Care Medicine | 2 (13.5) | 14 (87.5) | 16 (100.0) |
| Critical Care | 0 (0.0) | 15 (100.0) | 15 (100.0) |
| Journal of Neurotrauma | 26 (19.8) | 105 (80.2) | 131 (100.0) |
| Resuscitation | 0 (0.0) | 18 (100.0) | 18 (100.0) |
| Thorax | 1 (9.1) | 10 (90.9) | 11 (100.0) |
| European Respiratory Journal | 2 (12.5) | 14 (87.5) | 16 (100.0) |
| Journal of Heart and Lung Transplantation | 6 (42.9) | 8 (57.1) | 14 (100.0) |
| Journal of Thoracic Oncology | 3 (75.0) | 1 (25.0) | 4 (100.0) |
| American Journal of Respiratory Cell and Molecular Biology | 38 (33.6) | 75 (66.4) | 113 (100.0) |
| Respiratory Research | 2 (7.4) | 25 (92.6) | 27 (100.0) |
| Journal of Thoracic and Cardiovascular Surgery | 0 (0.0) | 9 (100.0) | 9 (100.0) |
| American Journal of Physiology – Lung Cellular and Molecular Physiology | 9 (7.8) | 106 (92.2) | 115 (100.0) |
| Annals of Thoracic Surgery | 0 (0.0) | 7 (100.0) | 7 (100.0) |
| Lung Cancer | 3 (30.0) | 7 (70.0) | 10 (100.0) |

Values are given as total number of manuscripts and percentage.

**Table B: Animal characteristics**

|  |  | **Mouse** | **Rat** | **Guinea pig** | **Rabbit** | **Total*^,^#** |
| --- | --- | --- | --- | --- | --- | --- |
| **Animal No.** |  |  |  |  |  |  |
|  | ANE | 1791 (18.0) | 7847 (78.9) | 12 (0.1) | 301 (3.0) | 9951 (43.2) |
|  | CCM | 3077 (29.9) | 6894 (67.1) | 28 (0.3) | 276 (2.7) | 10275 (44.7) |
|  | RS | 1702 (61.2) | 988 (35.5) | 0 (0.0) | 93 (3.3) | 2783 (12.1) |
|  | total | 6570 (28.5) | 15729 (68.4) | 40 (0.2) | 670 (2.9) | 23009 (1000.0) |
| **Genetic modification** | |  |  |  |  |  |
|  | WT | 252 (34.8) | 437 (60.3) | 9 (1.2) | 27 (3.7) | 725 (80.3) |
|  | KO | 139 (99.3) | 1 (0.7) | 0 (0.0) | 0 (0.0) | 140 (15.5) |
|  | KD | 14 (70.0) | 6 (30.0) | 0 (0.0) | 0 (0.0) | 20 (2.2) |
|  | KI | 17 (94.4) | 1 (0.6) | 0 (0.0) | 0 (0.0) | 18 (2.0) |
|  | total | 425 (46.6) | 451 (49.4) | 9 (1.0) | 27 (3.0) | 903 (100.0)§ |
| **Sex^#^** |  |  |  |  |  |  |
|  | m | 156 (37.5) | 321 (72.1) | 6 (66.7) | 11 (40.7) | 494 (55.1) |
|  | f | 73 (17.5) | 46 (10.4) | 2 (22.2) | 6 (22.3) | 127 (14.2) |
|  | m+f | 12 (2.9) | 18 (4.0) | 0 (0.0) | 0 (0.0) | 30 (3.3) |
|  | n/r | 175 (42.1) | 60 (13.5) | 1 (11.1) | 10 (37.0) | 246 (27.4) |
| **Age^#^** |  |  |  |  |  |  |
|  | neonatal | 5 (1.2) | 11 (2.5) | 0 (0.0) | 1 (3.7) | 17 (1.9) |
|  | young | 7 (1.7) | 21 (4.7) | 0 (0.0) | 1 (3.7) | 29 (3.2) |
|  | adolescent | 113 (27.2) | 33 (7.4) | 1 (11.1) | 3 (11.1) | 150 (16.7) |
|  | adult | 152 (36.5) | 148 (33.2) | 2 (22.2) | 9 (33.3) | 311 (34.6) |
|  | n/r | 139 (33.4) | 233 (52.2) | 6 (66.7) | 13 (48.1) | 391 (43.6) |
| **Weight^#^** |  |  |  |  |  |  |
|  | n/r | 326 (78.4) | 98 (22.0) | 1 (11.1) | 11 (40.7) | 436 (48.6) |
|  | n/s | 0 (0.0) | 5 (1.1) | 0 (0.0) | 1 (3.7) | 6 (0.7) |
|  | reported | 90 (21.6) | 342 (76.9) | 8 (88.9) | 15 (55.6) | 455 (50.7) |
|  | weight min [g] | 24,2±19,2 | 249±71 | 306±78 | 2719±534 |  |
|  | weight max [g] | 30,1±23,9 | 320±187 | 450±133 | 3307±701 |  |

Values are given as number (%) except of weight min and max (mean ± standard deviation). ANE: category Anesthesiology, CCM: Category Critical Care Medicine, RS: Category Respiratory System according to ISI Web of Knowledge. WT: wild type, KO: knock out, KD: knock down, KI: knock in, m: male, f: female, m+f: male and female, min: minimum, max: maximum, n/r: not reported, n/s: not specified,§: total number varying from 897 since more than one genetic modification could be applied per species in one manuscript, * denominator of the respective row, #: percentage given per column.

**Table C: Dosage, route and application of anesthetic agents and fluid therapy**

|  |  | **Premedication** | | **Maintenance** | | **NMBA** | | **Postoperative** | **Fluid** |
| --- | --- | --- | --- | --- | --- | --- | --- | --- | --- |
|  |  | **Sedation** | **Analgesia** | **Sedation** | **Analgesia** | **Bolus** | **Maintenance** | **Analgesia** |  |
| **Dosage** |  |  |  |  |  |  |  |  |  |
|  | n/r | 10 (5.9) | 4 (12.5) | 154 (22.3) | 30 (16.2) | 12 (31.6) | 31 (81.6) | 3 (8.1) | 7 (10.9) |
|  | reported | 147 (87.0) | 26 (81.3) | 449 (65.1) | 149 (80.5) | 28 (73.8) | 7 (18.4) | 30 (81.1) | 58 (90.6) |
|  | n/s | 12 (7.1) | 2 (6.3) | 87 (12.6) | 6 (3.2) | 1 (2.6) | 0 (0.0) | 4 (10.8) | 1 (1.5) |
|  | total | 169 (100.0) | 32 (100.0) | 690 (100.0) | 185 (100.0) | 38 (100.0) | 38 (100.0) | 37 (100.0) | 66 (100.0) |
| **Route** |  |  |  |  |  |  |  |  |  |
|  | n/r | 22 (13.0) | 8 (25.0) | 110 (15.9) | 43 (43.0) | 19 (50.0) | 32 (84.2) | 7 (18.9) | 7 (10.9) |
|  | n/s | 0 (0.0) | 0 (0.0) | 1 (0.1) | 0 (0.0) | 0 (0.0) | 0 (0.0) | 0 (0.0) | 0 (0.0) |
|  | inhal | 80 (47.3) | 0 (0.0) | 285 (41.5) | 0 (0.0) | 0 (0.0) | 0 (0.0) | 0 (0.0) | 0 (0.0) |
|  | ip | 54 (32.0) | 12 (37.5) | 225 (32.6) | 102 (55.1) | 11 (28.9) | 1 (2.6) | 3 (8.1) | 13 (20.3) |
|  | iv | 5 (3.0) | 1 (3.1) | 59 (8.5) | 14 (7.6) | 5 (13.2) | 5 (13.2) | 0 (0.0) | 31 (20.3) |
|  | sc | 1 (0.6) | 2 (6.3) | 3 (0.4) | 16 (8.6) | 0 (0.0) | 0 (0.0) | 26 (70.3) | 0 (0.0) |
|  | im | 7 (4.1) | 9 (28.1) | 7 (1.0) | 9 (4.9) | 1 (2.6) | 0 (0.0) | 1 (2.7) | 0 (0.0) |
|  | iart | 0 (0.0) | 0 (0.0) | 0 (0.0) | 0 (0.0) | 2 (5.3) | 0 (0.0) | 0 (0.0) | 2 (3.1) |
|  | ioc | 0 (0.0) | 0 (0.0) | 0 (0.0) | 1 (0.5) | 0 (0.0) | 0 (0.0) | 0 (0.0) | 0 (0.0) |
|  | total | 169 (100.0) | 32 (100.0) | 690 (100.0) | 185 (100.0) | 38 (100.0) | 38 (100.0) | 37 (100.0) | 64 (100.0) |
| **Application** |  |  |  |  |  |  |  |  |  |
|  | n/r | 20 (11.8) | 9 (28.1) | 109 (15.8) | 42 (22.7) | 24 (63.2) | 31 (81.6) | 13 (35.1) | 16 (25.0) |
|  | n/s | 0 (0.0) | 0 (0.0) | 10 (1.4) | 3 (1.6) | 0 (0.0) | 0 (0.0) | 3 (8.1) | 1 (1.5) |
|  | cont | 80 (47.3) | 0 (0.0) | 302 (43.8) | 16 (8.6) | 0 (0.0) | 5 (13.2) | 0 (0.0) | 23 (35.9) |
|  | cont+bolus | 0 (0.0) | 0 (0.0) | 15 (2.2) | 0 (0.0) | 0 (0.0) | 0 (0.0) | 0 (0.0) | 0 (0.0) |
|  | bolus | 69 (40.8) | 23 (71.9) | 254 (36.8) | 124 (67.0) | 14 (36.8) | 2 (5.2) | 21 (56.8) | 26 (40.6) |
|  | total | 169 (100.0) | 32 (100.0) | 690 (100.0) | 185 (100.0) | 38 (100.0) | 38 (100.0) | 37 (100.0) | 64 (100.0) |

Values are given as number (% relative to total). NMBA: neuromuscular blocking agents, n/r: not reported, n/s: not specified, inhal: inhalative, ip: intraperitoneal, iv: intravenous, sc: subcutaneous, im: intramuscular, iart: intraarterial, ioc: intraocular, cont: continuous.
